# Anti-cancer and immunomodulatory evaluation of new nicotinamide derivatives as potential VEGFR-2 inhibitors and apoptosis inducers: *in vitro* and *in silico* studies

**DOI:** 10.1080/14756366.2022.2110868

**Published:** 2022-08-18

**Authors:** Reda G. Yousef, Alaa Elwan, Ibraheem M. M. Gobaara, Ahmed B. M. Mehany, Wagdy M. Eldehna, Souad A. El-Metwally, Bshra A. Alsfouk, Eslam B. Elkaeed, Ahmed M. Metwaly, Ibrahim H. Eissa

**Affiliations:** a Pharmaceutical Medicinal Chemistry & Drug Design Department, Faculty of Pharmacy (Boys), Al-Azhar University, Cairo, Egypt; b Zoology Department, Faculty of Science (Boys), Al-Azhar University, Cairo, Egypt; c Department of Pharmaceutical Chemistry, Faculty of Pharmacy, Kafrelsheikh University, Kafrelsheikh, Egypt; d Department of Basic Science, Higher Technological institute, 10th of Ramadan City, Egypt; e Department of Pharmaceutical Sciences, College of Pharmacy, Princess Nourah Bint Abdulrahman University, Riyadh, Saudi Arabia; f Department of Pharmaceutical Sciences, College of Pharmacy, AlMaarefa University, Riyadh, Saudi Arabia; g Pharmacognosy and Medicinal Plants Department, Faculty of Pharmacy (Boys), Al-Azhar University, Cairo, Egypt; h Biopharmaceutical Products Research Department, Genetic Engineering and Biotechnology Research Institute, City of Scientific Research and Technological Applications (SRTA-City), Alexandria, Egypt

**Keywords:** Anticancer, nicotinamide, VEGFR-2 inhibition, cell cycle, apoptosis, immunomodulatory

## Abstract

New nicotinamide derivatives **6**, **7**, **10**, and **11** were designed and synthesised based on the essential features of the VEGFR-2 inhibitors. Compound **10** revealed the highest anti-proliferative activities with IC_50_ values of 15.4 and 9.8 µM against HCT-116 and HepG2, respectively compared to sorafenib (IC_50_ = 9.30 and 7.40 µM). Compound **7** owned promising cytotoxic activities with IC_50_ values of 15.7 and 15.5 µM against the same cell lines, respectively. Subsequently, the VEGFR-2 inhibitory activities were assessed for the titled compounds to exhibit VEGFR-2 inhibition with sub-micromolar IC_50_ values. Moreover, compound **7** induced the cell cycle cessation at the cycle at %G2-M and G0-G1phases, and induced apoptosis in the HCT-116. Compounds **7** and **10** reduced the levels of TNF-*α* by 81.6 and 84.5% as well as IL-6 by 88.4 and 60.9%, respectively, compared to dexamethasone (82.4 and 93.1%). *In silico* docking, molecular dynamics simulations, ADMET, and toxicity studies were carried out.

## 1. Introduction

Cancer is a deadly illness characterised by an overexcited and uncontrolled cell division with the plausibility to spread and attack other parts of the human body^1^. Whereas advance has been made in the treatment and avoidance, the worldwide burden of cancer and mortality is expanding colossally. Epidemiological ponders uncovered that cancer accounts for one of each five deaths^2^.

Cancer cells are characterised by biochemical anomalies. Cancer cells need more oxygen and supplements to outlive and multiply; consequently, they must be close to blood vessels to have availability to the blood circulation system^3^. Angiogenesis, the growth of new blood vessels from pre-existing vasculatures, is a fundamental factor in cancer development^4^. The angiogenesis is managed by assortments of protein kinases^5^. Among these proteins, the vascular endothelial growth factor (VEGF) is one of the foremost strong angiogenic controlling variables^6^^,^^7^.

VEGFs employ their cancer development consequences through the interaction with the three kinase receptors (VEGFRs 1, 2, and 3)^8^. Among them, VEGFR-2 is the subtype that is responsible for angiogenesis^9^. Consequently, the inhibition VEGF/VEGFER-2 pathway is an efficacious and desired target to control cancerous cells^10^. Interestingly, several compounds that inhibited VEGFR-2 displayed an additional potential to induce apoptosis in the cancerous, which causes a very strong antitumor effect^11^.

**Figure 1.**
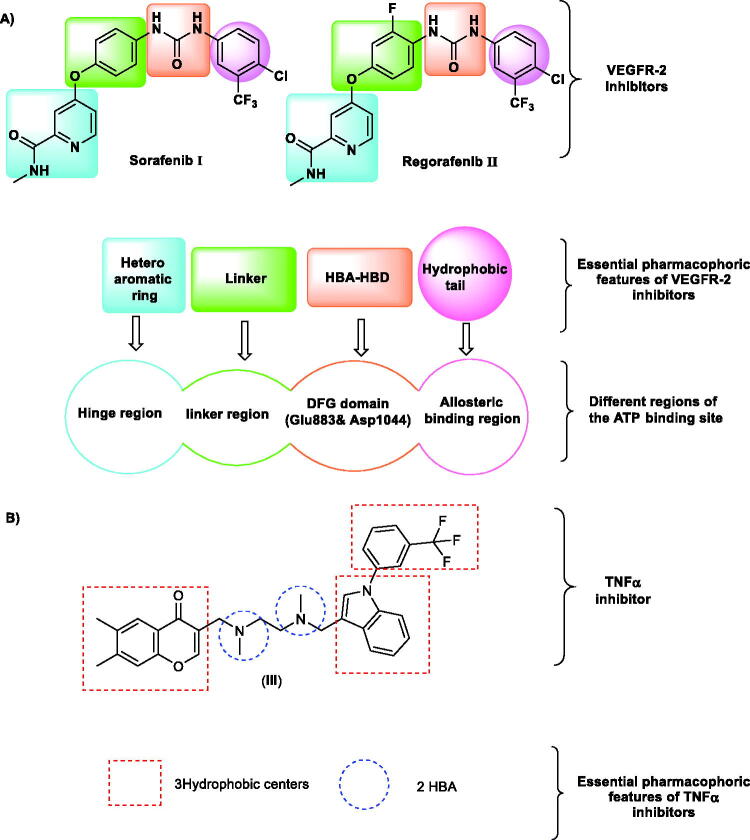
(A) Some reported VEGFR inhibitors showing the essential pharmacophoric features that occupy the different regions of the ATP binding site. (B) reported TNF*α* inhibitor with its pharmacophoric features.

Due to the hydrophobic nature of the VEGFR-2 binding site, VEGFR-2 inhibitors displayed a vast diversity of chemical structures^12^. However, the chemical structures of the two well-known inhibitors (sorafenib **I** and regorafenib **II**) exhibited some common features that are essential for any inhibitor to bind with the active binding site correctly (Figure 1). These structural features a heteroaromatic ring system, a central linker, a pharmacophore moiety, and a hydrophobic tail that should interact with the hinge region^13^, the linker region^14^, the DFG motif region, and the allosteric pocket^15^, respectively.

Cytokines have an important function in cancer progression and spread. Cancer patients have been found to have elevated levels of various cytokines. Anti-cytokine medicines are being researched extensively, potentially opening up new treatment choices for symptoms that are now difficult to manage^16^. Tumour necrosis factor (TNF-α) and interleukin-6 (IL-6) are multifunctional cytokines implicated in tumour growth and metastasis^17^. The IL-6 is a pleiotropic cytokine that plays a significant role in the growth and differentiation of cells. Several studies have addressed the role of IL-6 in tumour cell growth *in vitro*^18^. TNF-α is one of the key chemical mediators implicated in inflammation-associated cancers. There is now substantial evidence that TNF-α is involved in the promotion and progression of experimental and human cancers^19^. Accordingly, anticancer agents that have an inhibitory effect on both TNF-α and IL-6 are advantageous for drug discovery.

Nicotinamide nucleus was reported as a potent modulator of several proinflammatory cytokines and has a potent immunomodulatory effect *in vitro*, and may have great potential for the treatment of human inflammatory diseases^20^. Additionally, nicotinamide moiety was reported to have potential as an anticancer^21^.

Through our teamwork’s trip within the revel of novel anticancer agents^22^ in particular VEGFR-2 inhibitors^9^^,^^23^, It is of intrigued to start a new research towards the discovery of new nicotinamide derivatives as VEGFR-2 inhibitors with immunomodulatory effects. In this research, the designed nicotinamide derivatives are modified analogs of sorafenib and have the essential pharmacophoric features of VEGFR-2 inhibitors.

### 1.1. Rationale and design

**Figure 2.**
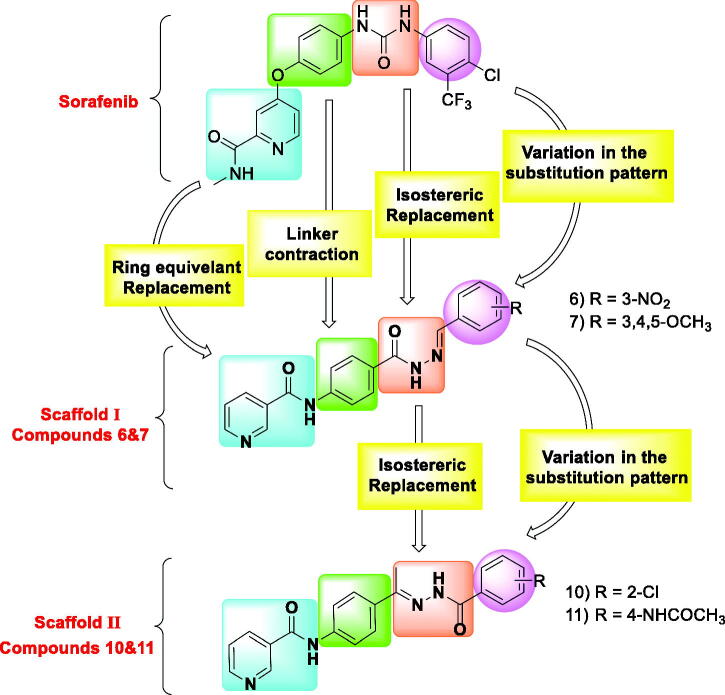
Strategies of molecular design.

In the present work, sorafenib was used as a lead compound to design new derivatives with the same pharmacophoric features of VEGFR-2 inhibitors. Four modification strategies were carried out at the four pharmacophoric features of sorafenib. Firstly, the *N*-methylpicolinamide moiety of sorafenib was replaced by nicotinamide moiety via ring equivalent modification strategy. Second, the linker (phenoxy) moiety of sorafenib was subjected to liker contaction to be a phenyl group in the designed compounds. Third, the pharmacophore (urea moiety) of sorafenib was replaced by two different hydrazone derivatives. Fourth, the hydrazone derivatives are conserving the hydrogen bond donor and hydrogen bond acceptor atoms that can form the essential hydrogen bonding interactions at the DFG motif region. Simultaneously, the hydrophobic head of sorafenib was subjected to the strategy of variation in the substitution pattern to study the electronic and hydrophobic effect on biological activities (Figure 2).

In many publications, nicotinamide moiety was reported as potent inhibitor of proinflammatory cytokines (TNF*α* and IL-6)^20^^,^^24^^,^^25^. These reports revealed that nicotinamide has a potent immunomodulatory activity and may have promising effect for the treatment of inflammatory disease as cancer^20^. In addition, compound **III** (The co-crystallised ligand of TNF*α* crystal structure (PDB ID: 2AZ5) was confirmed to have fitting interaction in the active pocket of TNF*α*)^26^. The reported pharmacophoric features of TNF*α* inhibitors include three hydrophobic centres with two hydrogen bond acceptors^27^. From these findings, it was obvious that our nicotinamide derivatives have the same structural similarity and pharmacophoric features of cytokines inhibitors which drove us to validate the synthesised compounds as immunomodulators besides its VEGFR-2 inhibitory effect (Figure 1).

All targeted compounds were screened for their *in vitro* antiproliferative effect against two cancer cell lines in addition to the examination of their VEGFR-2 inhibitory activity. Apoptosis and cell cycle were evaluated for a representative compound. Next, the effects of the most active derivatives on the levels of TNF-α and IL-6 were determined. Different *in silico* (docking, MD simulations, MM-PBSA, ADMET, and toxicity) studies were conducted to assess the binding pattern of the synthesised compounds and their stability in the active pocket of VEGFR-2, TNF*α*, and IL-6.

## 2. Results and discussion

### 2.1. Chemistry

**Scheme 1.**
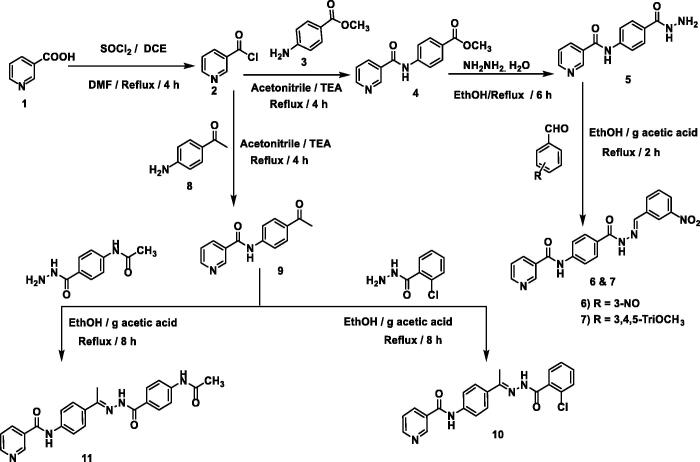
Synthetic route of target compounds **6, 7, 10,** and **11.**

The synthetic pathways for the target compounds were demonstrated in Schemes 1. Firstly, heating of nicotinic acid **1** with thionyl chloride in 1,2 dichloroethane and a catalytic amount of DMF furnished nicotinoyl chloride **2**^28^. Refluxing the nicotinoyl chloride **2** with 4-aminomethylbenzoate **3** in acetonitrile in the presence of triethylamine afforded the corresponding methyl ester derivatives *N*-(4-acetylphenyl)nicotinamide **4**^28^, which treated with hydrazine hydrate to produce *N*-(4-(hydrazinecarbonyl)-phenyl)nicotinamide **5**^29^. The produced hydrazide derivative **5** was then allowed to react with appropriate aldehydes namely 3-nitrobenzaldehyde, and 3,4,5-trimethoxy benzaldehyde to afford scaffold-I compounds **6** and **7,** respectively (Scheme 1).

Additionally, the nicotinoyl chloride **2** was further allowed to react with the commercially available *p*-aminoacetophenone **8** to yield the equivalent *N*-(4-acetylphenyl)nicotinamide **9**^30^. The latter was refluxed in ethanol and a catalytic amount of glacial acetic acid with appropriate amines namely, 2-chlorobenzohydrazide and *N-*(4-(hydrazinecarbonyl)phenyl)acetamide to give compounds of scaffold-II (compounds **10** and **11**), respectively.

The structures of the synthesised compounds were confirmed by ^1^H NMR, which showed the presence of two downfield singlet signals at the range of 10.49–12.08 ppm attributed to the NH protons. The integration of aromatic and olefinic protons was increased at the aromatic region corresponding to the additional aromatic ring. For scaffold-I (compounds **6** and **7**), the characteristic aldehydic proton (-CH = N) appeared as a singlet peak in aromatic protons. For scaffold-II, the characteristic CH_3_ of the hydrazone moiety appeared as upfield singlet signals at range of 2.3 to 3.46 ppm. IR charts of the synthesised compounds revealed the presence of NH stretching bands in the range of 3448 and 3174 cm^−1^. Moreover, ^13 ^C NMR spectra were also consistent with the assigned structures of the synthesised compounds. The ^13 ^C NMR spectra exhibited increased integration of the aromatic region, they also showed the appearance of upfield aliphatic protons corresponding to compounds **7**, **10**, and **11** at a range of 60.30–13.93 ppm.

### 2.2. Biological testing

#### 2.2.1. *In vitro* anti-proliferative activities

The *in vitro* anti-proliferative activities of the synthesised compounds **6**, **7**, **10**, and **11** were assessed against two human cancer cell lines (HCT-116; colorectal rectal cancer, and HepG-2; hepatocellular carcinoma) using the MTT assay^9^ and maintaining Sorafenib as a reference molecule. The concentrations of the tested compounds that produced 50% growth inhibition of cancer cells (IC_50_) are presented in Table 1.

**Table 1.** *In vitro* cytotoxic activities of the target compounds against **HCT-116** and **HepG-2** cell lines and *in vitro* inhibitory activities against VEGFR-2.

Comp.	Cytotoxicity IC_50_ (µM)		VEGFR-2 IC_50_ (nM)
	HCT-116	HepG-2	
**6**	22.09 ± 0.064	19.50 ± 0.063	291.9
**7**	15.70 ± 0.058	15.50 ± 0.055	250.2
**10**	15.40 ± 0.056	9.80 ± 0.05	145.1
**11**	20.17 ± 0.063	21.60 ± 0.07	86.60
**Sorafenib**	9.30 ± 0.201	7.40 ± 0.253	53.65

From the results, all the tested **6**, **7**, **10**, and **11** compounds exhibited promising cytotoxic effects on HCT-116 cell lines with IC_50_ values of 22.09, 15.70, 15.40, and 20.17 µM, respectively, when compared to the positive control, Sorafenib (IC_50_ = 9.30 μM). Regarding the cytotoxicity against HepG-2, compound **10** showed potent activity displaying an IC_50_ value of 9.80 μM that was comparable to sorafenib (IC_50_ = 7.40 μM). Compounds **6**, **7**, and **11** showed moderate activities with IC_50_ values of 19.50, 15.50, and 21.60 μM, respectively.

On analysing the SAR of scaffold I derivatives, the synthesised compound bearing 1,2,3-trimethoxybenzene moiety (compound **7**) as a hydrophobic tail showed higher activity than that with nitrobenzene moiety (compound **6**). For scaffold II derivatives, the synthesised compound bearing chlorobenzene moiety (compound **10**) as a hydrophobic tail showed higher activity than that with N-phenylacetamide moiety (compound **11**).

#### 2.2.2. *In vitro* VEGFR-2 enzyme assay inhibition

The synthesised compounds were investigated for their VEGFR-2 inhibitory activity. Sorafenib was used as a reference molecule, and the results were summarised in Table 1. Compounds **10** and **11** effectively inhibited VEGFR-2 activity with IC_50_ values of 145.1 and 86.60 nM relative to Sorafenib (IC_50_ = 53.65 nM). Compounds **6** and **7** displayed weak VEGFR-2 inhibitory activity (IC_50_ = 291.9 and 250.2 nM, respectively). Analysing assessment results indicated that the simplification approach may be beneficial for VEGFR-2 inhibitory activity as indicated by the sub-micromolar level of inhibition.

#### 2.2.3. Cell cycle analysis

**Figure 3.**
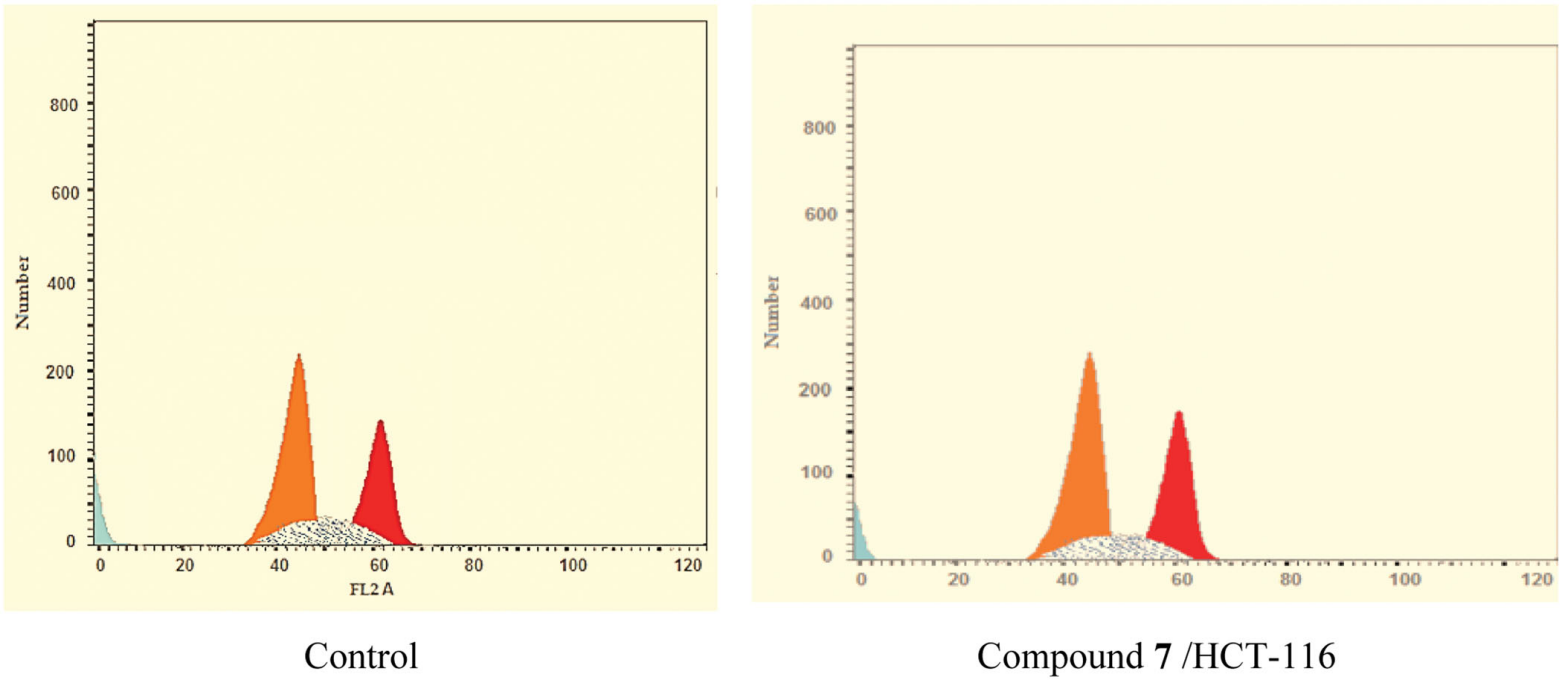
Cell cycle phases after the treatment of HCT-116 Cells with compound **7**.

**Figure 4.**
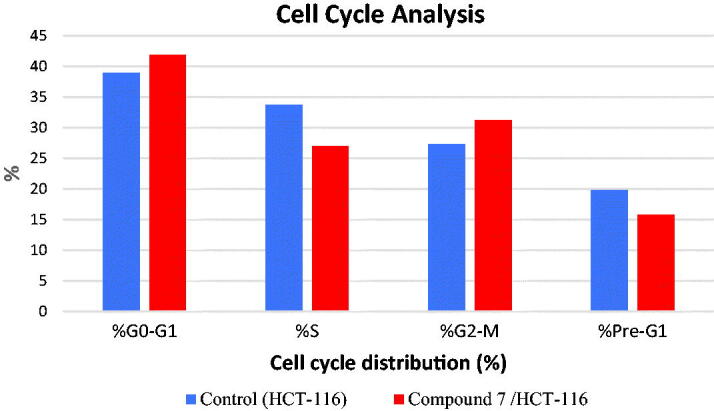
Flow cytometric analysis of cell cycle phases after treatment with compound **7**.

Numerous anti-proliferative agents produce their effect by either arresting the cell growth at a particular point of the cell cycle or the induction of apoptosis, or by a combined effect^31^. Taking compound **7** as a representative example of the tested compounds, its effect on the cell-cycle arrest of the HCT-116 cells for 48 h was evaluated by flow cytometry assay using the untreated HCT-116 as a control. Compared to the control group, compound **7** produced an increase in the HCT-116 population through the G2-M phase (from 27.29% to 31.16%) and the G0-G1 phase (from 38.96% to 41.92%). Such results indicated that compound **7** could arrest the cell cycle at %G2-M and G0-G1phases (Table 2, Figures 3 and 4).

**Table 2.** Effect of compound **7** on cell cycle phases of HCT-116 cells

Sample	Cell cycle distribution (%)			
	%G0-G1	% S	%G2-M	%Pre-G1
HCT-116	38.96	33.75	27.29	19.82
Compound **7** /HCT-116	41.92	26.92	31.16	15.74

#### 2.2.4. Induction of apoptosis

To determine whether apoptosis induction is involved in the anti-proliferative effects of the synthesised compounds, the apoptosis assay was performed for compound **7** against HCT-116 cells. The cells were incubated with either vehicle or compound **7** at a concentration of 15.70 μM for 48 h and then stained with FITC-Annexin V and propidium iodide (PI). The percentages of apoptotic HCT-116 cells were determined by flow cytometry analysis.

**Figure 5.**
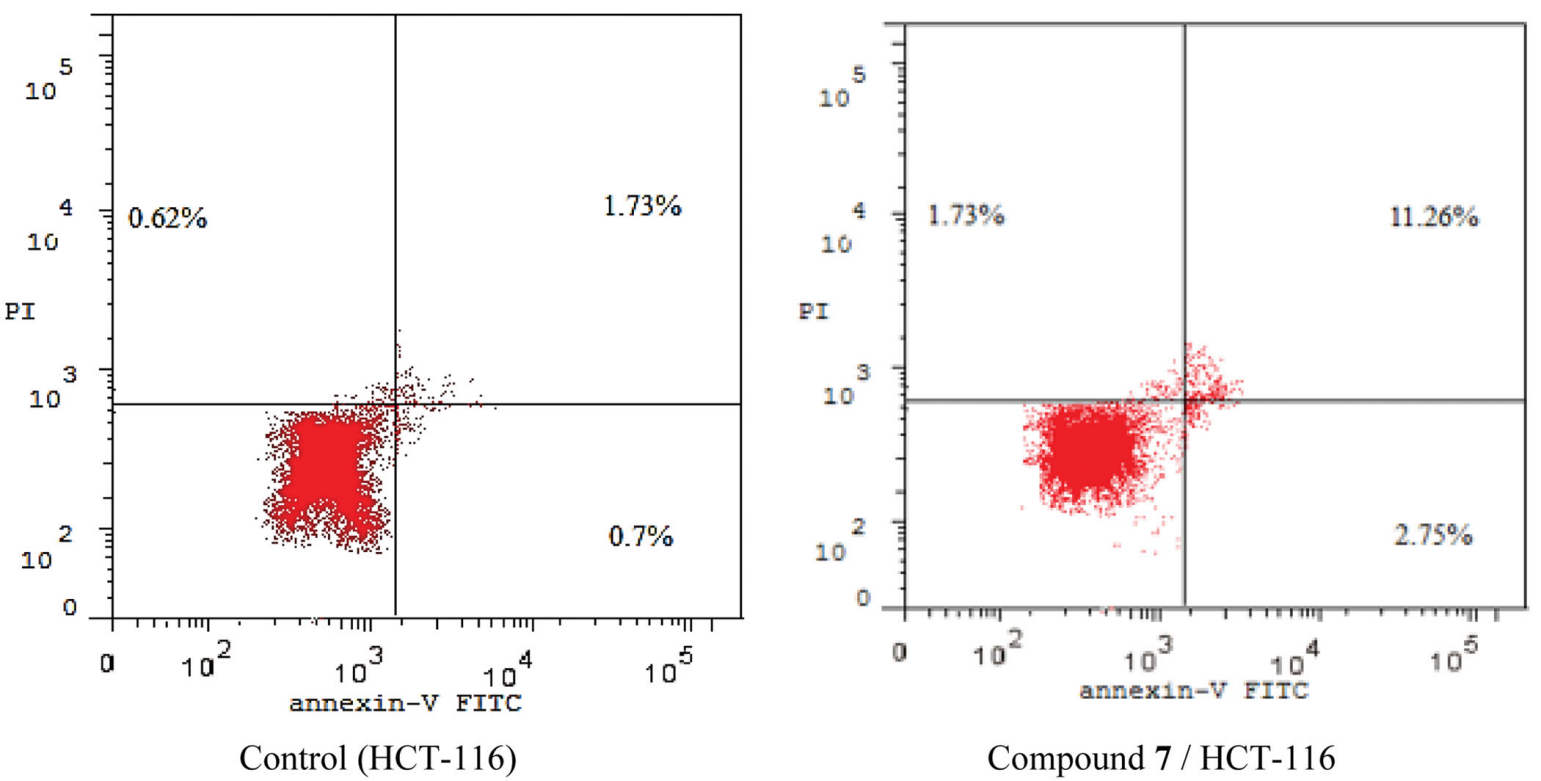
Apoptosis effect of compound **7** in HCT-116 cells.

**Figure 6.**
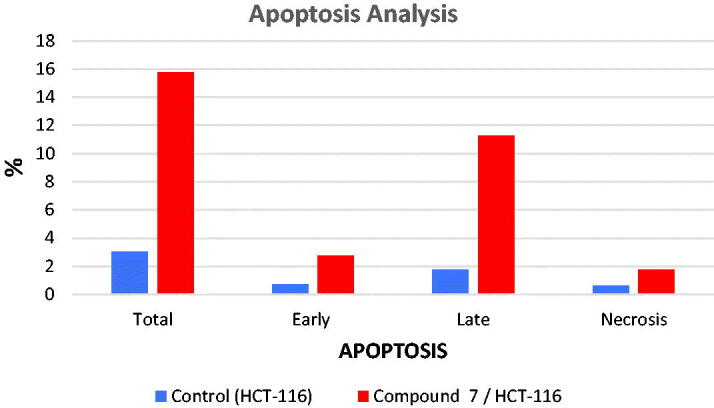
Flow cytometric analysis of apoptosis in HCT-116 cells exposed to compound **7**.

The results are shown in Table 3 and Figures 5 and 6. In the vehicle (control) group, the occurrence of HCT-116 cell apoptosis was minimal. On the other hand, in the compound **7**-treated HCT-116 cells, the population of the apoptotic cells clearly increased (5-folds of total apoptosis induction). In the early phase, compound **7** produces a 4-fold increase in the apoptotic cells (from 0.7 to 2.75%). In the late phase, it induced 6.5-folds in the apoptotic cells (from 1.73 to 11.26%). These results revealed that the antitumor activity of compound **7** is associated with the apoptosis induction effect.

**Table 3.** Stages of the cell death process in HCT-116 cells after treatment with compound **7**

	Apoptosis			Necrosis
Sample	Total	Early	Late	
Control (HCT-116)	3.05	0.7	1.73	0.62
Compound **7** / HCT-116	15.74	2.75	11.26	1.73

#### 2.2.5. Effects on the levels of the immunomodulatory proteins (TNF-α and IL-6)

The effect of the two most active compounds (**7** and **10**) on the level of immunomodulatory proteins (TNF-α and IL-6) was determined using qRT-PCR. In this test, HCT-116 cells were treated with compounds **7** and **10** at the concentrations of 15.70 and 15.40 µM, respectively for 24 h. Dexamethasone as a universal immunomodulatory drug was used as a reference molecule.

As presented in Table 4, compounds **7** and **10** showed a marked decrease in the levels of NF-α with inhibition percentages of 81.64 and 84.52%, respectively. These values are comparable with that of dexamethasone (82.47%). Regarding the IL-6, compounds **7** and **10** showed promising but less decreasing effects (88.44 and 60.98%, respectively) than dexamethasone (93.15%).

**Table 4.** Effect of compounds **7** and **10** on the levels of TNF-α and IL-6 in HCT-116 cells.

Sample	TNF-α (% inhibition)	IL-6 (% inhibition)
Compound **7**	81.64	88.44
Compound **10**	84.52	60.98
Dexamethasone	82.47	93.15

### 2.3. *In silico* studies

#### 2.3.1. Molecular docking against VEGFR-2 active pocket

Molecular docking simulation provides insight into the binding interaction and affinity between the compound and the receptor^32^. Promising biological activity is expected by a higher binding energy and a similar binding mode to that of the reference ligand^32^. To study the binding characteristics of the newly synthesised compounds in the binding site of VEGFR-2, molecular docking studies were performed by Molecular Operating Environment (MOE. 14) software. The X-ray crystallographic structure of VEGFR-2 co-crystallised with sorafenib was downloaded from the Protein Data Bank (PDB ID: 4ASD) ^21^. The docking process was initially validated by re-docking the co-crystallised ligand inside the active pocket of the target protein (Supplementary Data). As reported, the low root mean square deviation (RMSD) value (**≤**2.0 Å) of the best-docked conformer of the bound ligand in the experimental crystal indicates the used scoring function is successful^33^. In the current study, the RMSD between the co-crystallised conformer and the re-docked one is 0.65 Å. This indicates the appropriateness of the utilised molecular docking protocol. The docking scores of the tested ligands were summarised in Table 5, and their binding features inside the active site of the target protein were depicted. The binding poses with the highest energy scores were selected for analysis. The output from MOE software was further visualised using Discovery Studio 4.0 software.

**Table 5.** The calculated ΔG (binding free energies) of the synthesised compounds and reference drug sorafenib against VEGFR-2 (ΔG in Kcal/mole).

Compound	ΔG [Kcal/mole]	Compound	ΔG [Kcal/mole]
**6**	−16.41	**11**	−19.30
**7**	−22.85	**Sorafenib**	−36.20
**10**	−19.73		

**Figure 7.**
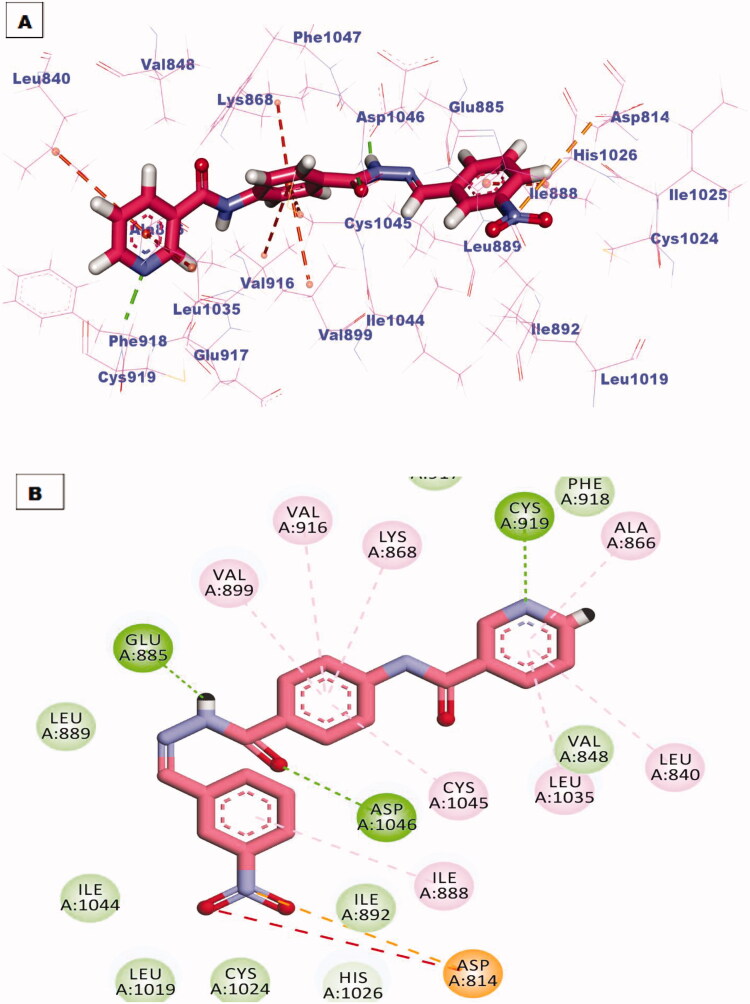
(A) 3D binding mode of compound **6** into VEGFR-2 active site, (B) 2D binding mode of compound **6** into VEGFR-2 active site.

All the newly synthesised compounds exhibited an interesting binding mode like that of sorafenib (Supplementary Data). The designed compound **6** bind to the receptor exhibiting a binding affinity of −16.41 kcal/mol. In its binding manner, the pyridinyl group occupied the hinge region forming a hydrogen bond interaction with an essential amino acid Cys919. Additionally hydrophobic interaction formed between this head and amino acid residue Leu840. The phenyl ring spacer was stabilised in the gatekeeper district through four hydrophobic interactions with Val916, Val899, Lys868, and Cys1945. However, the pharmacophore hydrazone moiety achieved its required job by binding to the key amino acids Glu885 and Asp1046 in the DGF motif. Moreover, the terminal p-nitrophenyl hydrophobic tail completely fits in the allosteric site forming one hydrophobic interaction with Ile 888 and two electrostatic interactions through the nitro group with amino acid residue Asp814 (Figure 7).

**Figure 8.**
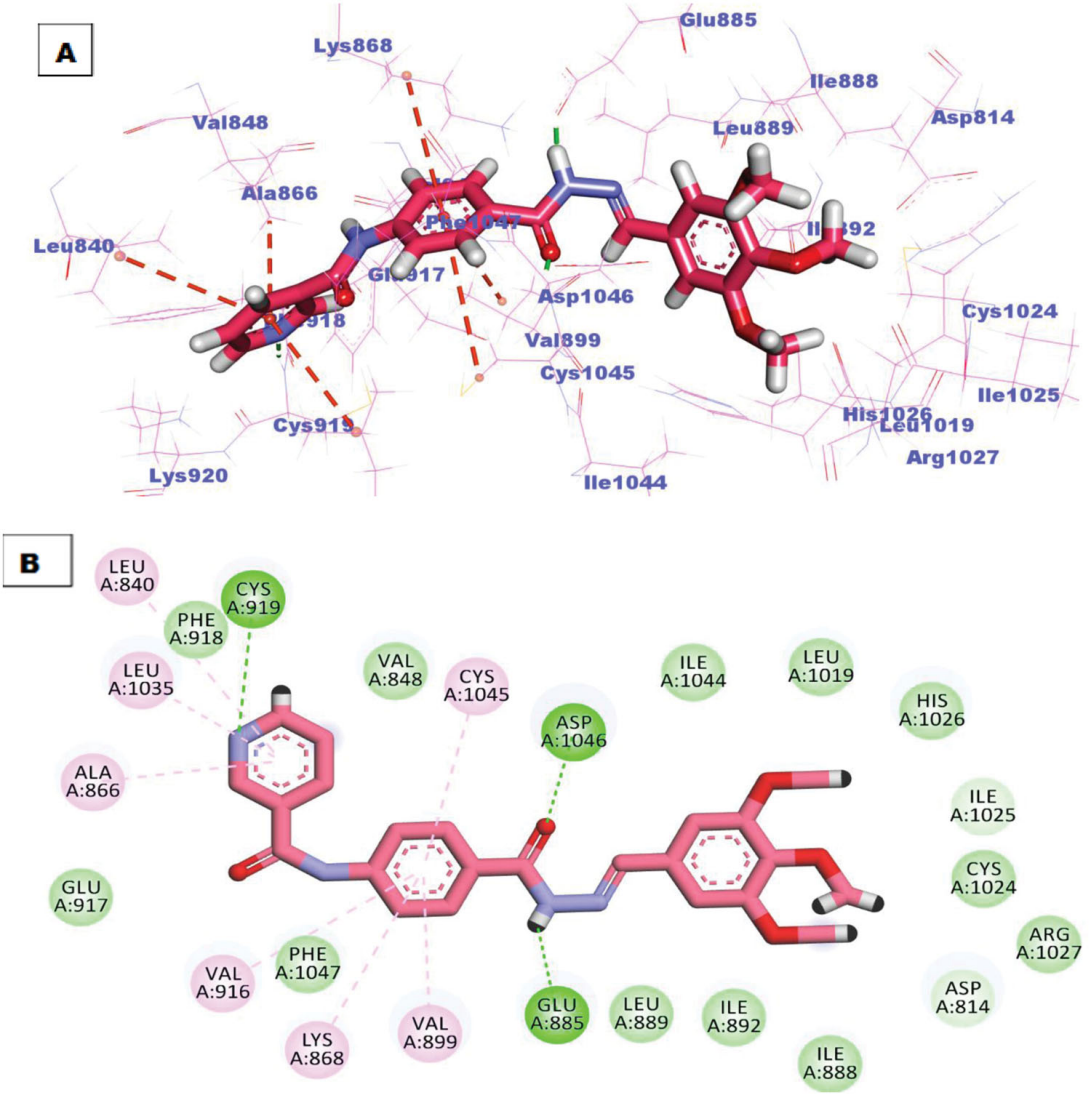
(A) 3D binding mode of compound **7** into VEGFR-2 active site, (B) 2D binding mode of compound **7** into VEGFR-2 active site.

The best-scored pose (−22.85 kcal/mol) of compound **7** mimicked the key interactions of the co-crystallised ligand; sorafenib. It kept the hydrogen bonding interaction with the essential amino acid Cys919 in the hinge region via the nitrogen atom of the pyridine ring. The pyridine head was much more stabilised by many hydrophobic interactions generated with Leu840, Leu1035, and Ala866. Moreover, the phenyl linker was enclosed in the gatekeeper area through four hydrophobic interactions with Val899, Val916, Lys868, and Cys1045. Furthermore, the hydrazone moiety interacted as an H-bond donor and acceptor producing essential hydrogen bonding interactions with Glu883 and Asp1044 at the DGF motif. Finally, trimethoxyphenyl tail occupied the hydrophobic allosteric site (Figure 8).

#### 2.3.2. Molecular docking against immunomodulatory proteins (TNF-α and IL-6)

**Figure 9.**
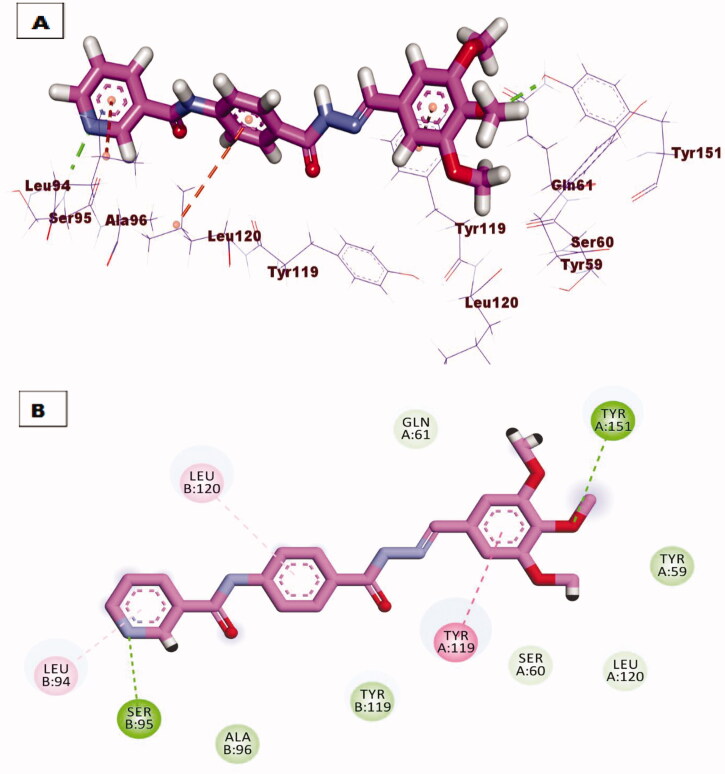
(A) 3D binding mode of compound **7** against TNF-*α* protein, (B) 2D binding mode of compound **7** against TNF-*α* protein.

**Figure 10.**
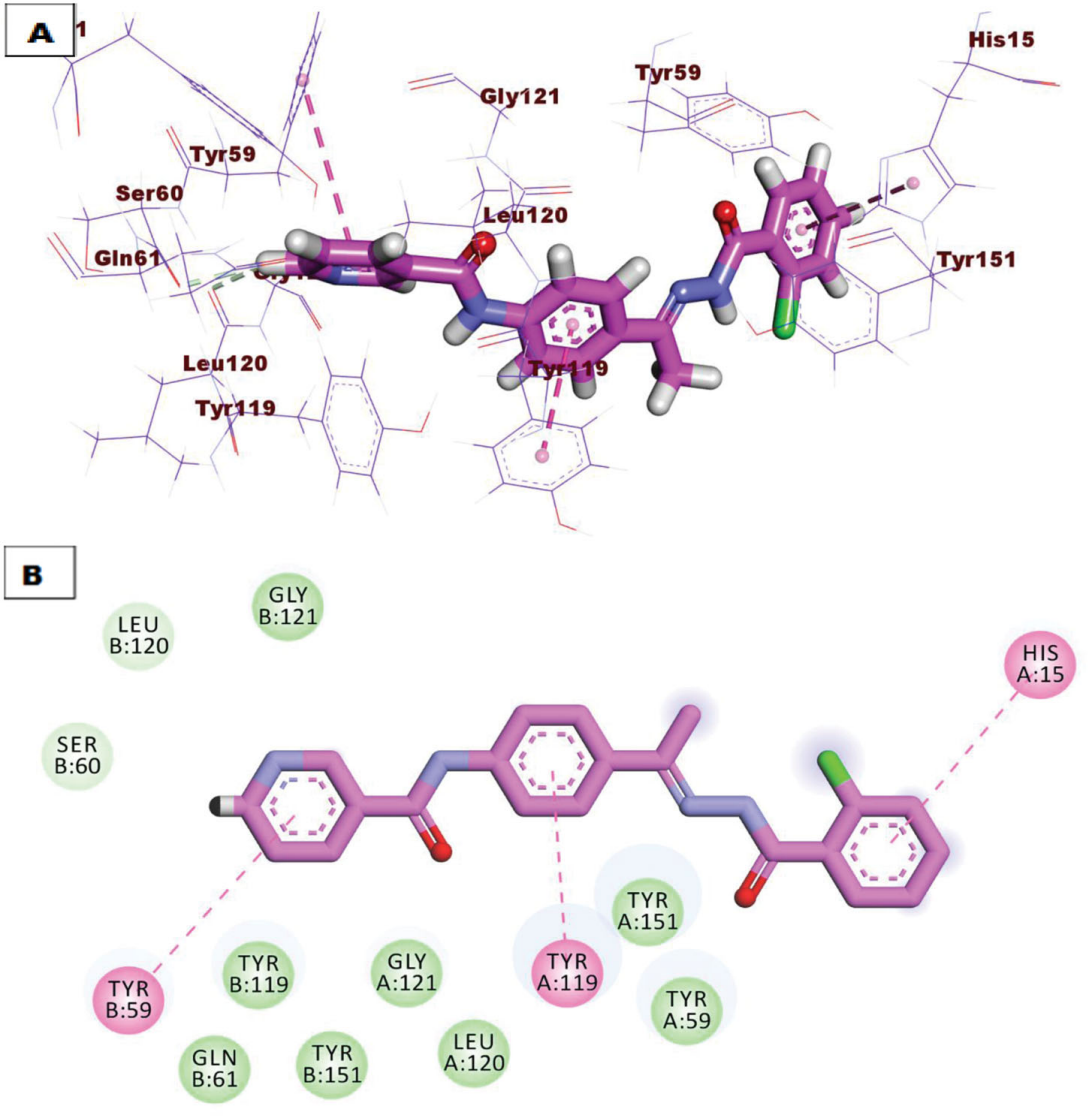
(A) 3D binding mode of compound **10** against TNF-*α* protein, (B) 2D binding mode of compound **10** against TNF-*α* protein.

Due to promising results of biological testing of the synthesised compounds on the level of immunomodulatory proteins (TNF-α and IL-6). An additional docking study was carried out to investigate the binding pattern of the most active compounds **7** and **10** against (TNF-α and IL-6) proteins. The crystal structures of the target proteins were downloaded from the Protein Data Bank (PDB ID: 2AZ5 for TNF-α) and (PDB ID: 1ALU for IL-6)^34^. The receptor protein was prepared for molecular docking simulation by removing ligand and water from the active site, and by addition of polar hydrogens. After the preparation of ligands, docking of the selected compounds have proceeded following the valid protocol. The results obtained after molecular docking against TNF-*α* indicated that compound **7** showed a promising binding mode similar to that of the small inhibitor molecule crystallised with the downloaded protein since two hydrogen bonds were formed with amino acid residues Try151 and Ser95, also two hydrophobic bonding were observed through interaction with Leu120 and Tyr119 amino acids (Figure 9). However compound **10** lacks hydrogen bonding, it binds to the active protein with a binding affinity of −21.88 kcal/mol through hydrophobic interactions with Tyr59, Tyr119, and His15 residues (Figure 10).

**Figure 11.**
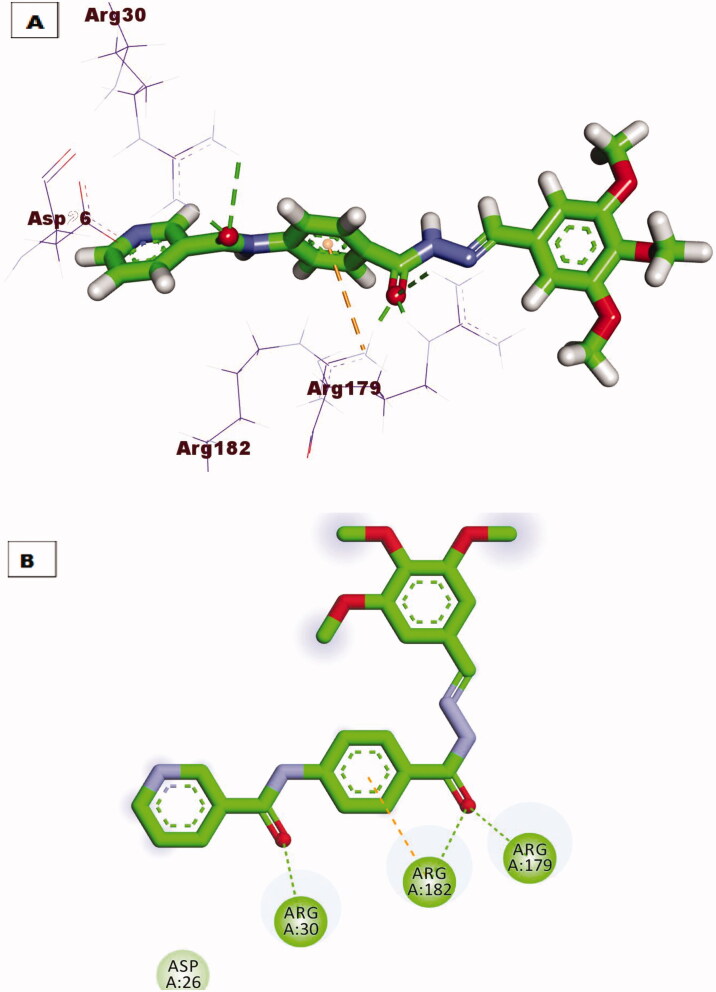
(A) 3D binding mode of compound **7** against IL-6 protein, (B) 2D binding mode of compound **7** against IL-6 protein.

**Figure 12.**
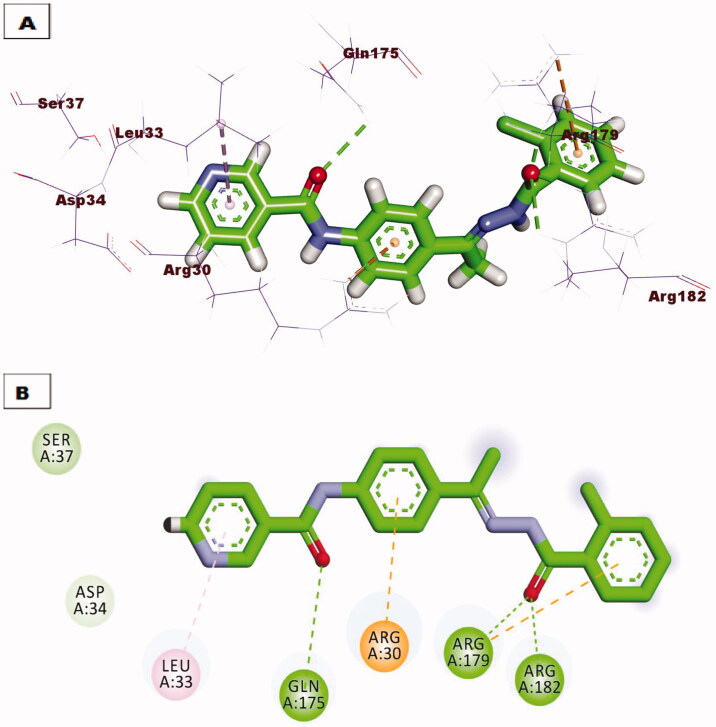
(A) 3D binding mode of compound **10** against IL-6 protein, (B) 2D binding mode of compound **10** against IL-6 protein.

Regarding docking against IL-6 protein, compound **7** demonstrated the same orientation and interactions of the tartaric acid co-crystallised with downloaded protein. Compound **7** exhibited an affinity value of −18. 87 kcal/mol through the formation of three hydrogen bonds with amino acid residues Arg82, Arg182, and Arg189, also extra hydrophobic interaction was formed with amino acid Arg182 (Figure 11). Compound **10** showed excellent binding mode against IL-6 protein, with a higher score function of −24.18 kcal/mol. Such compound was stabilised inside the active pocket by forming three hydrogen bonds with Arg182, Arg189, Gln175 and three hydrophobic interactions with Arg179, Arg30, and Leu33 (Figure 12).

#### 2.3.3. MD Simulations

##### 2.3.3.1. MD simulations against VEGFR-2

Investigating the Molecular dynamics (MD) simulations in drug-protein binding is closely a regular *in silico* experiment^35^. The MD study has two major points of interest. To begin with, the unequivocal capacity to describe any alter that happened within the ligand and the protein target either that alter was in the structure or an entropic. Also, the MD study computes the occurred changes for a decided time of an extraordinarily brief period and an exceptional resolution at the atomic level^36^. Hence, MD simulations can properly expect the changes that occurred in kinetics aspects and thermodynamics due to ligand-protein binding^37^. The outputs of such experiments are very useful to understand the ligand-target permanence, kinetics as well as energy^38^.

**Figure 13.**
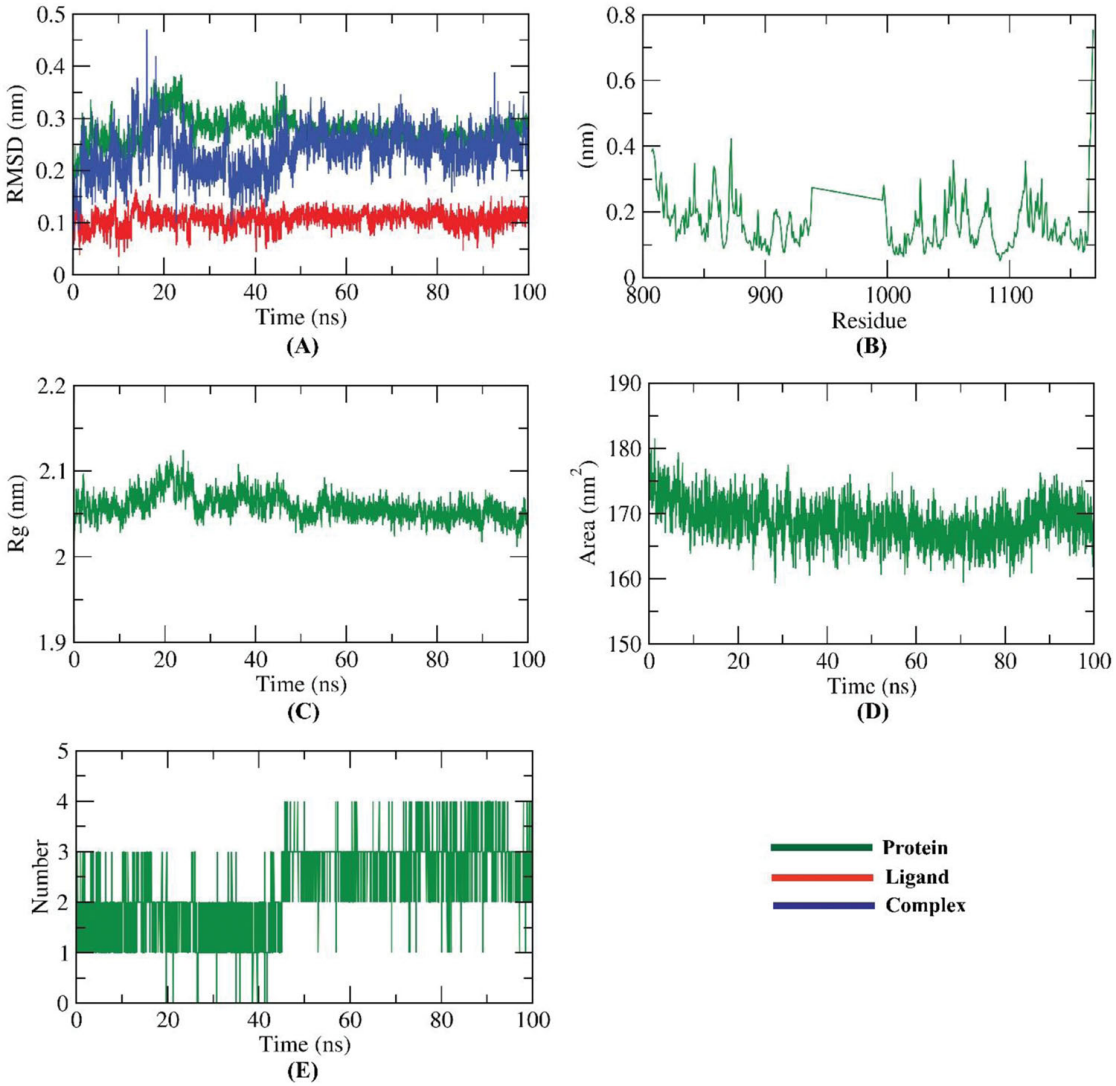
MD simulations experiment; (A) RMSD values of VEGFR-2 -compound **7** before and after binding, (B) RMSF of VEGFR-2 -compound **7** complex, (C) R_g_ of VEGFR-2 -compound **7** complex (D) SASA of VEGFR-2 -compound **7** complex, (E) H- bonding between VEGFR-2 -compound **7** complex.

To examine the stability of compound **7** inside the active site of VEGFR-2, the conformational changes of the VEGFR-2 -compound **7** complex were investigated over the course of 100 ns MD simulations utilising 5 different methods. Firstly, The RMSD value of the VEGFR-2 -compound **7** complex after binding with respect to the initial structure (Figure 13(A)) was estimated to demonstrate the changes that occurred in the dynamic and conformational aspects on an atomic level. The VEGFR-2, compound **7**, and VEGFR-2 -compound **7** complex demonstrated low RMSD values (less than 0.4) with very minute fluctuations from 15–25 ns followed by very stable behaviour till the end of the study. Secondly, to investigate the region of VEGFR-2 that have been fluctuated due to the binding with compound 7, the RMSF of each residue was computed. As shown in Figure 13(B), the binding of compound 7 didn’t cause a high degree of flexibility to VEGFR-2. Furthermore, the degree of VEGFR-2 -compound **7** complex compactness was revealed by the investigation of the radius of gyration (R_g_). Figure 13(C) demonstrates that the R_g_ values of the VEGFR-2 -compound **7** complex slightly fluctuated (2–2.1 nm) from starting till the end of the simulation time (100 ns). Such outputs indicate the compactness of the VEGFR-2 -compound **7** complex. Also, the interaction that occurred between the VEGFR-2 -compound **7** complex and solvents that are surrounding was revealed through the investigation of solvent accessible surface area (SASA) over the period of 100 ns. SASA values are a direct indication of the conformational dynamics that occurred due to the interaction. Figure 13(D) indicates the slight reduction of the SASA values of the VEGFR-2 than the starting time. Finally, hydrogen bonding within the VEGFR-2 -compound **7** complex was computed. The resulted outputs showed that the highest number of conformations of the VEGFR-2 formed up to four hydrogen bonds with compound **7** (Figure 13(E)).

##### 2.3.3.2. MM-PBSA studies against VEGFR-2

**Figure 14.**
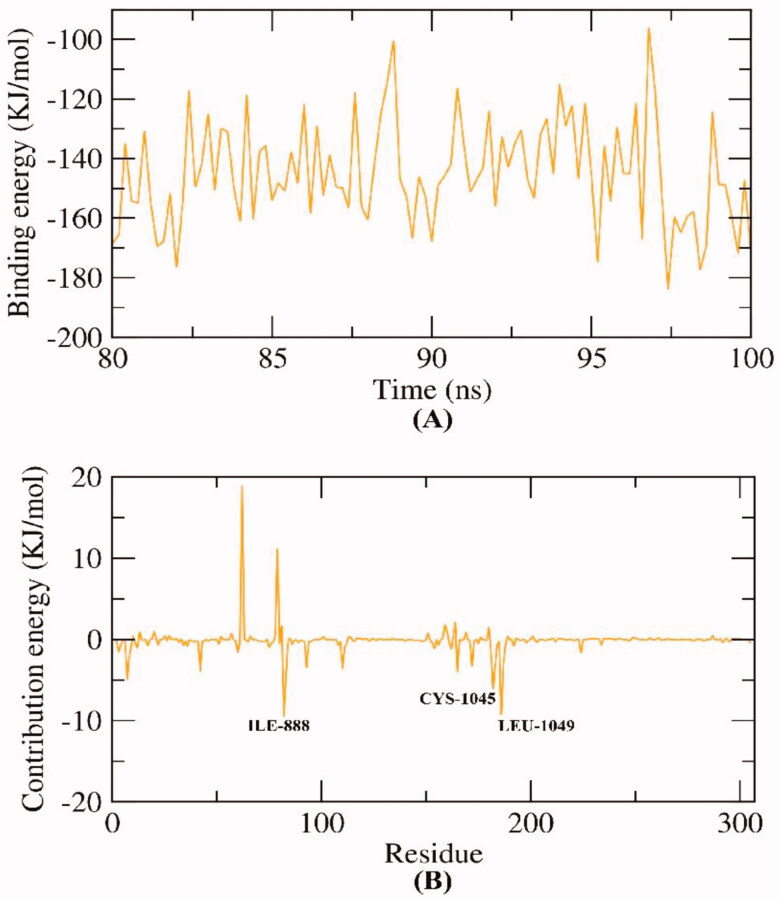
MM-PBSA results of VEGFR-2 -compound **7** complex.

We employed the molecular mechanics energies with the Poisson–Boltzmann Born and surface area (MM-PBSA) continuum solvation method to investigate the precise free energy of the VEGFR-2 -compound **7** complex because of the high accuracy of this approach^39^. The binding free energy of the VEGFR-2-compound **7** complex was computed from the resulted MD trajectories at the final 20 ns of the run having an interval of 100 ps. Additionally, the MmPbSaStat.py script was used to investigate the average free binding energy value as well as the standard deviation of that energy. As Figure 14(A) shows, compound **7** demonstrated a binding free energy of −145 KJ/mol with VEGFR-2.

Besides, the MM-PBSA was operated to distinguish the participation of every amino acid of VEGFR-2 in the total binding free energy that resulted from the interaction with compound **7**. The purpose of this study is to explore the pivotal amino acids that contributed advantageously to the binding of the VEGFR-2-compound **7** complex. As Figure 14(B) shows, ILE-888, CYS-1045, and LEU-1049 amino acids of VEGFR-2contributed more than −5 KJ/mol binding energy.

##### 2.3.3.3. MD simulations against IL-6

**Figure 15.**
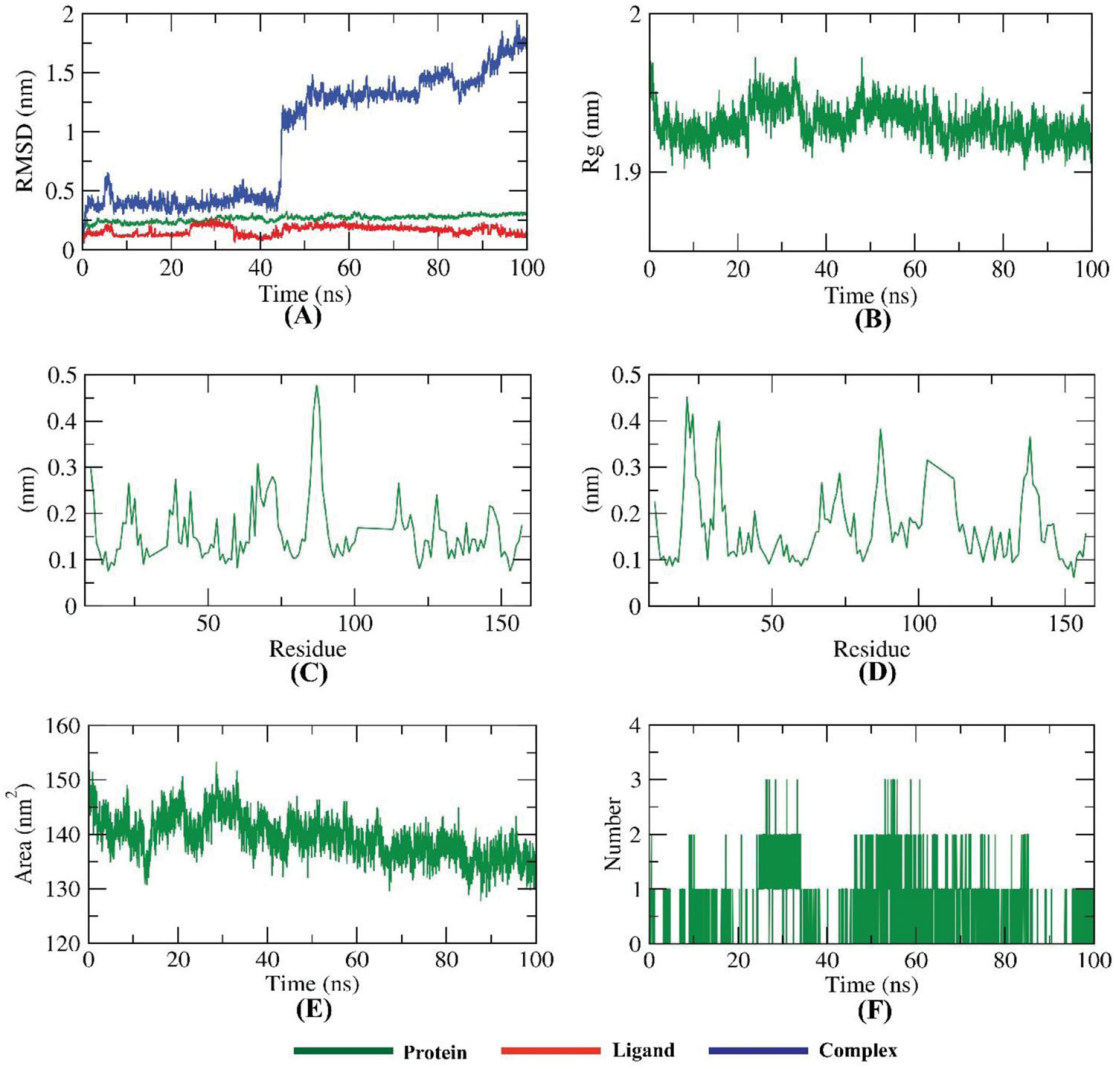
MD simulations experiment; (A) RMSD values of IL-6-compound **7** complex before and after binding, (B) R_g_ of IL-6-compound **7** complex, (C & D) RMSF of IL-6-compound **7** complex, (E) SASA of IL-6-compound **7** complex, (F) H- bonding between IL-6-compound **7** complex.

To examine the stability of compound **7** inside the active sites of IL-6, the conformational changes of the IL-6-compound **7** complex were investigated over the course of 100 ns MD simulations utilising 5 different methods. To demonstrate the changes that occurred in the dynamic and conformational aspects on an atomic level, the RMSD value of the IL-6-compound **7** complex after binding with respect to the initial structure (Figure 15(A)) was estimated. Both IL-6 and compound **7** demonstrated low RMSD values without fluctuations. However, the IL-6-compound **7** complex was stable till ∼42 ns and showed some minor fluctuations later. Further studies were conducted to confirm the stability of the IL-6-compound **7** complex. Additionally, Figure 15(B) demonstrates that the Rg values of the IL-6-compound **7** complex were more stable at the simulation time (100 ns) and found to be minimised at the end of the study than at the beginning. As shown in Figure 15(C,D), the binding of compound **7** didn’t cause a high degree of flexibility to IL-6. Figure 15(E) indicates the reduction of the SASA values of the IL-6 than the starting time verifying the stability of the IL-6-compound **7** complex. The resulted outputs showed that the highest number of conformations of the IL-6 formed up to three hydrogen bonds with compound **7** (Figure 15(F)).

##### 2.3.3.4. MD simulations against TNF-*α*

**Figure 16.**
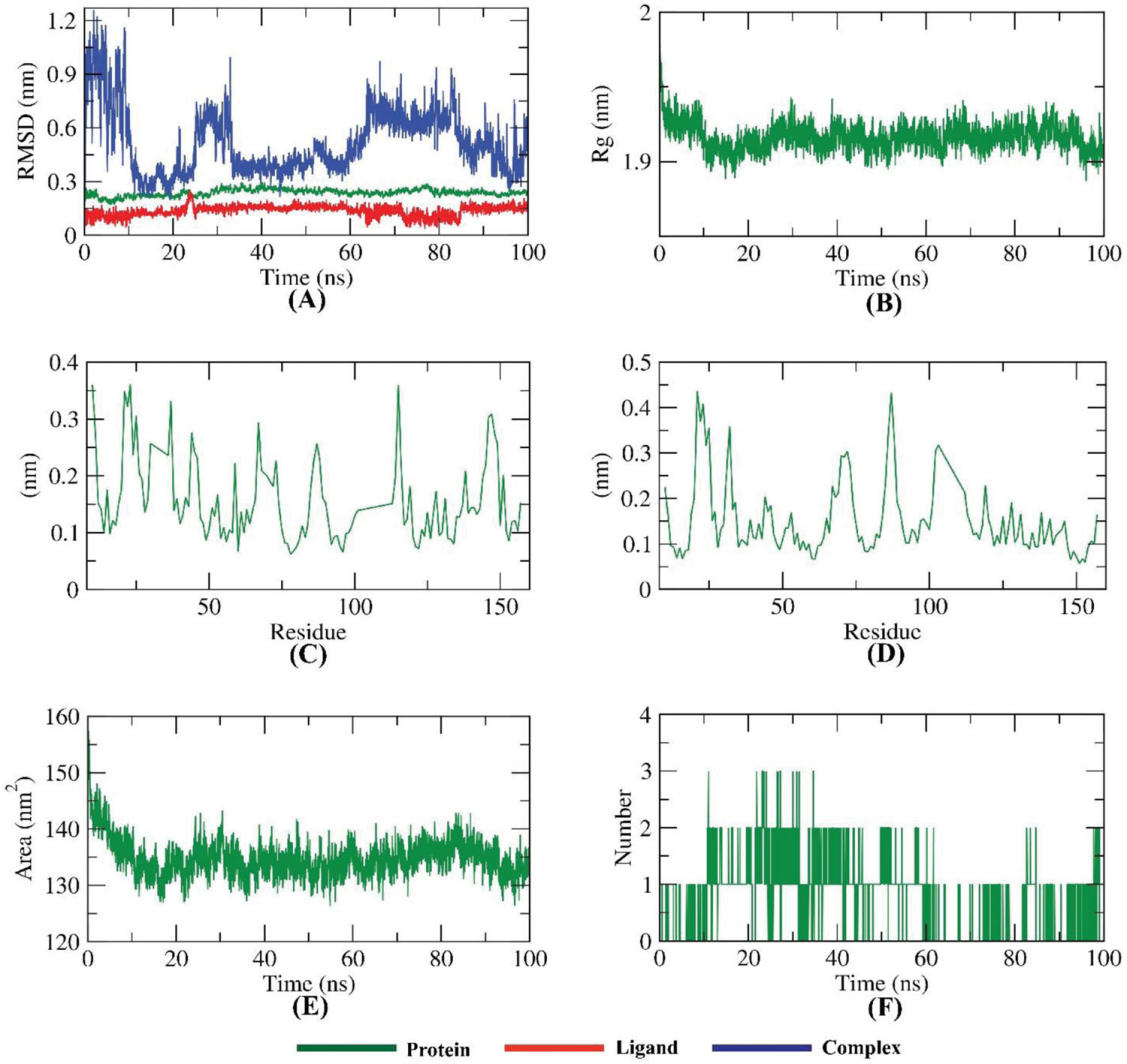
MD simulations experiment; (A) RMSD values of TNF-*α*-compound **7** complex before and after binding, (B) R_g_ of TNF-*α*-compound **7** complex, (C & D) RMSF of TNF-*α*-compound **7** complex, (E) SASA of TNF-*α*-compound **7** complex, (F) H- bonding between TNF-*α*-compound **7** complex.

To examine the stability of compound **7** inside the active sites of TNF-*α*, the conformational changes of the TNF-*α*-compound **7** complex were investigated over the course of 100 ns MD simulations utilising 5 different methods. To demonstrate the changes that occurred in the dynamic and conformational aspects on an atomic level, the RMSD value of the TNF-*α*-compound **7** complex after binding with respect to the initial structure (Figure 16(A)) was estimated. Both TNF-*α* and compound **7** demonstrated low RMSD values without fluctuations. However, the TNF-*α*-compound **7** complex fluctuated slightly throughout the simulation period. Further studies were conducted to confirm the stability of the TNF-*α*-compound **7** complex. Additionally, Figure 16(B). demonstrates that the Rg values of the TNF-*α*-compound **7** complex were more stable at the simulation time (100 ns) and found to be minimised at the end of the study than at the beginning. As shown in Figure 16(C,D), the binding of compound **7** didn’t cause a high degree of flexibility to TNF-*α*. Figure 16(E) indicates the reduction of the SASA values of the TNF-*α* than the starting time verifying the stability of the TNF*-α*-compound **7** complex. The resulted outputs showed that the highest number of conformations of the VEGFR-2 TNF-*α* formed up to three hydrogen bonds with compound **7** (Figure 16(F)).

#### 2.3.5. *In silico* ADMET analysis

**Figure 17.**
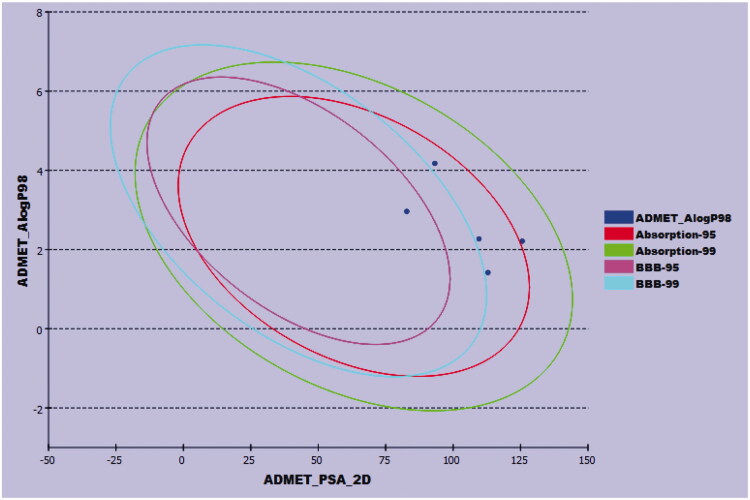
The predicted ADMET parameters.

The pharmacokinetic characters of the synthesised compounds were predicted using Discovery Studio 4.0 software in the presence of sorafenib as a reference. Except for compound **10**, which demonstrated a low amount of BBB penetration, all the tested compounds were predicted to have very low levels of BBB penetration. These data point to general safety in the CNS. Furthermore, compounds **6**, **7**, and **11** have high aqueous solubility, but compound **10** was expected to have a low value. Additionally, compounds **7, 10,** and **11** showed high levels of absorption, whereas compound **6** showed a moderate level. Furthermore, none of the investigated members were anticipated to be CYP2D6 inhibitors, indicating a low toxicity effect on the liver. Finally, all compounds except for **11** were predicted to bind plasma protein at a rate greater than 90 (Figure 17).

#### 2.3.6. Toxicity studies

The *in silico* toxicity profile was assessed utilising Discovery studio software 4.0 in the presence of sorafenib as a positive control. The results were depicted in Table 6. Firstly, the Ames mutagenicity prediction model was computed and predicted that all of the tested compounds were expected to be non-mutagen except compound **6**. Additionally, compounds **6, 7, 10,** and **11** showed anticipated carcinogenic potency (TD_50_) values of 28.360, 42.483, 26.437, and 28.065 g/kg, respectively in mice. These values are higher than that of sorafenib (19.236 g/kg). Further, compounds **6**, **10**, and **11** showed expected maximum tolerated dose (MTD) values of 0.094, 0.142, and 0.122 g/kg in rats which are higher than that of sorafenib (0.089 g/kg). on the other hand, compound **7** showed a maximum tolerated dose of 0.056 g/kg which is lower than that of sorafenib. Likewise, all compounds showed anticipated rat oral LD_50_ values ranging from 2.113 to 6.781 g/kg which are higher than that of sorafenib (0.823 g/kg). In addition, the predicted rat chronic LOAEL values of the tested compounds are ranging from 0.077 to 0.639 g/kg. These values are higher than sorafenib (0.005 g/kg). Finally, all compounds were expected to have mild irritant effects in the ocular irritancy model as well as to be non-irritant in the skin irritancy model.

**Table 6.** *In silico* toxicity potential of the synthesised compounds

Comp.	Ames mutagenicity prediction	TD_50_^a^	MTD^b^	LD_50_^b^	LOAEL^b^	Skin irritancy	Ocular irritancy
**6**	Mutagen	28.360	0.094	2.727	0.077	None irritant	Mild irritant
**7**	Non-Mutagen	42.483	0.056	2.113	0.091		
**10**		26.437	0.142	3.754	0.639		
**11**		28.065	0.122	6.781	0.315		
**Sorafenib**	Non-Mutagen	19.236	0.089	0.823	0.005	None	Mild

^a^Unit: g/kg.

^b^Unit: mg/kg body.

## 3. Conclusion

A new four nicotinamide-based derivatives were designed and synthesised to be anticancer candidates inhibiting VEGFR-2. The synthesised candidates displayed favourable antiproliferative effects against the human tumour cell lines (HCT-116 and HepG2), and promising VEGFR-2 inhibitory effects. Compounds **7** and **10** revealed cytotoxic activities against HCT-116 with IC_50_ values of 15.7 and 15.4 as well as against HepG2 cell lines with IC_50_ values of 15.5 and 9.8 µM, respectively compared to sorafenib (IC_50_ = 9.30 and 7.40 µM). Similarly, all compounds owned promising VEGFR-2 inhibitory activities with sub-micromolar IC_50_ values. Compound **7** -as a representative example- was subjected to further mechanistic studies. It induced the cell cycle cessation at the cycle at %G2-M and G0-G1phases and induced the apoptosis in the HCT-116 cells by 5-folds compared to control. Additionally, compounds **7** and **10** reduced the levels of the immunomodulatory proteins TNF-α by the percentages of 81.6 and 84.5 besides IL-6 by the percentages of 88.4 and 60.9, respectively, compared to dexamethasone (82.4 and 93.1). Further, the *in silico* docking, MD simulations, and MM-PBSA studies confirmed the correct affinity as well as the optimum dynamics of the compound **7** -VEGFR-2, compound **7** - IL-6, and compound **7** -TNF-*α* complexes. Finally, the *in silico* ADMET and toxicity analysis revealed the general drug-likeness and safety.

## 4. Experimental

### 4.1. Chemistry

#### 4.1.1. General

All reagents, chemicals, and apparatus were shown in the Supplementary data. Compounds **2, 3, 4, 5,** and **9** were previously reported^23^^,^^40^.

#### 4.1.2. General procedure for the synthesis of compounds 6 and 7

A mixture of hydrazide derivative **5** (0.256 g, 0.001 mol) and the appropriate aromatic aldehyde (0.001 mol) namely, 3-nitrobenzaldehyde, and 3,4,5-trimethoxy benzaldehyde (0.001 mol) was refluxed in absolute ethanol (30 ml) containing few drops of glacial acetic acid for 2 h. After reaction completion, the mixture was cooled to room temperature then, the formed precipitate was filtered, dried, and recrystallized from ethanol to afford compounds **6**, and **7**, respectively.

##### 4.1.2.1. (E)-N-(4–(2-(3-Nitrobenzylidene)hydrazine-1-carbonyl)phenyl)nicotinamide (6)

White crystal (yield, 76%); m. p. = 278–280 °C; C_20_H_15_N_5_O_4_ (389.37); IR (KBr) *ν* cm^−1^: 3247, 3174 (NH), 3083 (CH aromatic), 1650 (C=O), 1597 (C=N); ^1^H NMR (400 MHz, DMSO-*d*_6_) δ 12.08 (s, 1H), 10.72 (s, 1H), 9.18 − 9.09 (m, 1H), 8.84 − 8.77 (m, 1H), 8.57 (d, *J* = 9.0 Hz, 2H), 8.36 − 8.24 (m, 2H), 8.17 (d, *J* = 7.8 Hz, 1H), 7.97 (t, *J* = 7.3 Hz, 4H), 7.77 (t, *J* = 8.0 Hz, 1H), 7.60 (ddt, *J* = 7.9, 4.8, 0.8 Hz, 1H); ^13 ^C NMR (101 MHz, DMSO-*d*_6_) δ 164.91(2 C), 163.21(2 C), 148.72(4 C), 142.67(4 C), 136.76(2 C), 130.80(2 C), 128.54(4 C).

##### 4.1.2.2. (E)-N-(4–(2-(3,4,5-Trimethoxybenzylidene)hydrazine-1-carbonyl)phenyl) nicotinamide (7)

Off-white crystal (yield, 84%); m. p. = 257–259 °C; C_23_H_22_N_4_O5 (434.45); IR (KBr) *ν* cm^−1^: 3290, 3224 (NH), 3039 (CH aromatic), 2999 (CH aliphatic), 1650 (C=O), 1584 (C=N); ^1^H NMR (400 MHz, DMSO-*d*_6_) δ 11.86 − 11.76 (m, 1H), 10.71 (s, 1H), 9.15 (dd, *J* = 2.4, 0.9 Hz, 1H), 8.79 (dd, *J* = 4.8, 1.7 Hz, 1H), 8.51 − 8.23 (m, 2H), 8.14 − 7.76 (m, 4H), 7.60 (ddd, *J* = 8.0, 4.8, 0.9 Hz, 1H), 7.04 (s, 2H), 3.79 (d, *J* = 52.8 Hz, 9H); ^13 ^C NMR (101 MHz, DMSO-*d*6) δ 164.89(2 C), 163.02, 153.68(3 C), 142.45(2 C), 139.67(4 C), 130.81(4 C), 130.40(4 C), 60.30, 56.38(2 C).

#### 4.1.3. General procedure for the synthesis of compounds 10 and 11

To a solution of compound **9** (0.24 g, 0.001 mol) in absolute ethanol (20 ml) a catalytic amount of glacial acetic acid (3 drops), the appropriate amine derivatives namely, 2-chlorobenzohydrazide and *N-*(4-(hydrazinecarbonyl)phenyl)acetamide (10 mmol) were added. The reaction mixture was heated under reflux for 8 h. The resulting solids were filtered, washed with water, dried, and crystallised from ethanol to afford the target compounds **10** and **11,** respectively.

##### 4.1.3.1. ((E)-N-(4–(1-(2–(2-Chlorobenzoyl)hydrazono)ethyl)phenyl)nicotinamide (10)

Brown crystal **(**yield, 75%); m. p. = 240–241 °C; C_21_H_17_ClN_4_O_2_ (392.84); IR (KBr) *ν* cm^−1^: 3448, 3337 (NH), 3172 (CH aromatic), 2970 (CH aliphatic), 1669 (C=O), 1597 (C=N); ^1^H NMR (400 MHz, DMSO-*d*_6_) δ 10.97 (s, 1H), 10.61 (s, 1H), 9.11 (ddd, *J* = 22.5, 2.4, 0.9 Hz, 1H), 8.77 (ddd, *J* = 8.1, 4.8, 1.7 Hz, 1H), 8.35 − 8.22 (m, 1H), 7.88 (s, 2H), 7.72 − 7.65 (m, 1H), 7.60 − 7.42 (m, 6H), 2.30 (d, *J* = 13.9 Hz, 3H); ^13 ^C NMR (101 MHz, DMSO-*d*_6_) δ 170.03, 164.67, 164.58, 163.42, 154.69, 148.50, 140.59, 140.05, 137.24, 136.26(2 C), 133.91(2 C), 133.81(2 C), 130.95, 130.92, 130.20, 127.57, 120.23, 14.92.

##### 4.1.3.2. (E)-N-(4–(1-(2–(4-Acetamidobenzoyl)hydrazono)ethyl)phenyl)nicotinamide (11)

Yellow crystal **(**yield, 72%); m. p. = 285–287 °C; C_23_H_21_N_5_O_3_ (415.45); IR (KBr) *ν* cm^−1^: 3315, 3236 (NH), 3098 (CH aromatic), 1647 (C=O), 1602 (C=N); 1H NMR (400 MHz, DMSO-*d*6) δ 11.22 (s, 1H), 10.61 (s, 1H), 10.49 (s, 1H), 9.11 (ddd, *J* = 22.5, 2.4, 0.9 Hz, 1H), 8.77 (ddd, *J* = 8.1, 4.8, 1.7 Hz, 1H), 8.34 − 8.25 (m, 1H), 7.88 (s, 2H), 7.70 − 7.65 (m, 1H), 7.59 − 7.43 (m, 6H), 3.46 − 3.36 (m, 3H), 2.30 (d, *J* = 13.9 Hz, 3H); ^13 ^C NMR (101 MHz, DMSO-*d*_6_) δ 170.03, 164.67, 164.58, 163.42, 154.69, 148.50, 140.59, 140.05, 137.24, 136.26(2 C), 133.91(2 C), 133.81(2 C), 130.95(2 C), 130.92, 130.20, 127.57, 120.23, 14.84, 13.93.

### 4.2. Biological testing

#### 4.2.1. *In vitro* antiproliferative activity

MTT procedure^41^^,^^42^ was applied for the determination of the anti-proliferative activity as described in Supplementary data.

#### 4.2.2. *In vitro* VEGFR-2 enzyme inhibition assay

VEGFR-2 inhibitory activity was assessed using Human VEGFR-2 ELISA kit^43^ as described in Supplementary data.

#### 4.2.3. Flow cytometry analysis for cell cycle

Cell cycle analysis was performed using propidium iodide (PI) staining and flow cytometry analysis for compound **7** as described in Supplementary data^44^^,^^45^.

#### 4.2.4. Flow cytometry analysis for apoptosis

Flow cytometry cell apoptosis analysis was used to investigate the apoptotic effect of compound **7** as described in Supplementary data^46^^,^^47^.

#### 4.2.5. Quantitative Real-Time Reverse-Transcriptase PCR (qRT-PCR) technique

The effect of compounds **7** and **10** on the expression of TNF-α and IL-6 were determined using qRT-PCR as described in Supplementary data^48–50^.

### 4.3. *In silico* studies

#### 4.3.1. Docking studies

MOE 2014 software was used for carrying out the docking studies against VEGFR-2 (PDB: 4ASD), TNF-α (PDB ID: 2AZ5), and IL-6 (PDB ID: 1ALU) as described in Supplementary data^51^.

#### 4.3.2. ADMET studies

ADMET descriptors were determined using Discovery studio 4.0^52^ according to the reported method (Supplementary data).

#### 4.3.3. Toxicity studies

The toxicity parameters of the synthesised compounds were calculated using Discovery studio 4.0 as described in Supplementary data^53^.

#### 4.3.4. Molecular dynamics simulation & MM/PBSA

MD simulation experiments and MM/PBSA (Molecular Mechanics/Poisson Boltzmann Surface Area) were carried out against VEGFR-2, TNF-α, and IL-6 using GROMACS as reported in Supplementary data^54^.

## Supplementary Material

Supplemental Material

## References

[1] Siegel RL, Miller KD, Jemal A. Cancer statistics, 2020. CA Cancer J Clin 2020;70:7–30. 31912902 10.3322/caac.21590

[2] Fabbro D, Parkinson D, Matter A. Protein tyrosine kinase inhibitors: new treatment modalities? Curr Opin Pharmacol 2002;2:374–81. 12127869 10.1016/s1471-4892(02)00179-0

[3] Abdallah AE, Mabrouk RR, Al Ward MMS, et al. Synthesis, biological evaluation, and molecular docking of new series of antitumor and apoptosis inducers designed as VEGFR-2 inhibitors. J Enzyme Inhib Med Chem 2022;37:573–91. 35012403 10.1080/14756366.2021.2017911 PMC8757611

[4] Phillips CM, Lima EA, Woodall RT, et al. A hybrid model of tumor growth and angiogenesis: *in silico* experiments. PLoS One 2020;15:e0231137. 32275674 10.1371/journal.pone.0231137 PMC7147760

[5] Patel HM, Bari P, Karpoormath R, et al. Design and synthesis of VEGFR-2 tyrosine kinase inhibitors as potential anticancer agents by virtual based screening. RSC Adv 2015;5:56724–71. 10.1039/d5ra90070k PMC12135165 40476244

[6] Shibuya M. Vascular endothelial growth factor and its receptor system: physiological functions in angiogenesis and pathological roles in various diseases. J Biochem 2013;153:13–9. 23172303 10.1093/jb/mvs136 PMC3528006

[7] Frezzetti D, Gallo M, Roma C, et al. Vascular endothelial growth factor a regulates the secretion of different angiogenic factors in lung cancer cells. J Cell Physiol 2016;231:1514–21. 26542886 10.1002/jcp.25243

[8] Stuttfeld E, Ballmer‐Hofer K. Structure and function of VEGF receptors. IUBMB Life 2009;61:915–22. 19658168 10.1002/iub.234

[9] El-Metwally SA, Abou-El-Regal MM, Eissa IH, et al. Discovery of thieno [2, 3-d] pyrimidine-based derivatives as potent VEGFR-2 kinase inhibitors and anti-cancer agents. Bioorg Chem 2021;112:104947. 33964580 10.1016/j.bioorg.2021.104947

[10] Shahin MI, Abou El Ella DA, Ismail NS, Abouzid KA. Design, synthesis and biological evaluation of type-II VEGFR-2 inhibitors based on quinoxaline scaffold. Bioorg Chem 2014;56:16–26. 24922538 10.1016/j.bioorg.2014.05.010

[11] Yousef RG, Ibrahim A, Khalifa MM, et al. Discovery of new nicotinamides as apoptotic VEGFR-2 inhibitors: virtual screening, synthesis, anti-proliferative, immunomodulatory, ADMET, toxicity, and molecular dynamic simulation studies. J Enzyme Inhib Med Chem 2022;37:1389–403. 35577416 10.1080/14756366.2022.2070744 PMC9116259

[12] Elrazaz EZ, Serya RA, Ismail NS, et al. Discovery of potent thieno [2, 3-d] pyrimidine VEGFR-2 inhibitors: design, synthesis and enzyme inhibitory evaluation supported by molecular dynamics simulations. Bioorg Chem 2021;113:105019. 34091286 10.1016/j.bioorg.2021.105019

[13] Lee K, Jeong K-W, Lee Y, et al. Pharmacophore modeling and virtual screening studies for new VEGFR-2 kinase inhibitors. Eur J Med Chem 2010;45:5420–7. 20869793 10.1016/j.ejmech.2010.09.002

[14] Machado VA, Peixoto D, Costa R, et al. Synthesis, antiangiogenesis evaluation and molecular docking studies of 1-aryl-3-[(thieno [3, 2-b] pyridin-7-ylthio) phenyl] ureas: discovery of a new substitution pattern for type II VEGFR-2 Tyr kinase inhibitors. Bioorg Med Chem 2015;23:6497–509. 26344591 10.1016/j.bmc.2015.08.010

[15] Garofalo A, Goossens L, Six P, et al. Impact of aryloxy-linked quinazolines: a novel series of selective VEGFR-2 receptor tyrosine kinase inhibitors. Bioorg Med Chem Lett 2011;21:2106–12. 21353546 10.1016/j.bmcl.2011.01.137

[16] Dunlop RJ, Campbell CW. Cytokines and advanced cancer. J Pain Symptom Manage 2000;20:214–32. 11018340 10.1016/s0885-3924(00)00199-8

[17] Michalaki V, Syrigos K, Charles P, Waxman J. Serum levels of IL-6 and TNF-α correlate with clinicopathological features and patient survival in patients with prostate cancer. Br J Cancer 2004;90:2312–6. 15150588 10.1038/sj.bjc.6601814 PMC2409519

[18] Taher MY, Davies DM, Maher J. The role of the interleukin (IL)-6/IL-6 receptor axis in cancer. Biochem Soc Trans 2018;46:1449–62. 30467123 10.1042/BST20180136

[19] Balkwill FJC. TNF-alpha in promotion and progression of cancer. Cancer Metastasis Rev 2006;25:409–16. 16951987 10.1007/s10555-006-9005-3

[20] Ungerstedt JS, Blombäck M, Söderström T. Nicotinamide is a potent inhibitor of proinflammatory cytokines. Clin Exp Immunol 2003;131:48–52. 12519385 10.1046/j.1365-2249.2003.02031.x PMC1808598

[21] Zeidan MA, Mostafa AS, Gomaa RM, et al. Design, synthesis and docking study of novel picolinamide derivatives as anticancer agents and VEGFR-2 inhibitors. Eur J Med Chem 2019;168:315–29. 30826508 10.1016/j.ejmech.2019.02.050

[22] Hagras M, El Deeb MA, Elzahabi HS, et al. Discovery of new quinolines as potent colchicine binding site inhibitors: design, synthesis, docking studies, and anti-proliferative evaluation. J Enzyme Inhib Med Chem 2021;36:640–58. 33588683 10.1080/14756366.2021.1883598 PMC7889231

[23] Ran F, Li W, Qin Y, et al. Inhibition of vascular smooth muscle and cancer cell proliferation by new VEGFR inhibitors and their immunomodulator effect: design, synthesis, and biological evaluation. Oxid Med Cell Longev 2021;2021:1–21. 10.1155/2021/8321400 PMC8568530 34745424

[24] Zhang Q, Li J, Zhong H, Xu Y. The mechanism of nicotinamide on reducing acute lung injury by inhibiting MAPK and NF-κB signal pathway. Mol Med 2021;27:1–11. 34544355 10.1186/s10020-021-00376-2 PMC8451170

[25] Grange PA, Raingeaud J, Calvez V, Dupin N. Nicotinamide inhibits propionibacterium acnes-induced IL-8 production in keratinocytes through the NF-κB and MAPK pathways. J Dermatol Sci 2009;56:106–12. 19726162 10.1016/j.jdermsci.2009.08.001

[26] He MM, Smith AS, Oslob JD, et al. Small-molecule inhibition ofTNF-a. Science 2005;310:1022–5. 16284179 10.1126/science.1116304

[27] Zia K, Ashraf S, Jabeen A, et al. Identification of potential TNF-α inhibitors: from *in silico* to *in vitro* studies. Sci Rep 2020;10:1–9. 33262408 10.1038/s41598-020-77750-3 PMC7708426

[28] Alanazi MM, Eissa IH, Alsaif NA, et al. Design, synthesis, docking, ADMET studies, and anticancer evaluation of new 3-methylquinoxaline derivatives as VEGFR-2 inhibitors and apoptosis inducers. J Enzyme Inhib Med Chem 2021;36:1760–82. 34340610 10.1080/14756366.2021.1956488 PMC8344243

[29] El-Adl K, El-Helby A-GA, Sakr H, Elwan A. [1, 2, 4] Triazolo [4, 3-a] quinoxaline and [1, 2, 4] triazolo [4, 3-a] quinoxaline-1-thiol-derived DNA intercalators: design, synthesis, molecular docking, *in silico* ADMET profiles and anti-proliferative evaluations. N J Chem 2021;45:881–97. 10.1016/j.bioorg.2020.104399 33113414

[30] El-Adl K, El-Helby A-GA, Sakr H, Elwan A. Design, synthesis, molecular docking and anti-proliferative evaluations of [1, 2, 4] triazolo [4, 3-a]. Quinoxaline Derivatives as DNA Intercalators and Topoisomerase II Inhibitors. Bioorg Chem 2020;105:104399. 33113414 10.1016/j.bioorg.2020.104399

[31] Chan KT, Meng FY, Li Q, et al. Cucurbitacin B induces apoptosis and S phase cell cycle arrest in BEL-7402 human hepatocellular carcinoma cells and is effective via oral administration. Cancer Letters 2010;294:118–24. 20153103 10.1016/j.canlet.2010.01.029

[32] Sobhy MK, Mowafy S, Lasheen DS, et al. 3D-QSAR pharmacophore modelling, virtual screening and docking studies for lead discovery of a novel scaffold for VEGFR 2 inhibitors: design, synthesis and biological evaluation. Bioorg Chem 2019;89:102988. 31146197 10.1016/j.bioorg.2019.102988

[33] Mena-Ulecia K, Tiznado W, Caballero J. Study of the differential activity of thrombin inhibitors using docking, QSAR, molecular dynamics, and MM-GBSA. PLoS One 2015;10:e0142774. 26599107 10.1371/journal.pone.0142774 PMC4657979

[34] Wang S-Q, Shi M, Fang L, et al. Design of dual inhibitors of human TNF-α and IL-6 with potentials for the treatment of rheumatoid arthritis. Trop J Pharm Res 2019;18:2305–12.

[35] Sousa SF, Fernandes PA, Ramos MJ. Protein–ligand docking: current status and future challenges. Proteins: Struct Funct Bioinformatics 2006;65:15–26. 10.1002/prot.21082 16862531

[36] Hollingsworth SA, Dror RO. Molecular dynamics simulation for all. Neuron 2018;99:1129–43. 30236283 10.1016/j.neuron.2018.08.011 PMC6209097

[37] Hansson T, Oostenbrink C, van Gunsteren W. Molecular dynamics simulations. Curr Opin Struct Biol 2002;12:190–6. 11959496 10.1016/s0959-440x(02)00308-1

[38] Durrant JD, McCammon JA. Molecular dynamics simulations and drug discovery. BMC Biol 2011;9:1–9. 22035460 10.1186/1741-7007-9-71 PMC3203851

[39] Genheden S, Ryde U. The MM/PBSA and MM/GBSA methods to estimate ligand-binding affinities. Expert Opin Drug Discov 2015;10:449–61. 25835573 10.1517/17460441.2015.1032936 PMC4487606

[40] Chaitra G, Rohini R. Synthesis and biological activities of [1, 3]‐oxazine derivatives. Der Pharma Chem 2018;10:96–101.

[41] Eldehna WM, Al-Rashood ST, Al-Warhi T, et al. Novel oxindole/benzofuran hybrids as potential dual CDK2/GSK-3β inhibitors targeting breast cancer: design, synthesis, biological evaluation, and *in silico* studies. J Enzyme Inhib Med Chem 2021;36:270–85. 33327806 10.1080/14756366.2020.1862101 PMC7751407

[42] Al-Sanea MM, Al-Ansary GH, Elsayed ZM, et al. Development of 3-methyl/3-(morpholinomethyl) benzofuran derivatives as novel antitumor agents towards non-small cell lung cancer cells. J Enzyme Inhib Med Chem 2021;36:987–99. 33985397 10.1080/14756366.2021.1915302 PMC8128204

[43] Abou-Seri SM, Eldehna WM, Ali MM, Abou El Ella DA. 1-Piperazinylphthalazines as potential VEGFR-2 inhibitors and anticancer agents: synthesis and *in vitro* biological evaluation. Eur J Med Chem 2016;107:165–79. 26590508 10.1016/j.ejmech.2015.10.053

[44] Wang J, Lenardo MJ. Roles of caspases in apoptosis, development, and cytokine maturation revealed by homozygous gene deficiencies. J Cell Sci 2000;113:753–7. 10671365 10.1242/jcs.113.5.753

[45] Eldehna WM, Hassan GS, Al-Rashood ST, et al. Synthesis and *in vitro* anticancer activity of certain novel 1-(2-methyl-6-arylpyridin-3-yl)-3-phenylureas as apoptosis-inducing agents. J Enzyme Inhib Med Chem 2019;34:322–32. 30722708 10.1080/14756366.2018.1547286 PMC6366416

[46] Lo KK-W, Lee TK-M, Lau JS-Y, et al. Luminescent biological probes derived from ruthenium (II) estradiol polypyridine complexes. Inorg Chem 2008;47:200–8. 18067284 10.1021/ic701735q

[47] Sabt A, Abdelhafez OM, El-Haggar RS, et al. Novel coumarin-6-sulfonamides as apoptotic anti-proliferative agents: synthesis, *in vitro* biological evaluation, and QSAR studies. J Enzyme Inhib Med Chem 2018;33:1095–107. 29944015 10.1080/14756366.2018.1477137 PMC6022226

[48] Balah A, Ezzat O, Akool E-S. Vitamin E inhibits cyclosporin A-induced CTGF and TIMP-1 expression by repressing ROS-mediated activation of TGF-β/Smad signaling pathway in rat liver. Int Immunopharmacol 2018;65:493–502. 30391882 10.1016/j.intimp.2018.09.033

[49] Aborehab NM, Elnagar MR, Waly NE. Gallic acid potentiates the apoptotic effect of paclitaxel and carboplatin via overexpression of Bax and P53 on the MCF‐7 human breast cancer cell line. J Biochem Mol Toxicol 2021;35:e22638. 33002289 10.1002/jbt.22638

[50] Elnagar MR, Walls AB, Helal GK, et al. Functional characterization of α7 nicotinic acetylcholine and NMDA receptor signaling in SH-SY5Y neuroblastoma cells in an ERK phosphorylation assay. Eur J Pharmacol 2018;826:106–13. 29501870 10.1016/j.ejphar.2018.02.047

[51] Ibrahim MK, Eissa IH, Alesawy MS, et al. Design, synthesis, molecular modeling and anti-hyperglycemic evaluation of quinazolin-4 (3H)-one derivatives as potential PPARγ and SUR agonists. Bioorg Med Chem 2017;25:4723–44. 28720328 10.1016/j.bmc.2017.07.015

[52] Suleimen YM, Jose RA, Suleimen RN, et al. Jusanin, a new flavonoid from artemisia commutata with an *in silico* inhibitory potential against the SARS-CoV-2 main protease. Molecules 2022;27:1636. 35268738 10.3390/molecules27051636 PMC8911936

[53] Alesawy MS, Elkaeed EB, Alsfouk AA, et al. *In silico* screening of semi-synthesized compounds as potential inhibitors for SARS-CoV-2 papain-like protease: pharmacophoric features, molecular docking, ADMET, toxicity and DFT studies. Molecules 2021;26:6593. 34771004 10.3390/molecules26216593 PMC8588135

[54] Suleimen YM, Jose RA, Suleimen RN, et al. Isolation and *In Silico* Anti-SARS-CoV-2 papain-like protease potentialities of two rare 2-phenoxychromone derivatives from Artemisia spp. Molecules 2022;27:1216. 35209006 10.3390/molecules27041216 PMC8879996

