# Supplementary material for: Anti-cancer and immunomodulatory evaluation of new nicotinamide derivatives as potential VEGFR-2 inhibitors and apoptosis inducers: in vitro and in silico studies
Source: J Enzyme Inhib Med Chem. 2022 Aug 18;37(1):2206–22. doi: 10.1080/14756366.2022.2110868 (PMC9466619; doi:10.1080/14756366.2022.2110868)
Supplement: Supplemental Material [file IENZ_A_2110868_SM6183.pdf]

Anti-cancer and immunomodulatory evaluation of new nicotinamide derivatives as potential VEGFR-2 inhibitors and apoptosis inducers: *in vitro* and *in silico* studies

Reda G.Yousef[a], Alaa Elwan[a], Ibraheem M. M. Gobaara[b], Ahmed B. M. Mehany[b], Wagdy M. Eldehna[c,d], Souad A. El-Metwally[e], Bshra A. Alsfouk[f], Eslam B. Elkaeed[g], Ahmed M. Metwaly[h,i]* Ibrahim. H. Eissa[a]*

[a] Pharmaceutical Medicinal Chemistry & Drug Design Department, Faculty of Pharmacy (Boys), Al-Azhar University, Cairo11884, Egypt.

[b] Zoology Department, Faculty of Science (Boys), Al-Azhar University, Cairo11884, Egypt.

[c] School of Biotechnology, Badr University in Cairo, Badr City, Cairo 11829, Egypt

[d] Department of Pharmaceutical Chemistry, Faculty of Pharmacy, Kafrelsheikh University, P.O. Box 33516, Kafrelsheikh, Egypt.

[e] Department of Basic Science, Higher Technological institute, 10th of Ramadan City, Egypt.

[f] Department of Pharmaceutical Sciences, College of Pharmacy, Princess Nourah bint Abdulrahman University, P.O. Box 84428, Riyadh 11671, Saudi Arabia

[g] Department of Pharmaceutical Sciences, College of Pharmacy, AlMaarefa University, Riyadh 13713, Saudi Arabia.

[h] Pharmacognosy and Medicinal Plants Department, Faculty of Pharmacy (Boys), Al-Azhar University, Cairo 11884, Egypt.

[i] Biopharmaceutical Products Research Department, Genetic Engineering and Biotechnology Research Institute, City of Scientific Research and Technological Applications (SRTA-City), Alexandria, Egypt.

***Corresponding authors:**

Ibrahim H. Eissa

Medicinal Chemistry Department, Faculty of Pharmacy (Boys), Al-Azhar University, Cairo 11884, Egypt. **Email:** Ibrahimeissa@azhar.edu.eg

Ahmed M. Metwaly

Pharmacognosy and Medicinal Plants Department, Faculty of Pharmacy (Boys), Al-Azhar University, Cairo 11884, Egypt. **Email:** ametwaly@azhar.edu.eg

Content

1	**4.1.1. Chemistry and materials**
2	**Spectral data of final target compounds 6, 7, 10** and **11**
3	**4.2. Experimental of Biological testing**<br>**4.2.1.** In vitro anti-proliferative activity<br>**4.2.2.** In vitro VEGFR-2 kinase assay<br>**4.2.3.** Flow cytometry analysis for cell cycle<br>**4.2.4.** Flow cytometry analysis for apoptosis<br>**4.2.5.** Quantitative Real Time Reverse-Transcriptase PCR technique
4	**4.3. Experimental of *in silico* studies**<br>**4.3.1.** Docking studies<br>• **Figure 7** ; Validation figure<br>• **Figure 8** ; Binding mode of Sorafenib<br>**4.3.2**. ADMET studies<br>**4.3.3.** Toxicity studies<br>**4.3.4.** Molecular dynamics simulation & MM/PBSA
5	**In silico toxicity data of final target compounds 10a-o**

1- **4.1.1. Chemistry and material**

All melting points were carried out by open capillary method on a Gallen kamp Melting point apparatus. The infrared spectra were recorded on pye Unicam SP 1000 IR spectrophotometer using potassium bromide disc technique. Proton magnetic resonance 1HNMR spectra were recorded on a Bruker 400 Megahertz-nuclear magnetic resonance (400 MHZ-NMR) spectrophotometer. Carbon-13 (C13) nuclear magnetic resonance (13CNMR) spectra were recorded on a Bruker 100 Megahertz-nuclear magnetic resonance (100 MHZ-NMR) spectrophotometer. Tetramethylsilane (TMS) was used as internal standard and chemical shifts were measured in δ scale one part per million (ppm). All compounds were within ±0.4 of the theoretical values. The reactions were monitored by thin-layer chromatography (TLC) using TLC sheets precoated with UV fluorescent silica gel Merck 60 F254 plates and were visualized using ultraviolet (UV) lamp and different solvents as mobile phases.

2- **Spectral data of final target compounds 6,7,10 and 11**

IR of compound 6

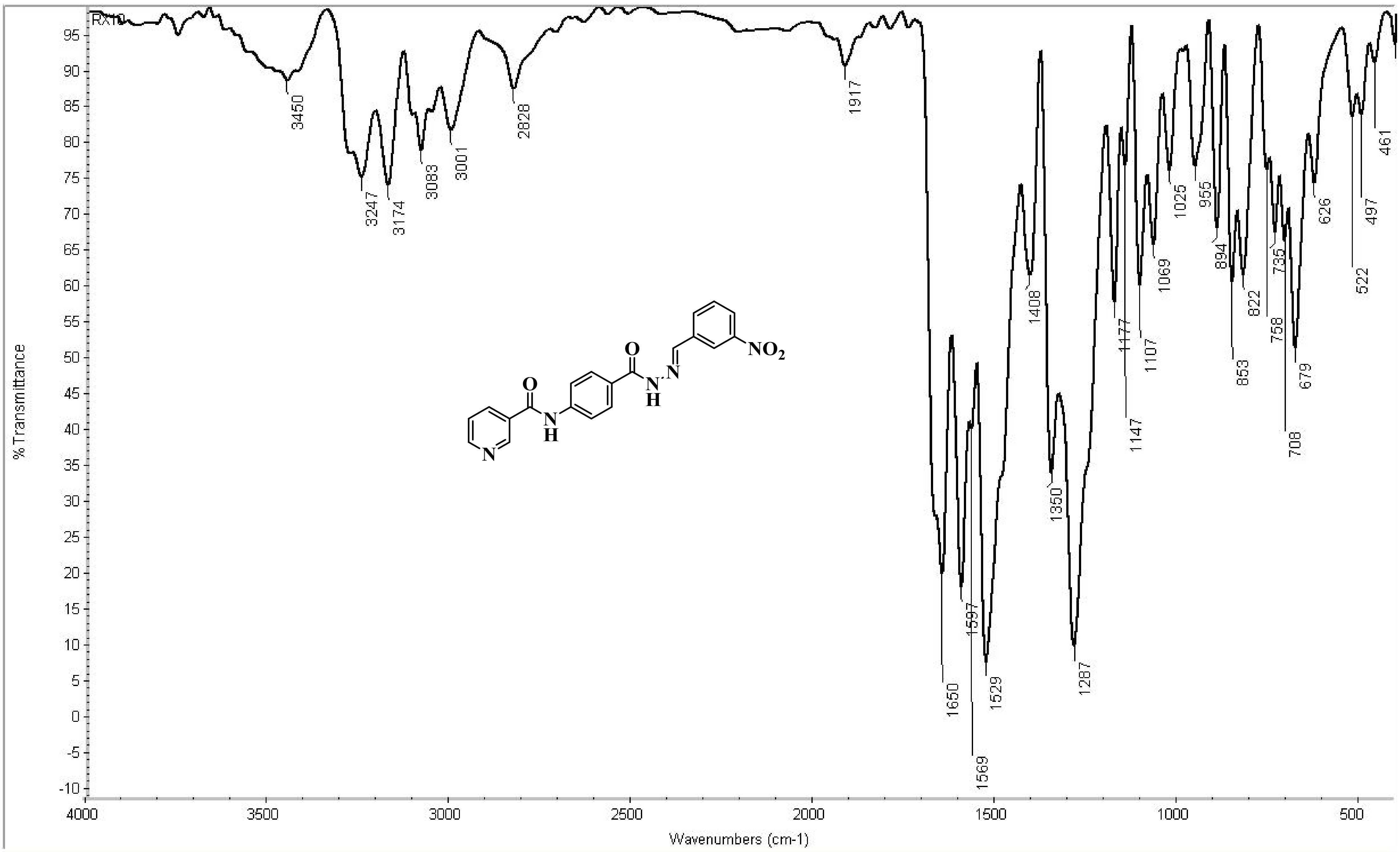

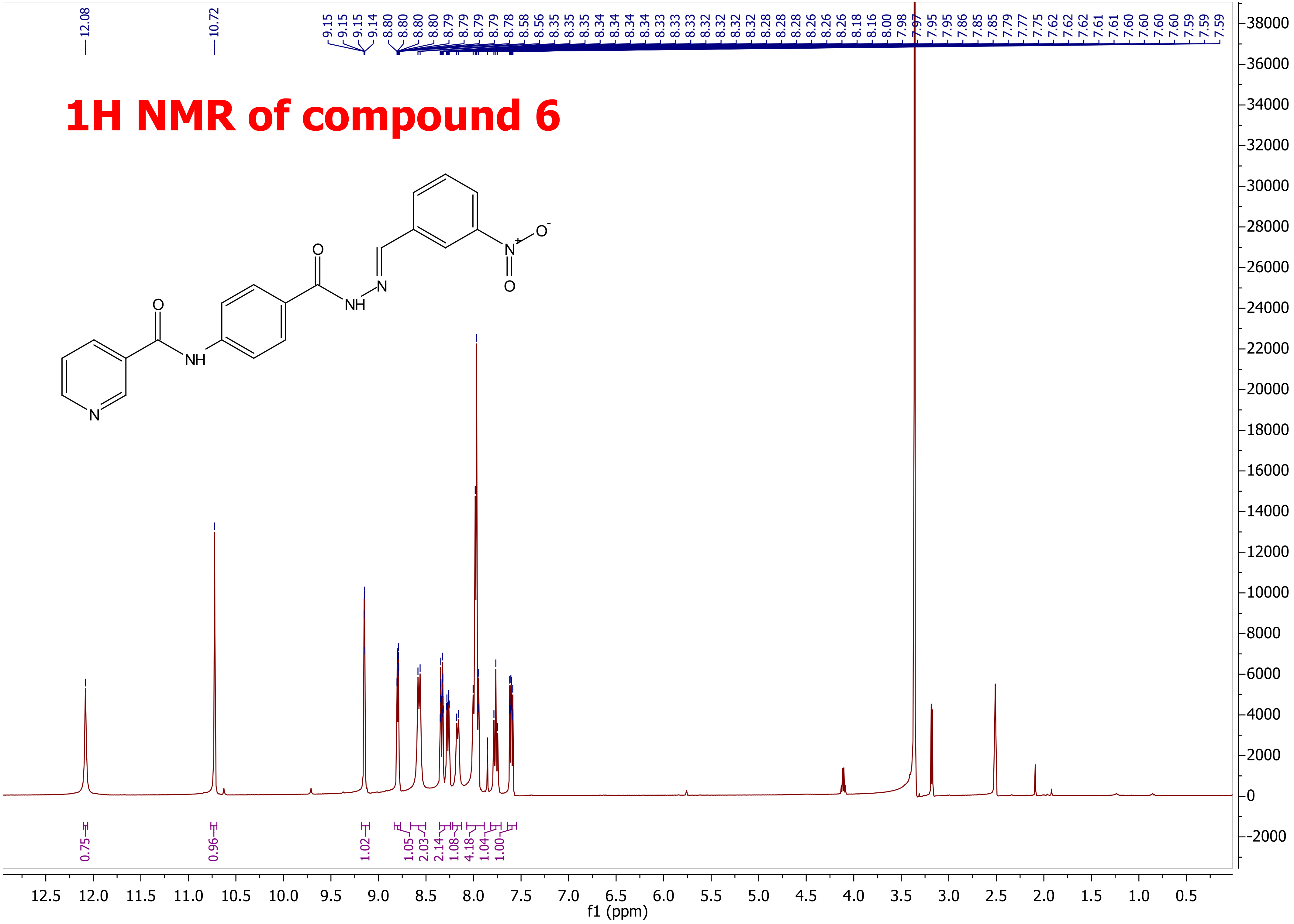

1H NMR of compound 6
12.08
10.72
9.15 9.15 9.15 9.14 8.80 8.80 8.80 8.80 8.79 8.79 8.79 8.79 8.78 8.58 8.56 8.35 8.35 8.35 8.34 8.34 8.34 8.34 8.33 8.33 8.33 8.32 8.32 8.32 8.32 8.28 8.28 8.28 8.26 8.26 8.26 8.18 8.16 8.00 7.98 7.97 7.95 7.95 7.86 7.85 7.85 7.79 7.77 7.75 7.62 7.62 7.62 7.61 7.61 7.60 7.60 7.60 7.59 7.59 7.59
O
NH
N
O
NH
N
O
N+
O-
O
0.75
0.96
1.02
1.05
2.03
2.14
1.08
4.18
1.04
1.00
38000
36000
34000
32000
30000
28000
26000
24000
22000
20000
18000
16000
14000
12000
10000
8000
6000
4000
2000
0
-2000
12.5 12.0 11.5 11.0 10.5 10.0 9.5 9.0 8.5 8.0 7.5 7.0 6.5 6.0 5.5 5.0 4.5 4.0 3.5 3.0 2.5 2.0 1.5 1.0 0.5
f1 (ppm)

1H NMR of compound 6

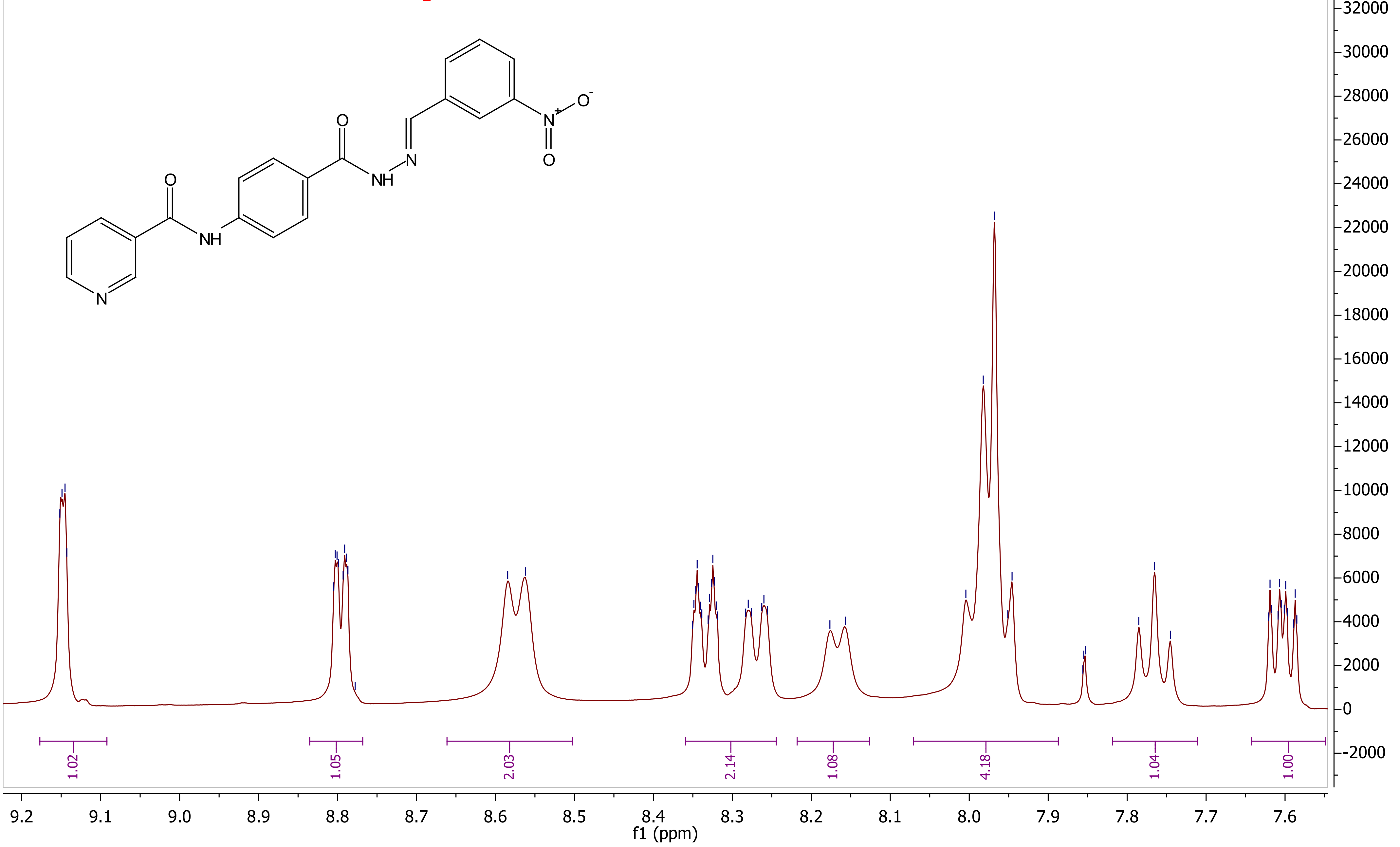

13C NMR of compound 6

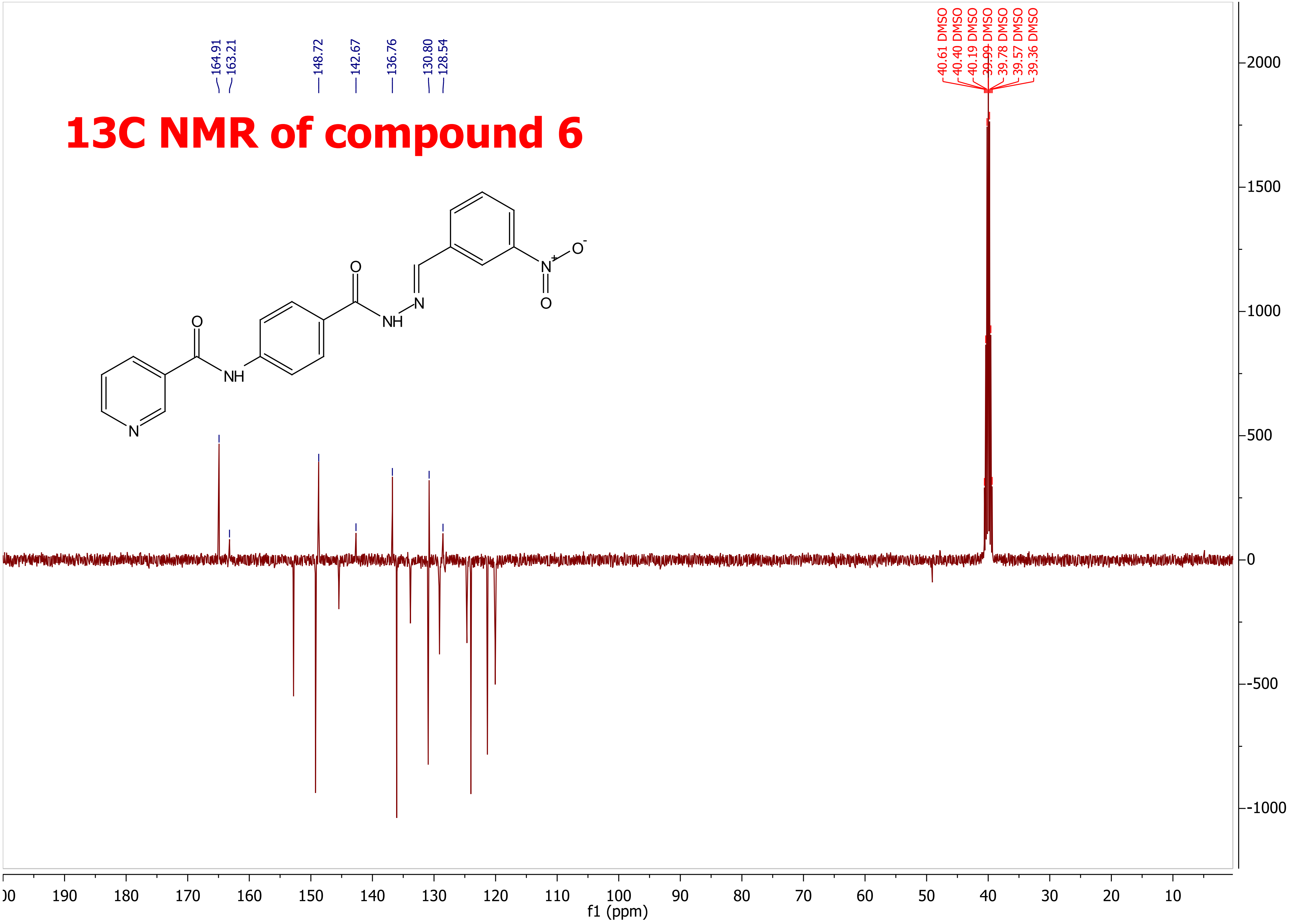

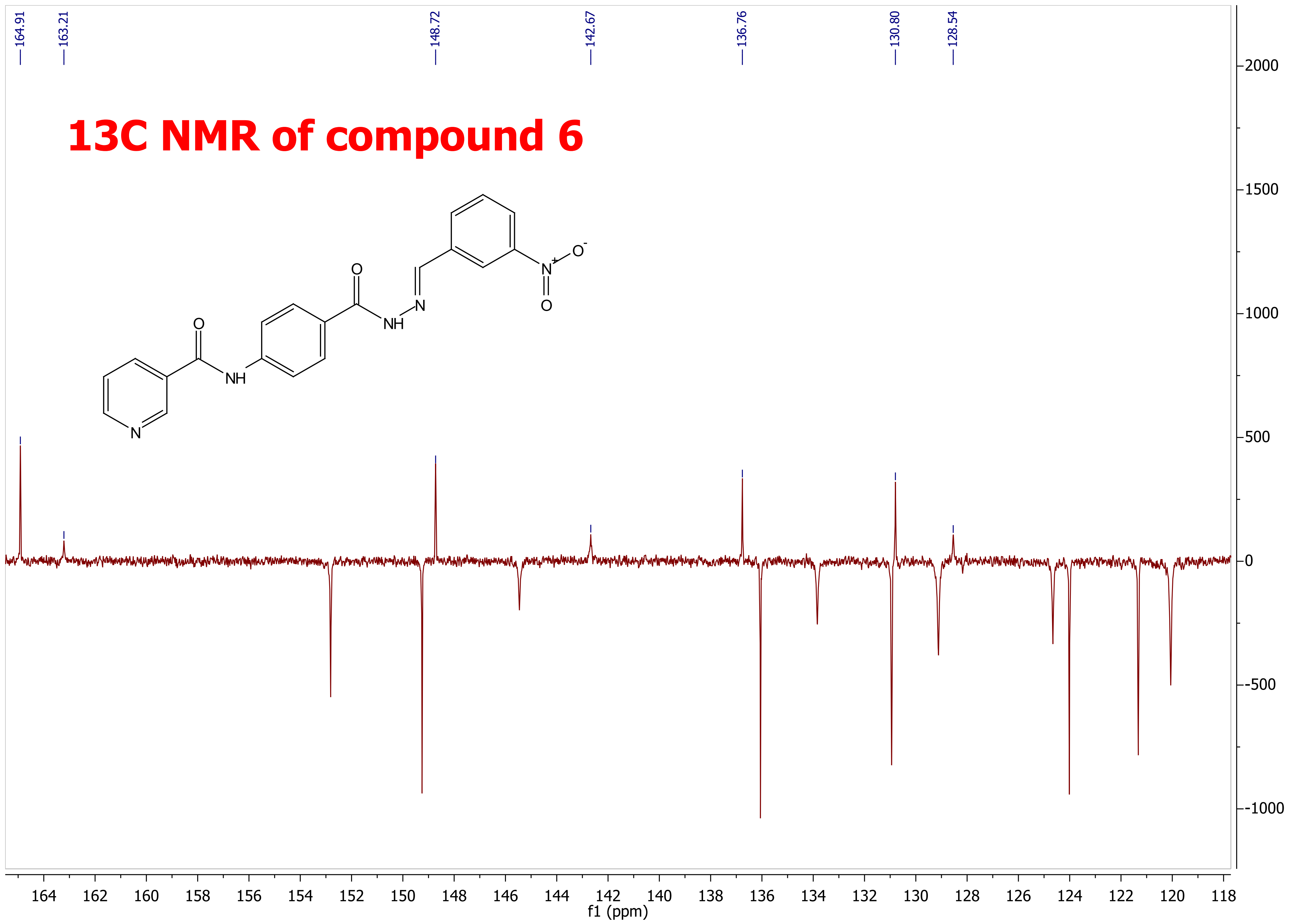

164.91
163.21
148.72
142.67
136.76
130.80
128.54
13C NMR of compound 6
NH
NH
N
N
O
O
O
N
O
2000
1500
1000
500
0
-500
-1000
164
162
160
158
156
154
152
150
148
146
144
142
140
138
136
134
132
130
128
126
124
122
120
118
f1 (ppm)

IR of compound 7

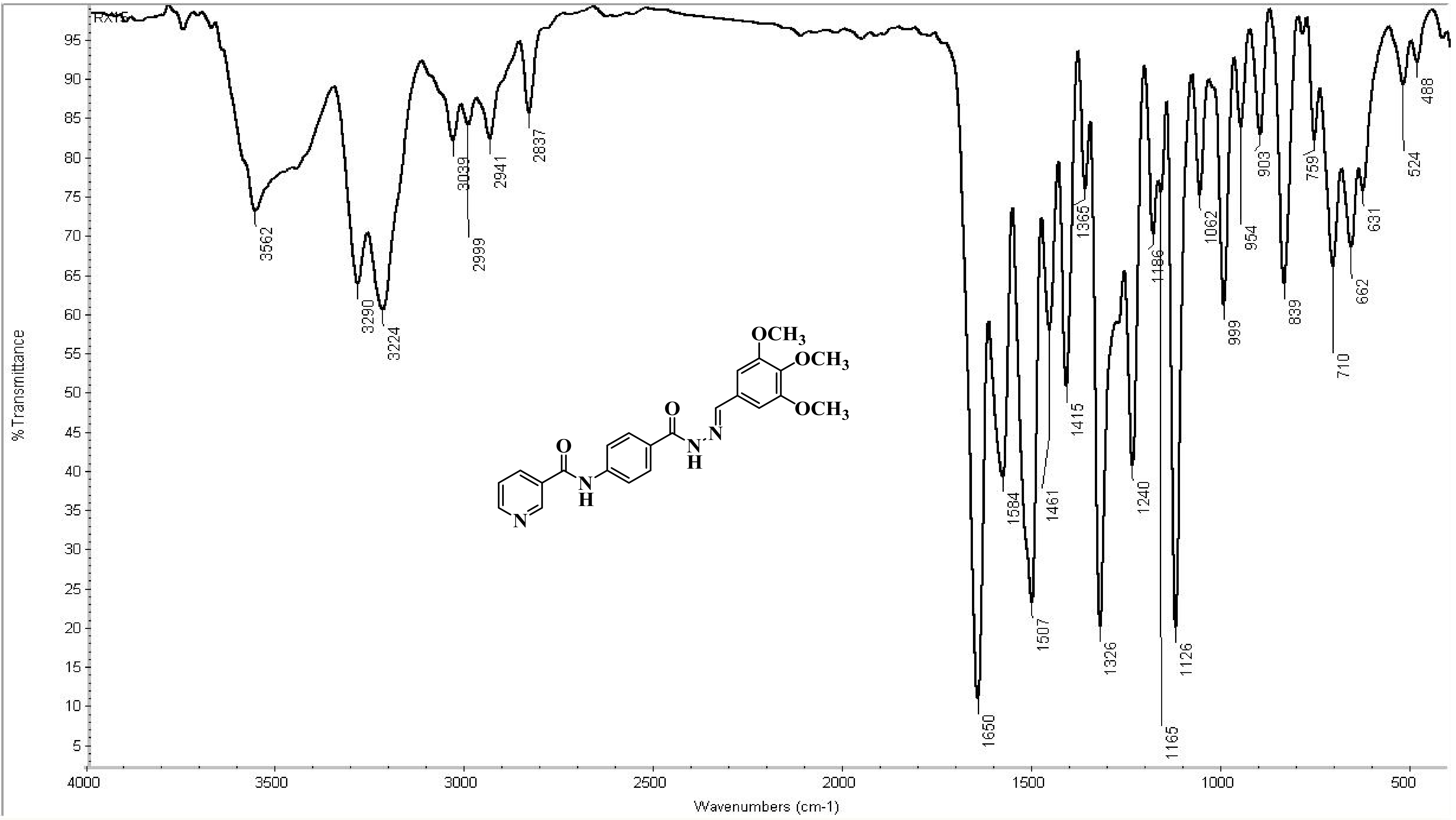

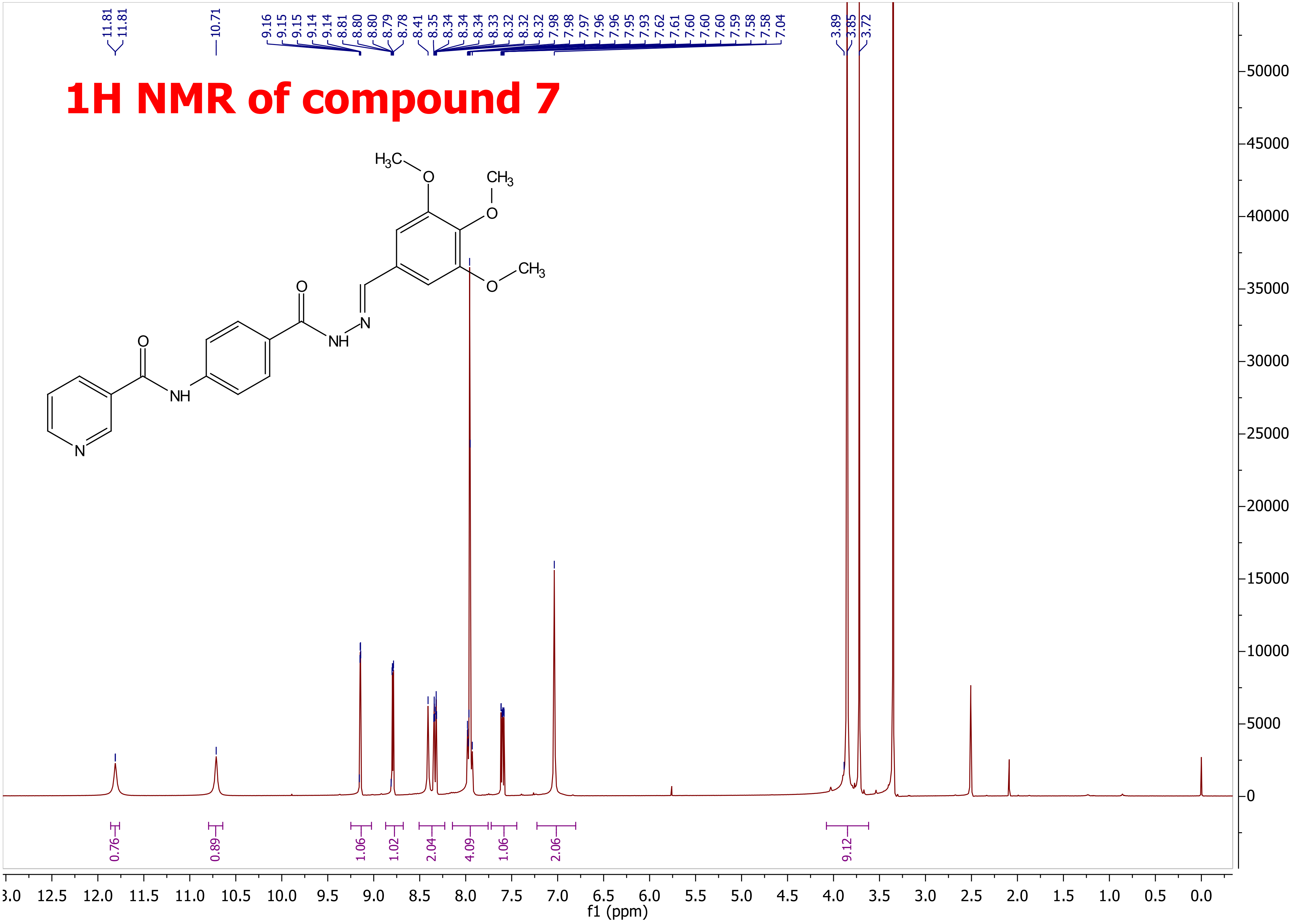

1H NMR of compound 7
11.81
11.81
10.71
9.16
9.15
9.15
9.14
9.14
8.81
8.80
8.80
8.79
8.78
8.41
8.35
8.34
8.34
8.34
8.33
8.32
8.32
8.32
7.98
7.98
7.97
7.96
7.96
7.95
7.93
7.62
7.61
7.60
7.60
7.60
7.59
7.58
7.58
7.04
3.89
3.85
3.72
H3C
O
CH3
O
CH3
O
O
N
NH
O
NH
N
0.76
0.89
1.06
1.02
2.04
4.09
1.06
2.06
9.12
f1 (ppm)

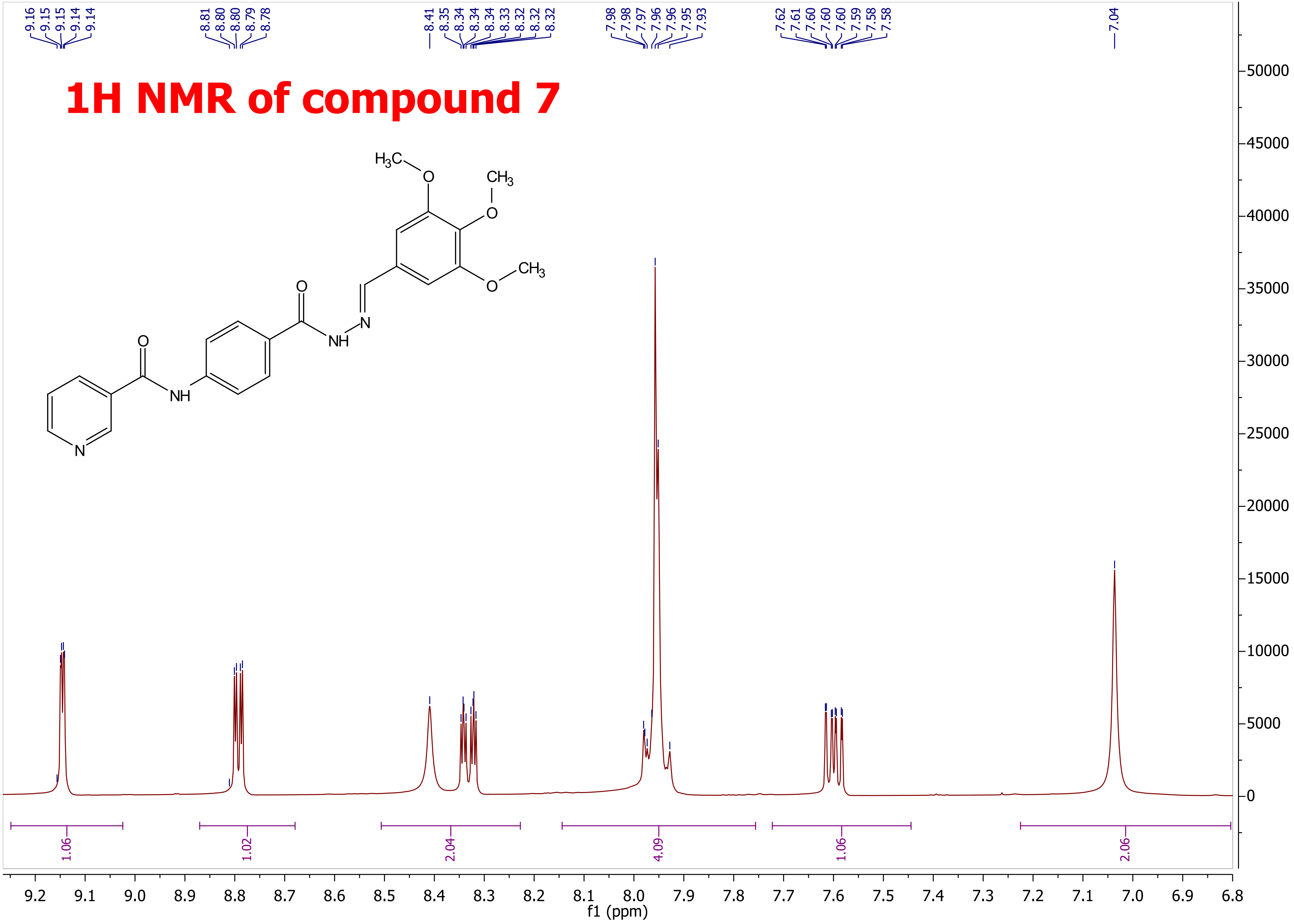

1H NMR of compound 7
9.16 9.15 9.15 9.14 9.14
8.81 8.80 8.80 8.79 8.78
8.41 8.35 8.34 8.34 8.34 8.33 8.32 8.32 8.32
7.98 7.98 7.97 7.96 7.96 7.95 7.93
7.62 7.61 7.60 7.60 7.60 7.59 7.58 7.58
7.04
1.06
1.02
2.04
4.09
1.06
2.06
f1 (ppm)

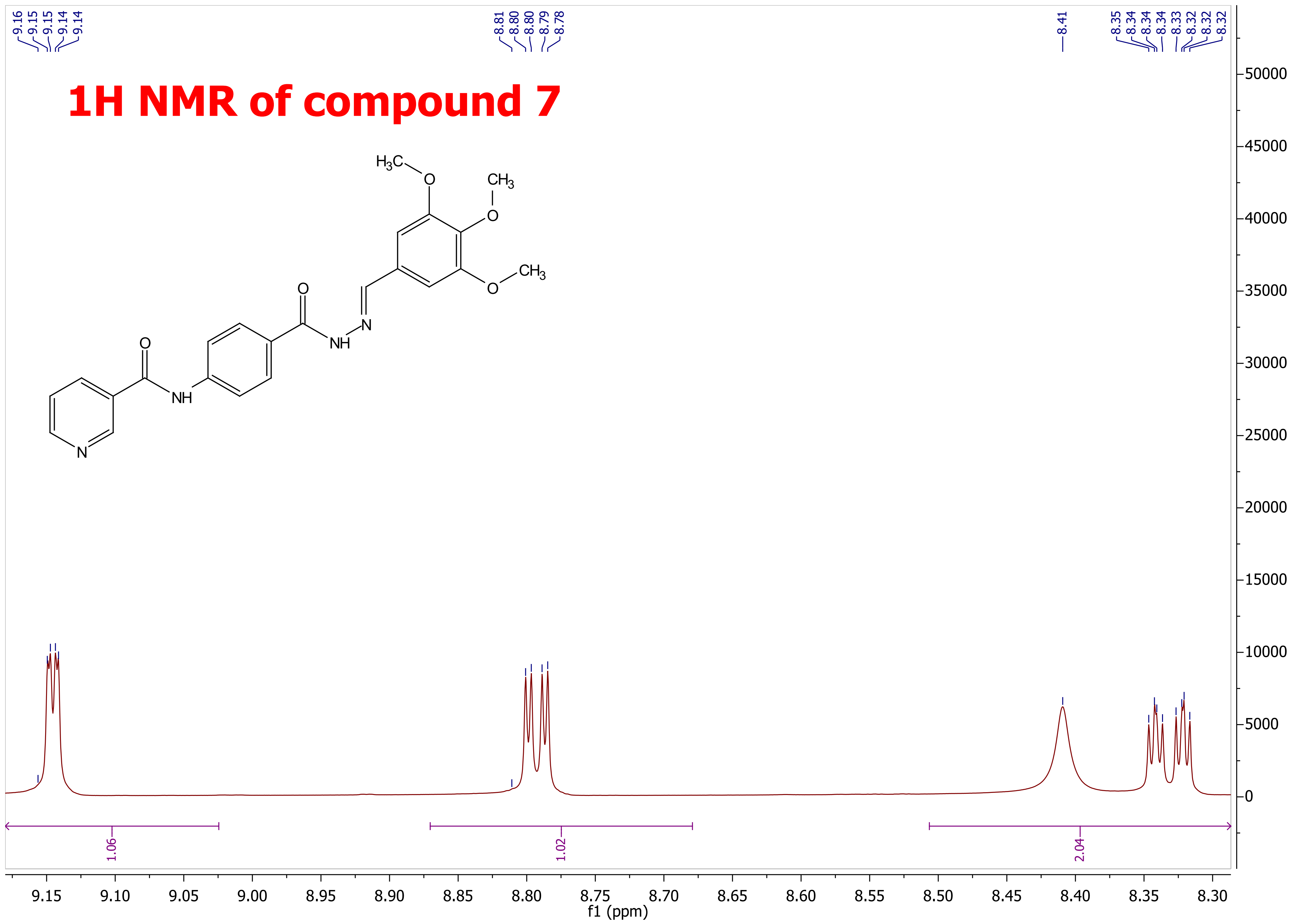
1H NMR of compound 7
9.16
9.15
9.15
9.14
9.14
8.81
8.80
8.80
8.79
8.78
8.41
8.35
8.34
8.34
8.34
8.33
8.32
8.32
8.32
1.06
1.02
2.04
f1 (ppm)

13C NMR of compound 7

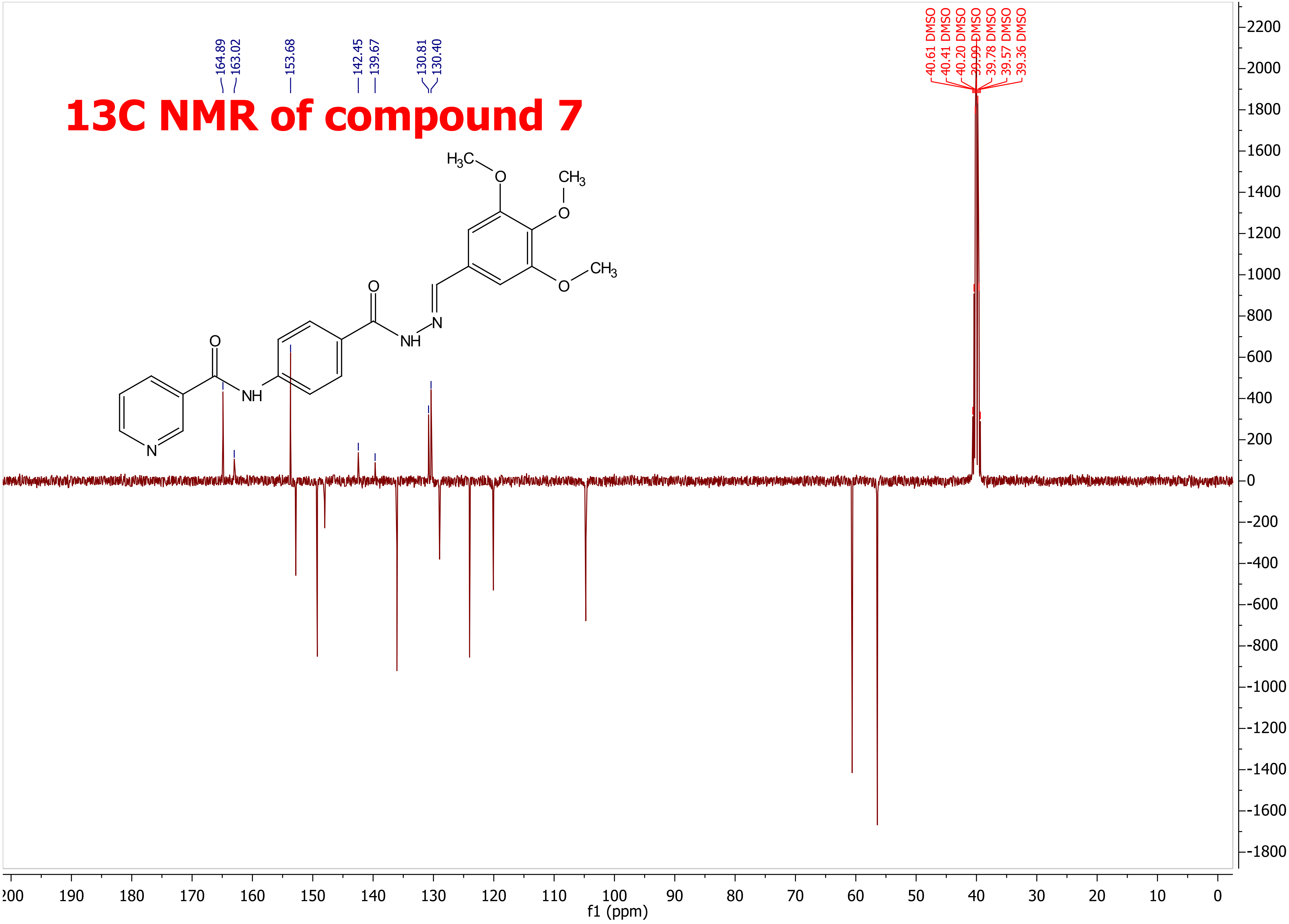

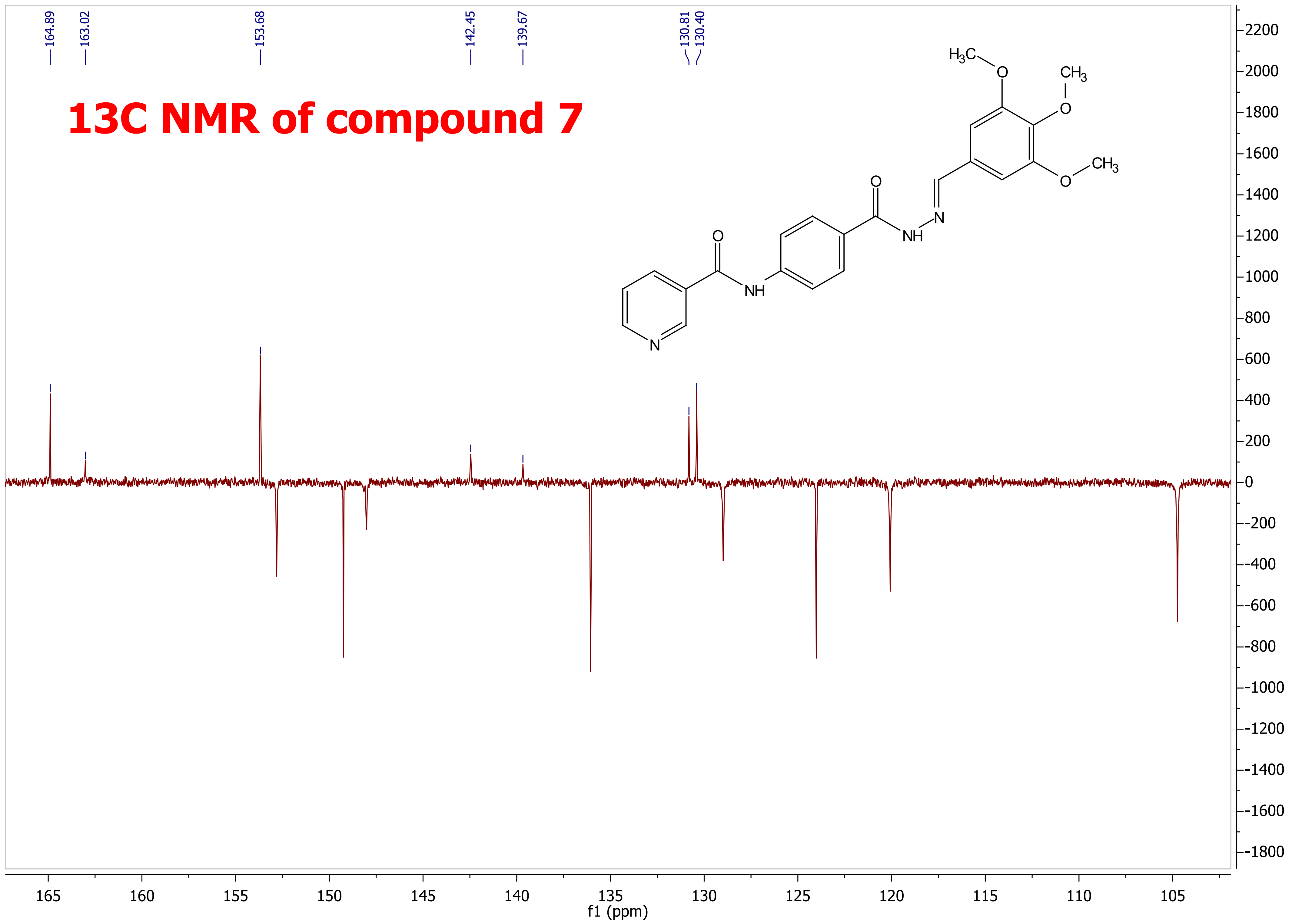
13C NMR of compound 7
164.89
163.02
153.68
142.45
139.67
130.81
130.40
H3C
CH3
O
NH
N
f1 (ppm)

13C NMR of compound 7

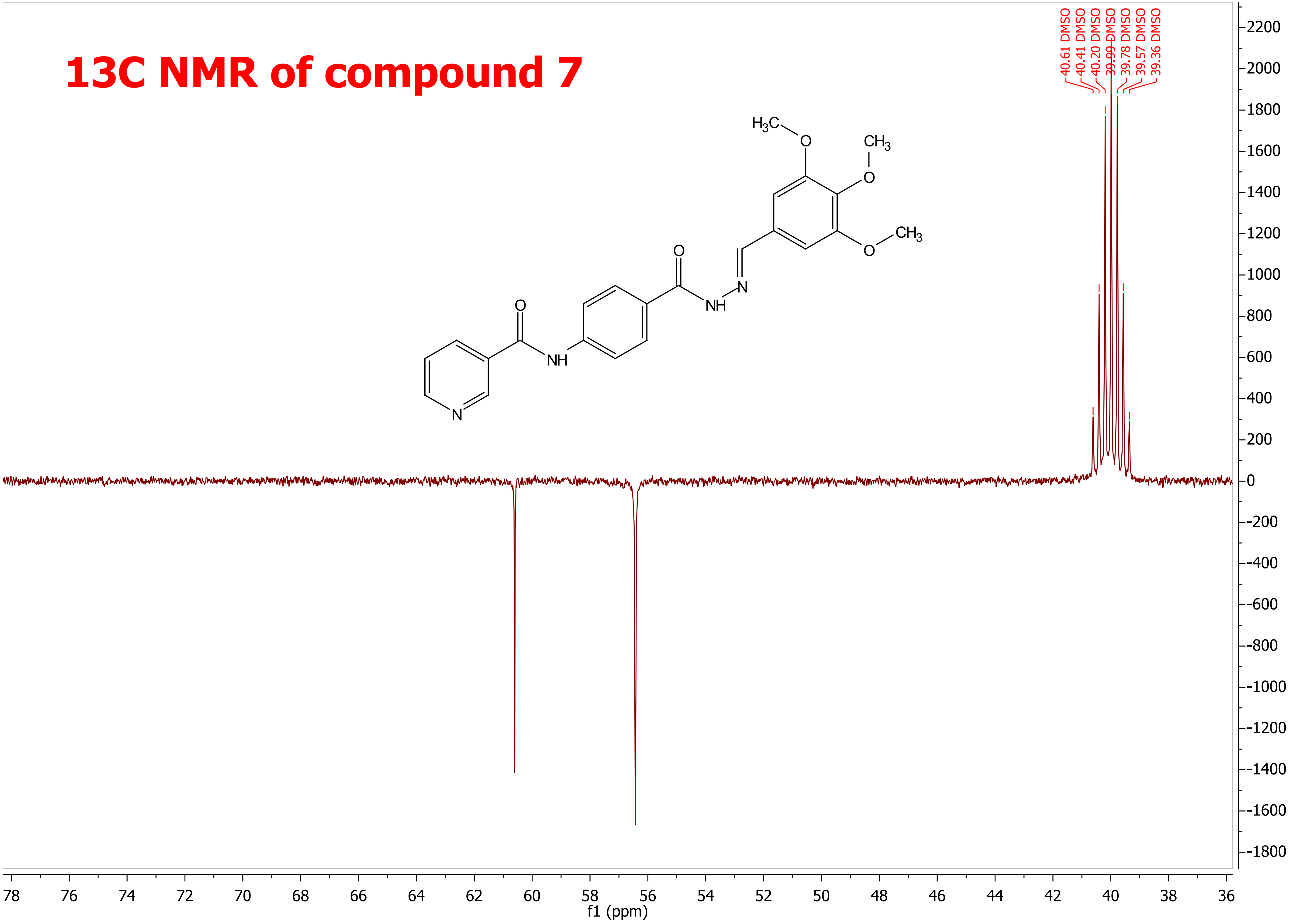

IR of compound 10

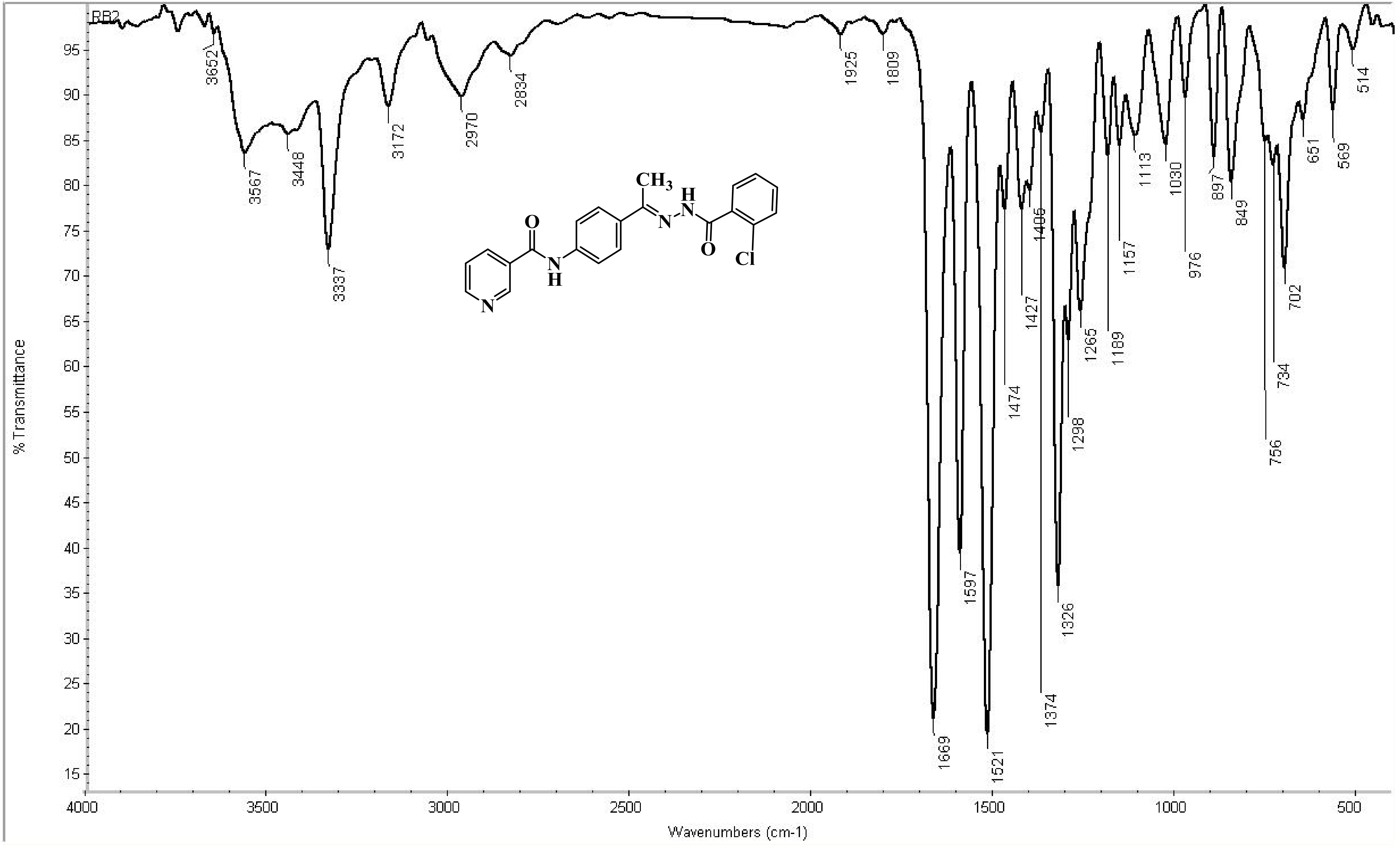

1H NMR of compound 10

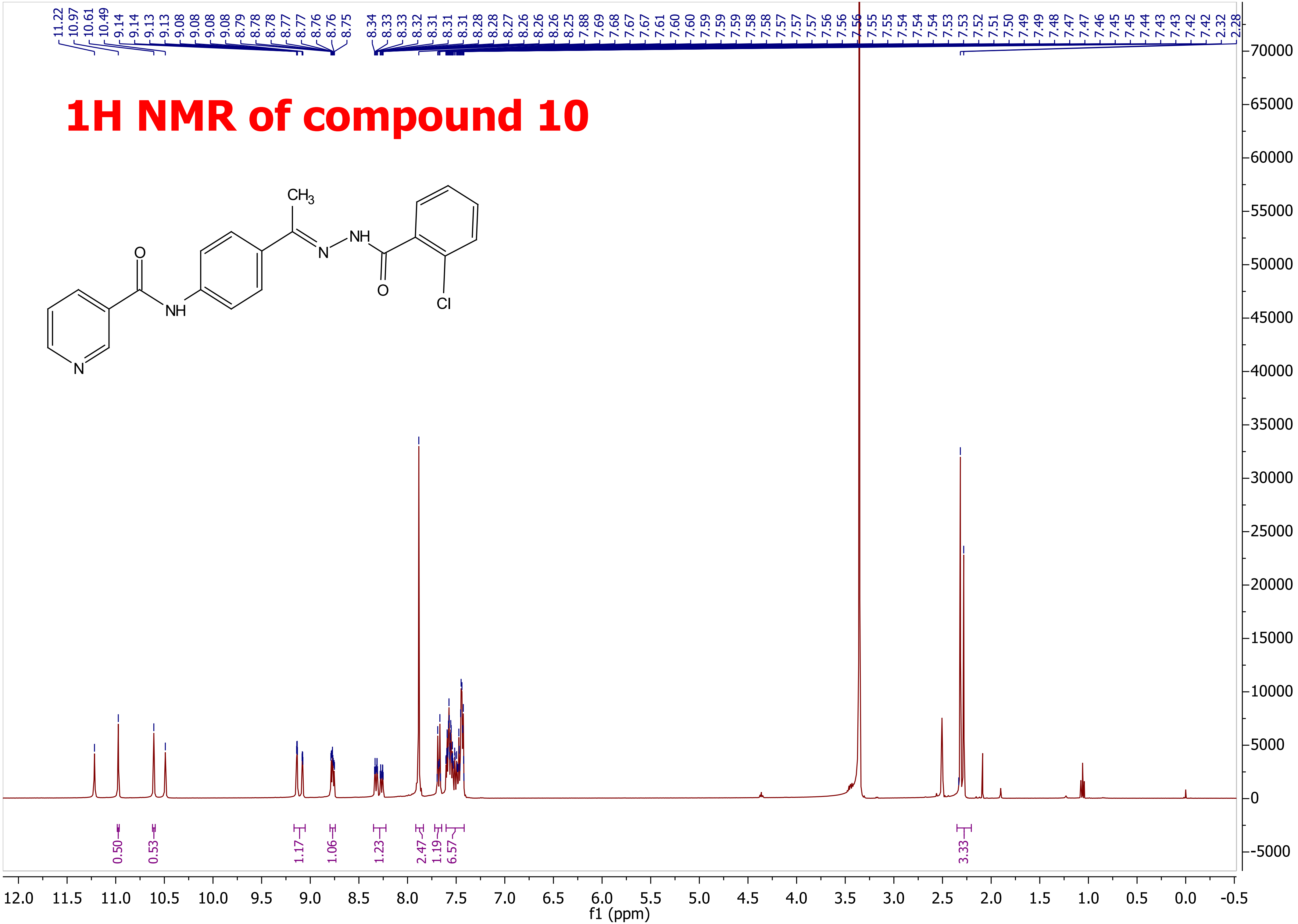

1H NMR of compound 10

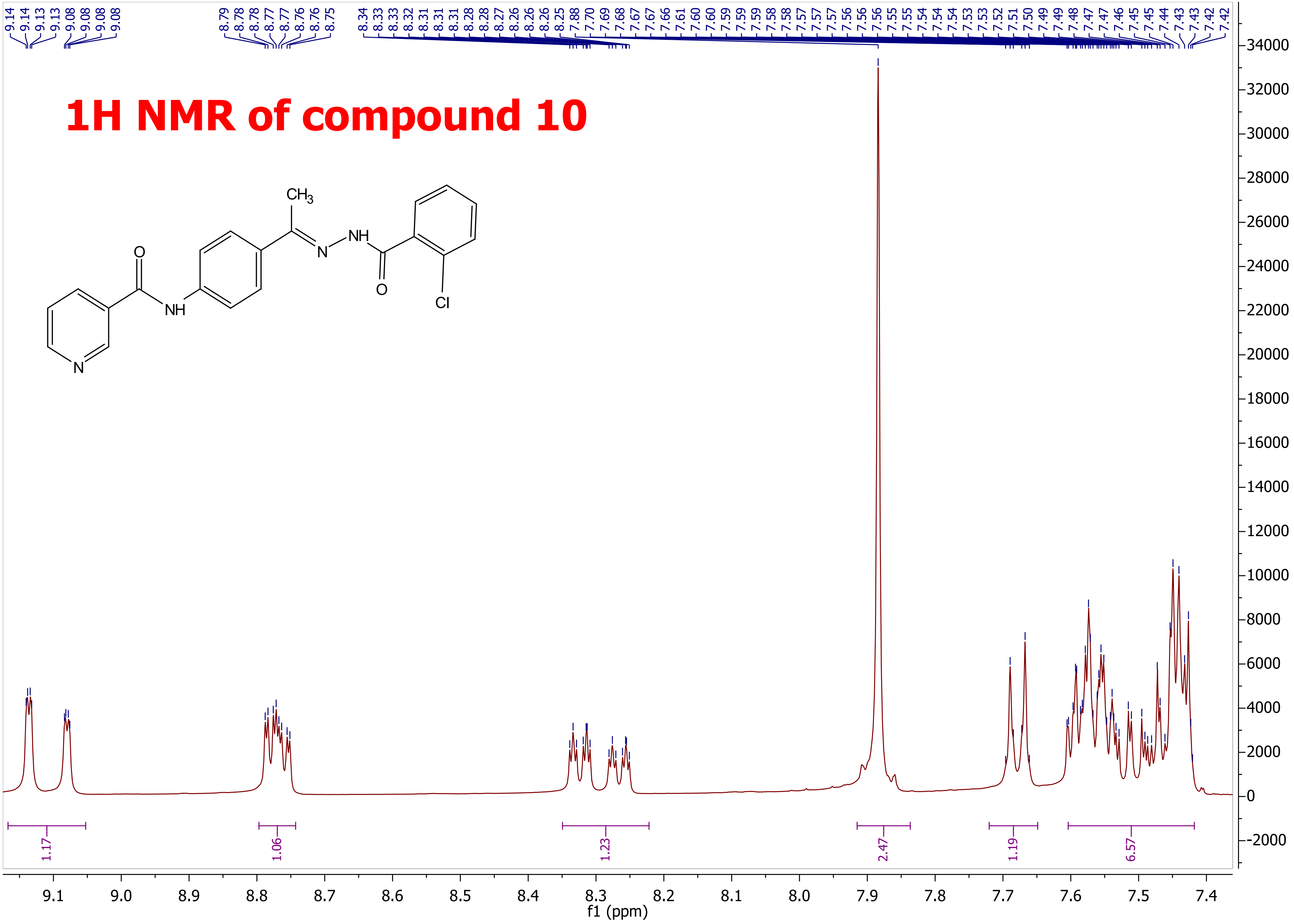

13C NMR of compound 10

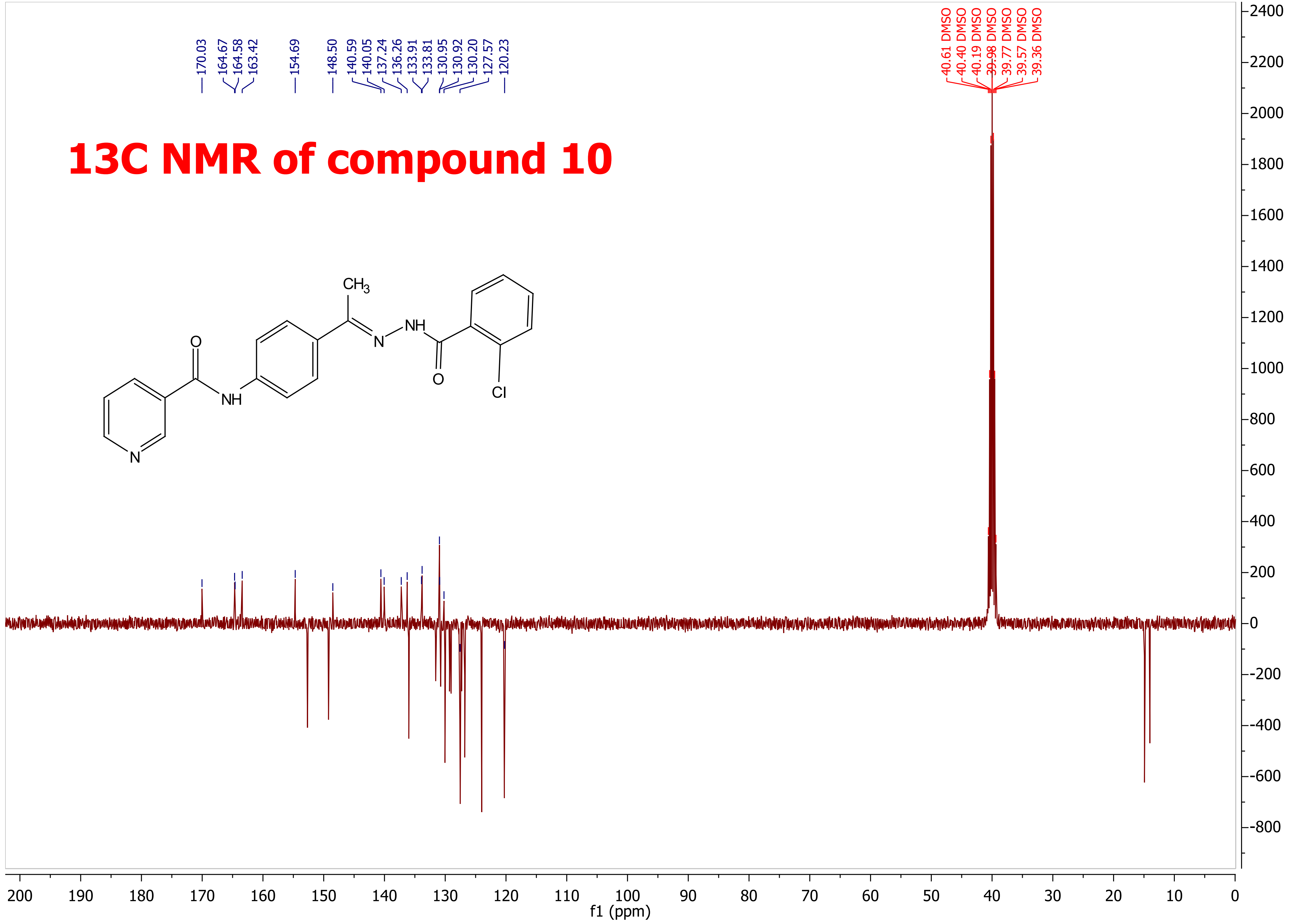

13C NMR of compound 10

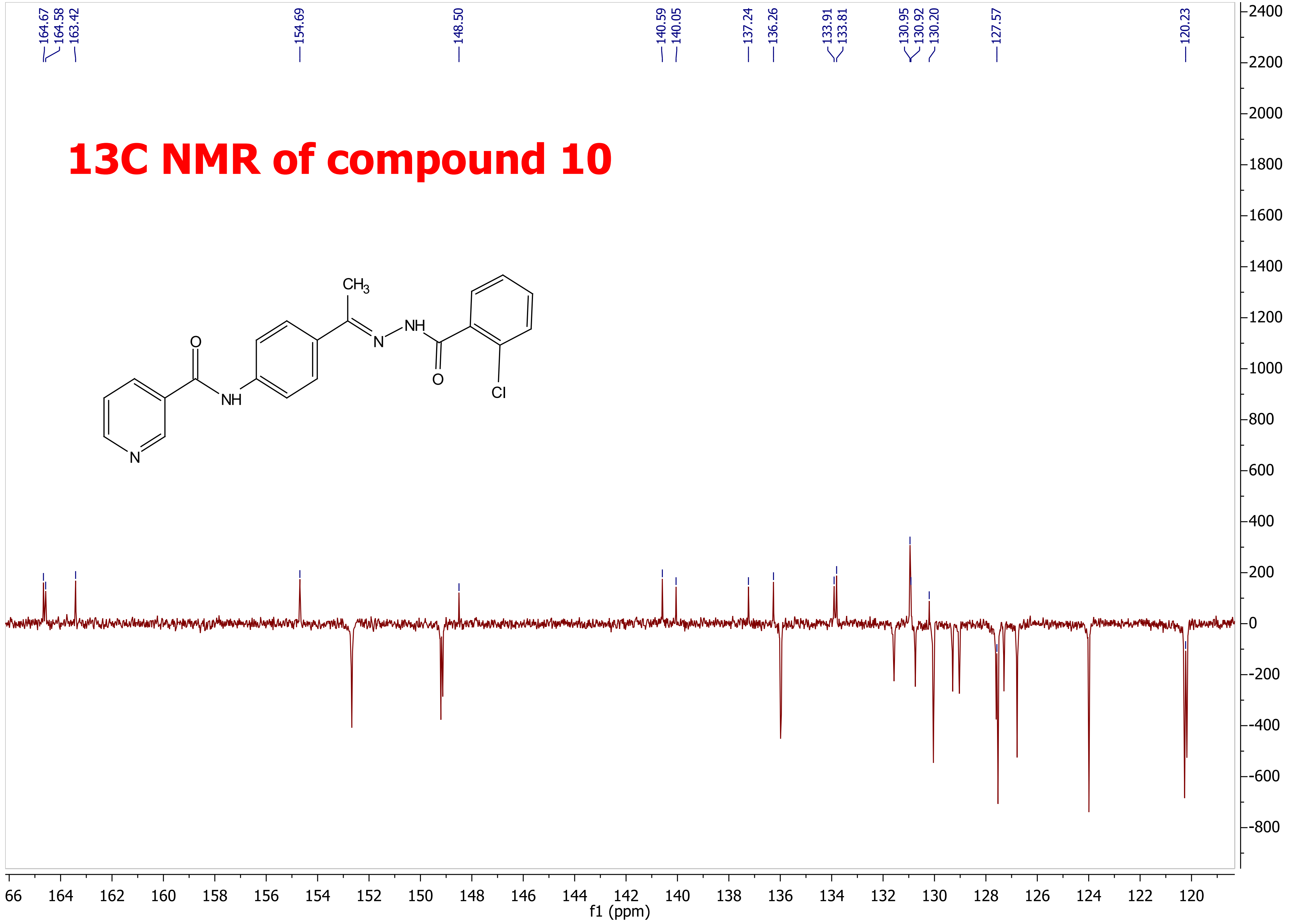

IR of compound 11

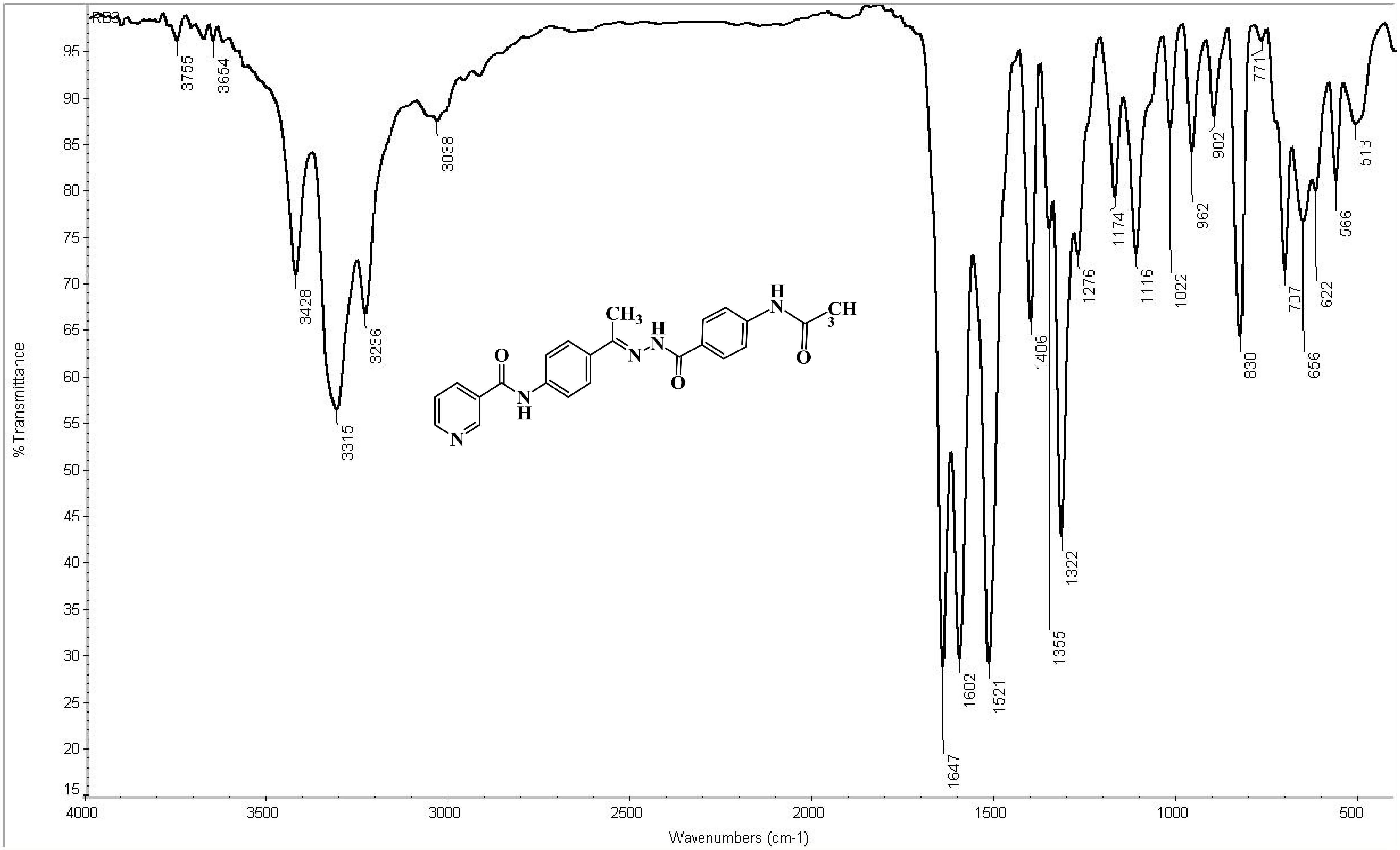

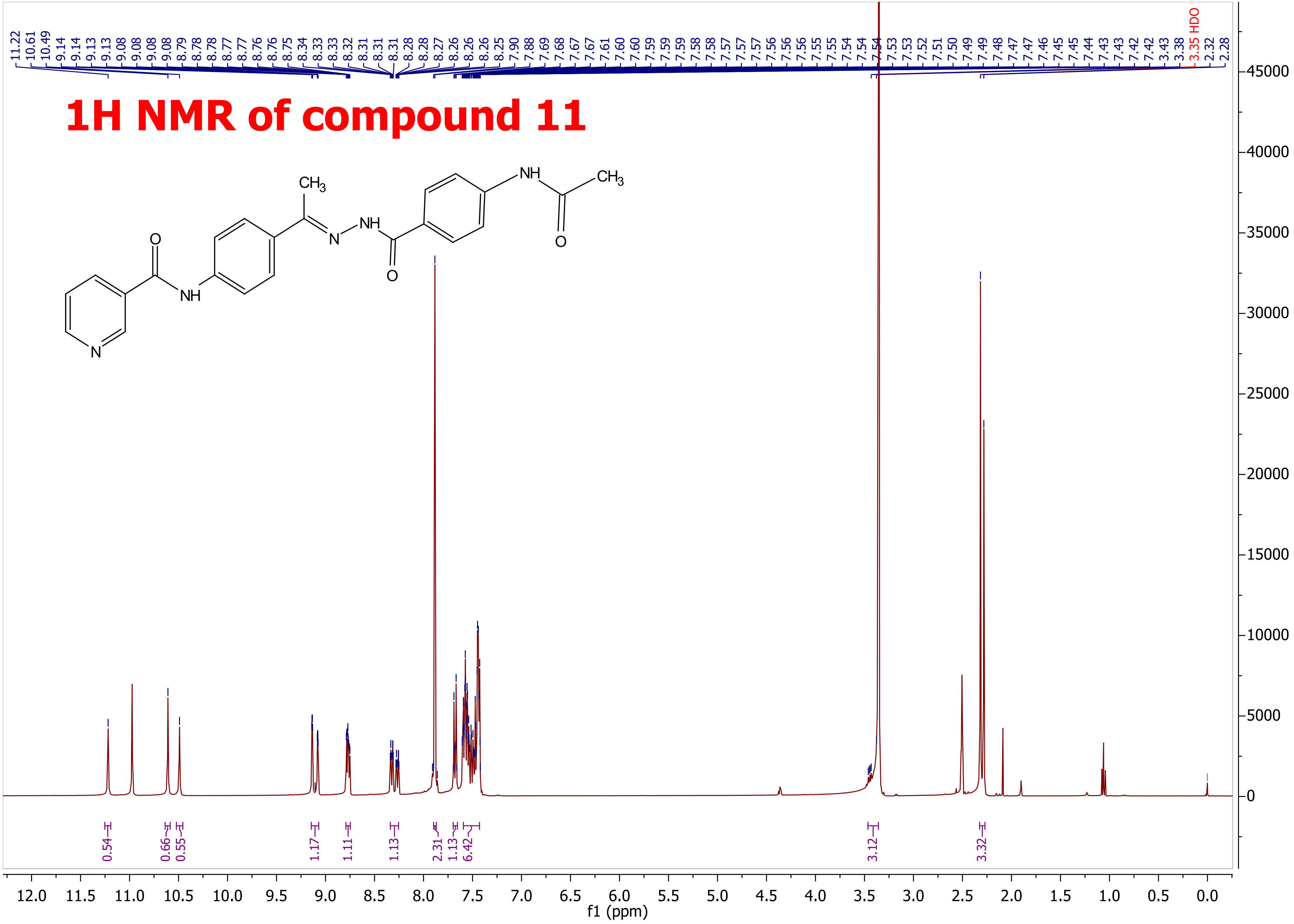
1H NMR of compound 11
f1 (ppm)
3.35 HDO

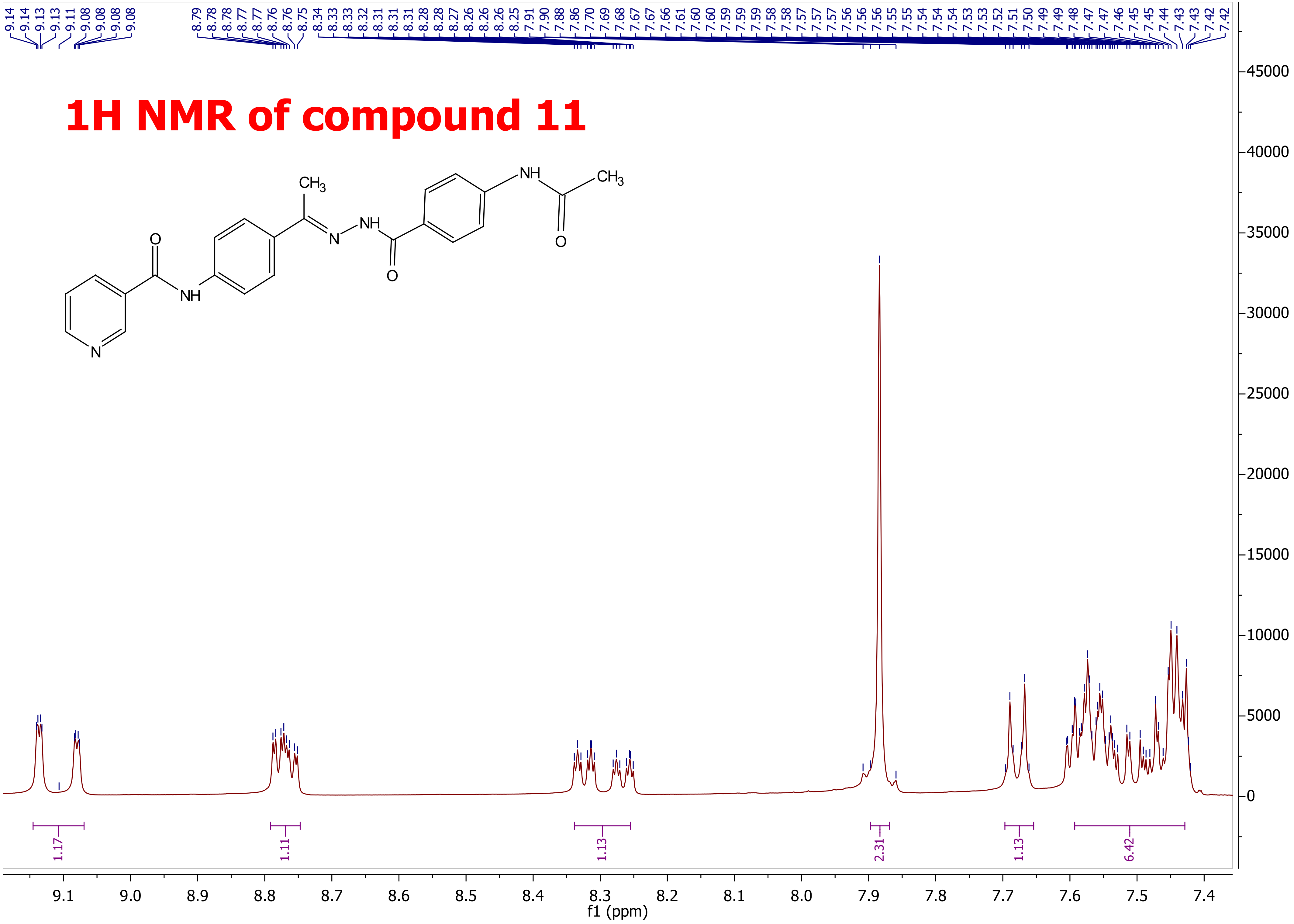
1H NMR of compound 11
9.14 9.14 9.13 9.13 9.11 9.08 9.08 9.08 9.08 8.79 8.78 8.78 8.77 8.77 8.76 8.76 8.75 8.34 8.33 8.33 8.32 8.31 8.31 8.31 8.28 8.28 8.27 8.26 8.26 8.26 8.25 7.91 7.90 7.88 7.86 7.70 7.69 7.68 7.67 7.67 7.66 7.61 7.60 7.60 7.59 7.59 7.59 7.58 7.58 7.57 7.57 7.57 7.56 7.56 7.55 7.55 7.54 7.54 7.54 7.53 7.53 7.52 7.51 7.50 7.49 7.49 7.48 7.47 7.47 7.46 7.45 7.45 7.44 7.43 7.43 7.42 7.42
CH3
NH
CH3
O
NH
N
NH
O
O
N
1.17
1.11
1.13
2.31
1.13
6.42
f1 (ppm)

13C NMR of compound 11

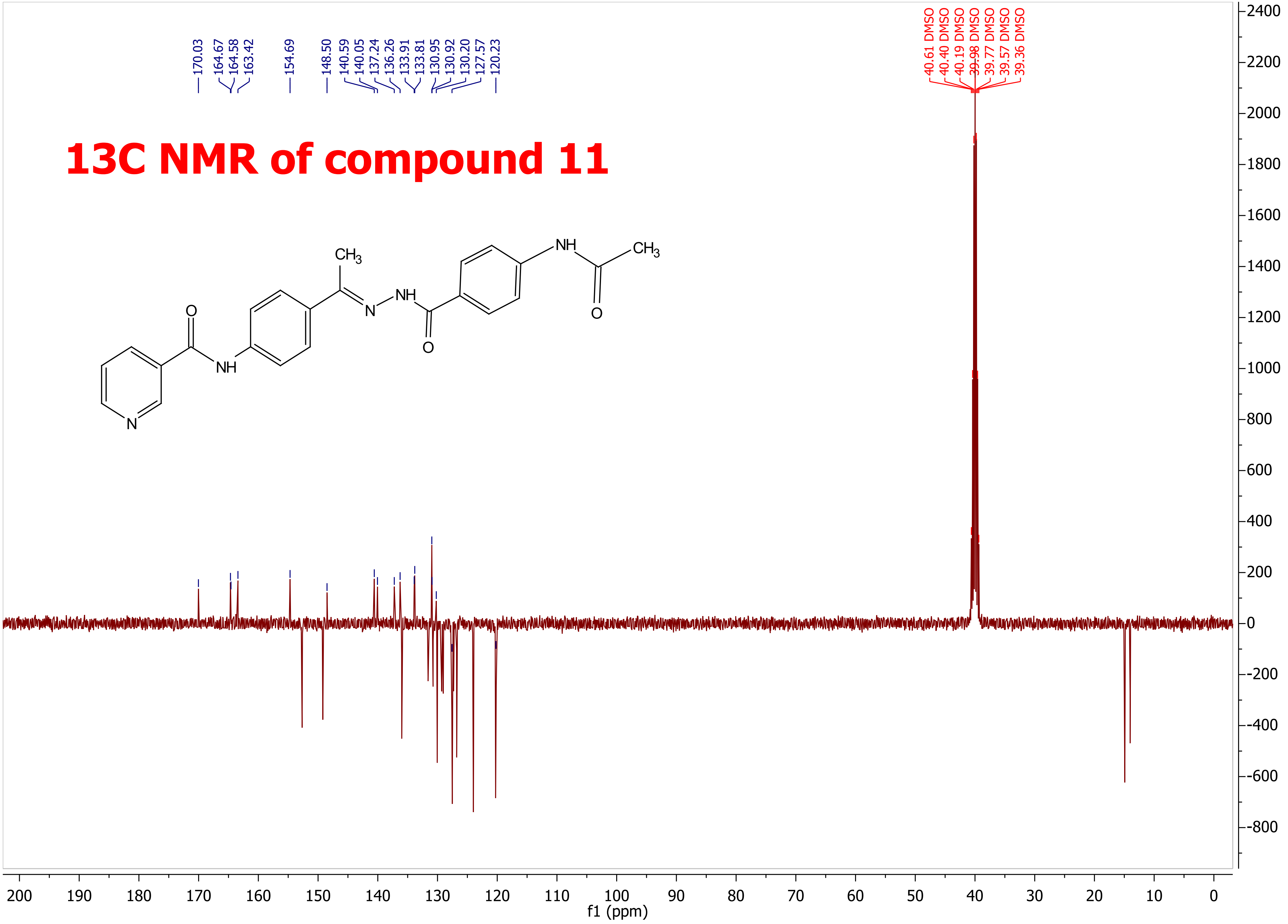

13C NMR of compound 11

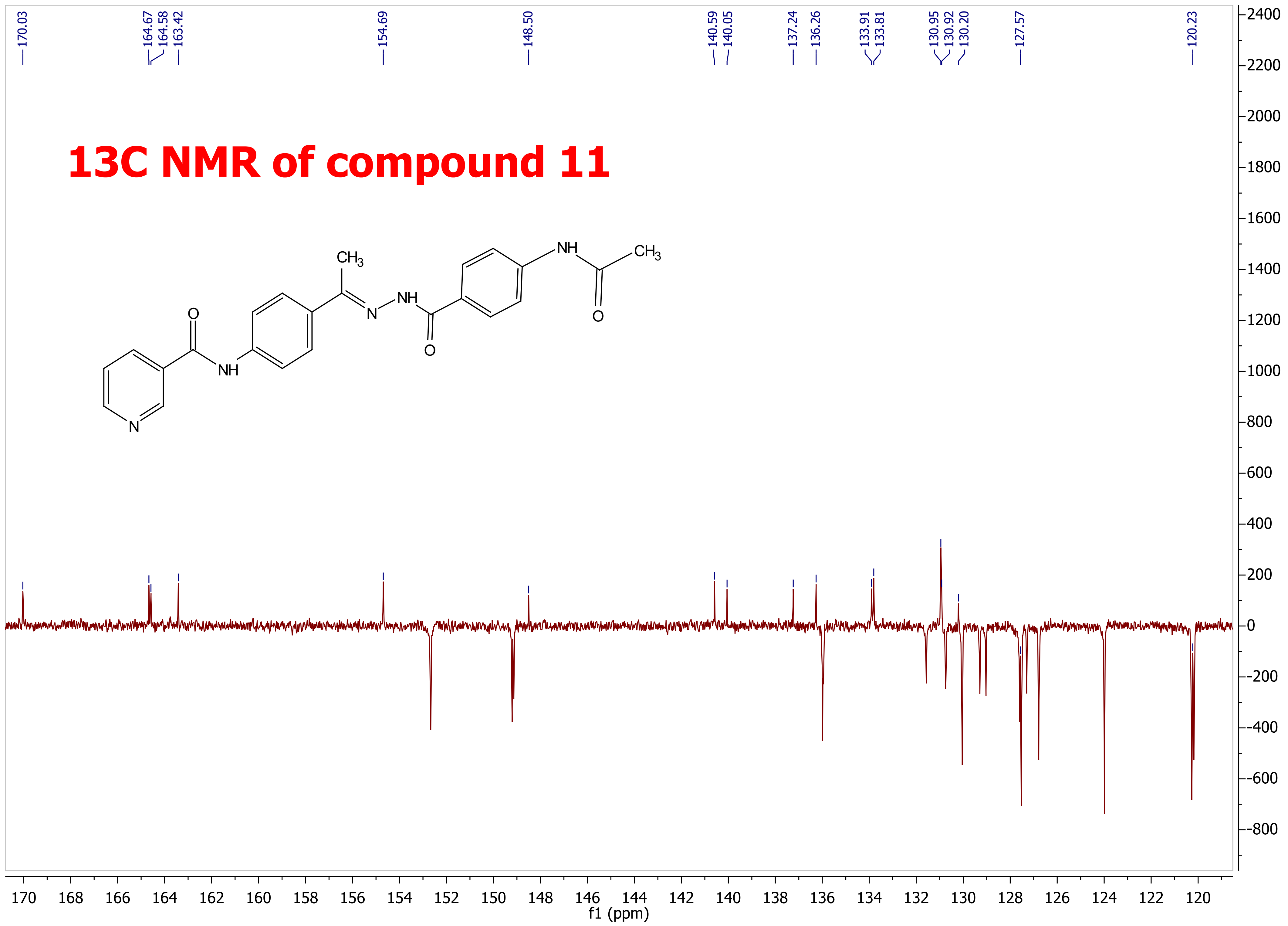

3- 4.2. Experimental of biological testing

4.2.1. In vitro anti-proliferative activity

The in vitro antiproliferative activities of the synthesized compounds were evaluated against two human tumor cell lines: hepatocellular carcinoma (HepG2) and breast cancer (MCF-7) using MTT assay protocol The commercially available sorafenib were used in this test as a positive control. The tested cell lines were purchased and dropped on the appropriate growth medium. The growth medium was supplemented with 100 mg/mL of streptomycin, 100 units/mL of penicillin and 10% of heat-inactivated fetal bovine serum in a humidified 5% (v/v) CO2 atmosphere at 37 ◦C. Then the cells from the two cancer cell lines were seeded at the appropriate densities into 96-well microtiter plates. After incubation for 24 h, the growth medium of each cell was treated with graded concentrations (0.1, 10, 100 and 1000 μM) of the test compounds and incubated for three days. The viability of treated cells was determined using 3-[4,5-dimethylthiazole-2-yl]- 2,5-diphenyltetrazolium bromide (MTT) technique as cells were stained with 5% MTT solution and allowed to break down the dye into colored-insoluble formazan crystals for 4 h. The formazan crystals were dissolved in acidified isopropanol for 30 min with continuous shaking at room temperature. The colorimetric assay was measured and recorded at absorbance of 570 nm. The cell viability was expressed as percentage of control and the concentration that induces 50% of maximum inhibition of cell proliferation considering control group as 100% viability and group treated with a mixture of toxic compounds as 0% viability. (IC_{50}) were determined for each compound using Graph Pad Prism version 5 software by plotting of log C against % viability.

4.2.2. In vitro VEGFR-2 kinase assay

The synthesized compounds were estimated for their *in vitro* inhibition on human VEGFR-2 in HepG2 cell line; using ELISA kit. Firstly, a plate was used for the assay had been coated by an antibody specific for human VEGFR-2 enzyme, Sorafenib was nominated as a standard VEGFR-2 inhibitor. Both standard and samples were added to the wells and incubated overnight at 4 ◦C, then washed. The biotinylated antibody was supplemented and further incubated for 1 h at room temperature. The unreacted,

liberated antibody was then washed; followed by addition of HRP-conjugated streptavidin and incubated for 45 min at room temperature. Wells were washed and a TMB substrate solution was added and kept at room temperature for 30 min. Finally, the stop solution was added, and the intensity of the color produced was measured at 450 nm . Concentration-inhibition response curve was established by GraphPad Prism 5.0. The IC_{50} value was calculated as the concentration at which 50% of the cells could survive in comparison to sorafenib.

4.2.3. Flow cytometry analysis for cell cycle

Cell cycle analysis for the most potent candidate **7** was carried out through Flow cytometric analysis. In this test HepG2 cells were supplemented with the test compound at its cytotoxic concentration, seeded and subjected for incubation for 24 h at 37 ◦C and 5% CO_2. Cells were washed twice with phosphate buffer saline, then the centrifugation of cell pellets had been completed, followed by preservation with ice-cold 70% ethanol for 15 min. Pellets were collected again and incubated with propidium iodide (PI) staining solution. After incubation for one hour at room temperature, it was analyzed by flowcytometry on an FC500 cytometer (Beckman Coulter) and the cell cycle distributions were calculated.

4.2.4. Flow cytometry analysis for apoptosis

Flow cytometry cell apoptosis analysis was used to investigate the apoptotic effect of the most active compound **7** in HepG2 cells, the examination depends on that AnnexinV-fluorescein isothiocyanate (Annexin V-FITC) is a protein that has high affinity to phosphatidyl serine PS, which can be detected by staining with Annexin V-(FITC) and counter staining with propidium iodide (PI). In such procedure, HepG2 cells were incubated together with the tested compound (100 mL) for 24 h. Then, the cells were centrifuged, collected by trypsin, washed with PBS two successive times, and suspended in 500 mL of binding buffer. The double staining Annexin V-FITC (5 mL) and PI (5 mL) were added to the cells and incubated in the dark for 5 min at room temperature. The stained cells were analyzed for flow cytometry using FACS caliber flow cytometer and apoptosis detection kit (BD Biosciences, San Jose, CA)

4.2.5. Quantitative Real Time Reverse-Transcriptase PCR technique

The quantity of immunomodulatory proteins (TNF-α and IL-6) in control and compounds **7**, **10** (at the IC_{50} concentration)-treated HepG2 cells was assessed by qRT-PCR (reference). Total RNA from vehicle-treated control (0.01% DMSO) and 10k-treated HepG2 cells were extracted as-per the manufacturer instructions (RNeasy mini kit, Qiagen, Germany). After RNA extraction, cDNA was prepared using the Revert Aid First Strand cDNA Synthesis kit (Thermo Scientific, USA). Amplification of target cDNA for apoptosis markers and GAPDH [as a normalization (housekeeping) gene] was done using one-step RT-PCR SYBR® Green kit Master Mix (Bio-Rad Laboratories, USA) on Rotor-Gene Q real-time PCR thermal cycler instrument. cDNA (2 µl aliquots) was mixed with 1 µl of forward primer, 1 µl reverse primer, 10 µl master mixture, and the reaction volume was completed to 20 µl with nuclease-free water. All experiments were performed in triplicates

4- 4.3. Experimental of *in silico* studies

4.3.1. Docking studies

The docking studies were performed utilizing MOE.14 software to explore the binding mode of the synthesized compounds towards VEGFR-2 kinase. The 3D crystal structures of the target macromolecules VEGFR-2 were downloaded from the protein databank, http://www.pdb.org (PDB ID; 4ASD). Furtherly, the synthesized compounds were studied for molecular docking against immunomodulatory proteins TNF-α (PDB ID: 2AZ5) and IL-6 (PDB ID: 1ALU) to investigate interactions patterns toward these active proteins. To prepare the target proteins, water molecules were removed, and the valances of atoms were corrected through protonation of the whole molecule. Then energy minimization was carried out by applying CHARMM and MMFF94 force fields. After that, the active binding site was defined and prepared for docking. The validation process was performed by redocking the co-crystallized ligand. The designed compounds were drawn using ChemBioDraw Ultra 14.0 and saved as MDL-SD format. The sketched compounds were constructed from fragment libraries in MOE program, protonated, followed by energy minimization then prepared for docking. Docking process was carried through Triangle matcher placement inserted in compute window, and the scoring function was London dG. Ten conformers (poses) for each molecule were generated using genetic algorithm searches. The free energies and binding modes of the designed molecules against VEGFR-2 were determined. The most ideal pose was selected according to its binding free energy as well as its binding mode with target molecule.

- **Validation figure**

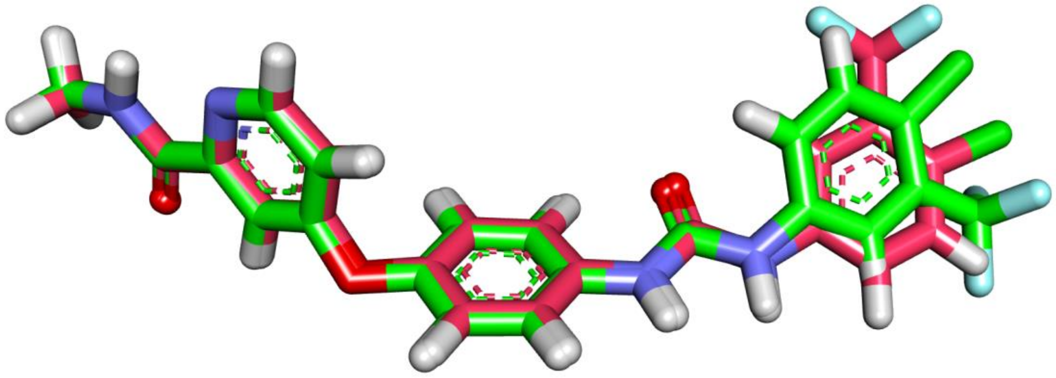

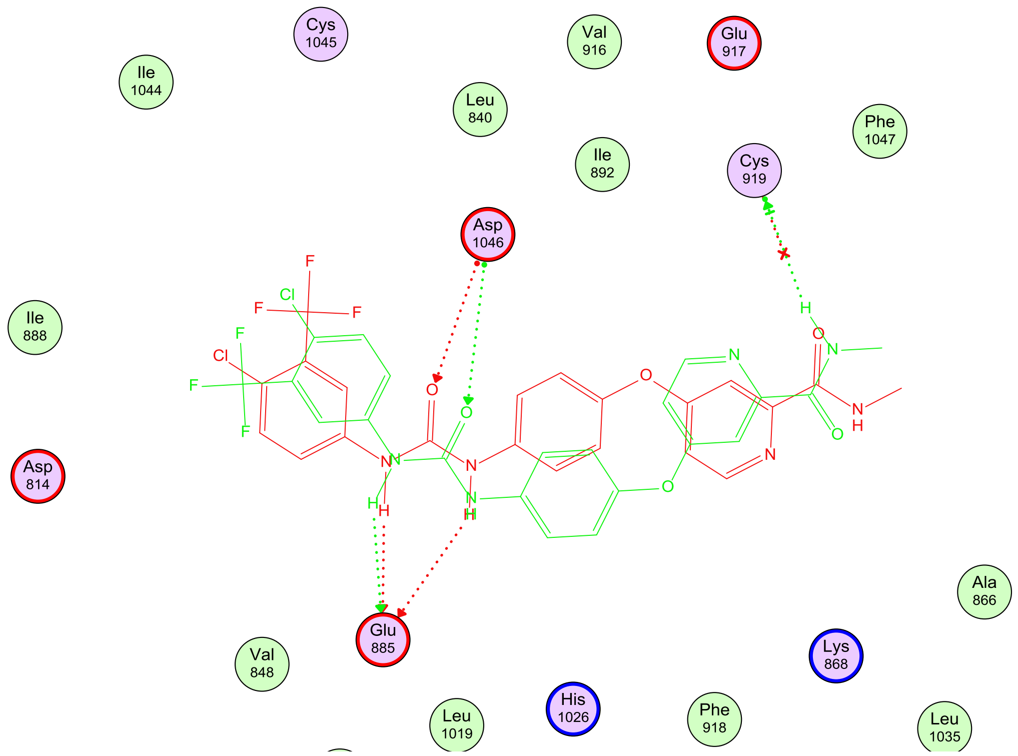

Fig. 7: The 3D image of the superimposition of the re-docked conformer of sorafenib over the co-crystallized one with an RMSD value of 0.065 Å.

- **Binding mode of Sorafenib**

Molecular docking of sorafenib was carried out to study its binding interactions and orientation. Such a binding pattern showed a docking score of -36.23 kcal/mol with ten hydrogen bonds and nine hydrophobic interactions. The carbonyl group of *N* methylpicolinamide head formed one hydrogen bond with the essential amino acid Cys919 located in the hinge region. Additionally, the pyridine ring formed three hydrophobic interactions with a hydrophobic pocket formed by amino acid residues, Leu1035, Phe918, and Ala866. The spacer phenyl ring is bound to another hydrophobic pocket consisting of Lys 868, Val916, and Val899. Furthermore, the urea moiety interacted with the protein through three hydrogen bonds at the DFG motif; where the two NH formed two hydrogen bonds with Glu885 while the urea carbonyl interacted with Asp1046. Finally, the terminal phenyl ring formed hydrophobic interactions with Leu1019, Ile1044, and His1026

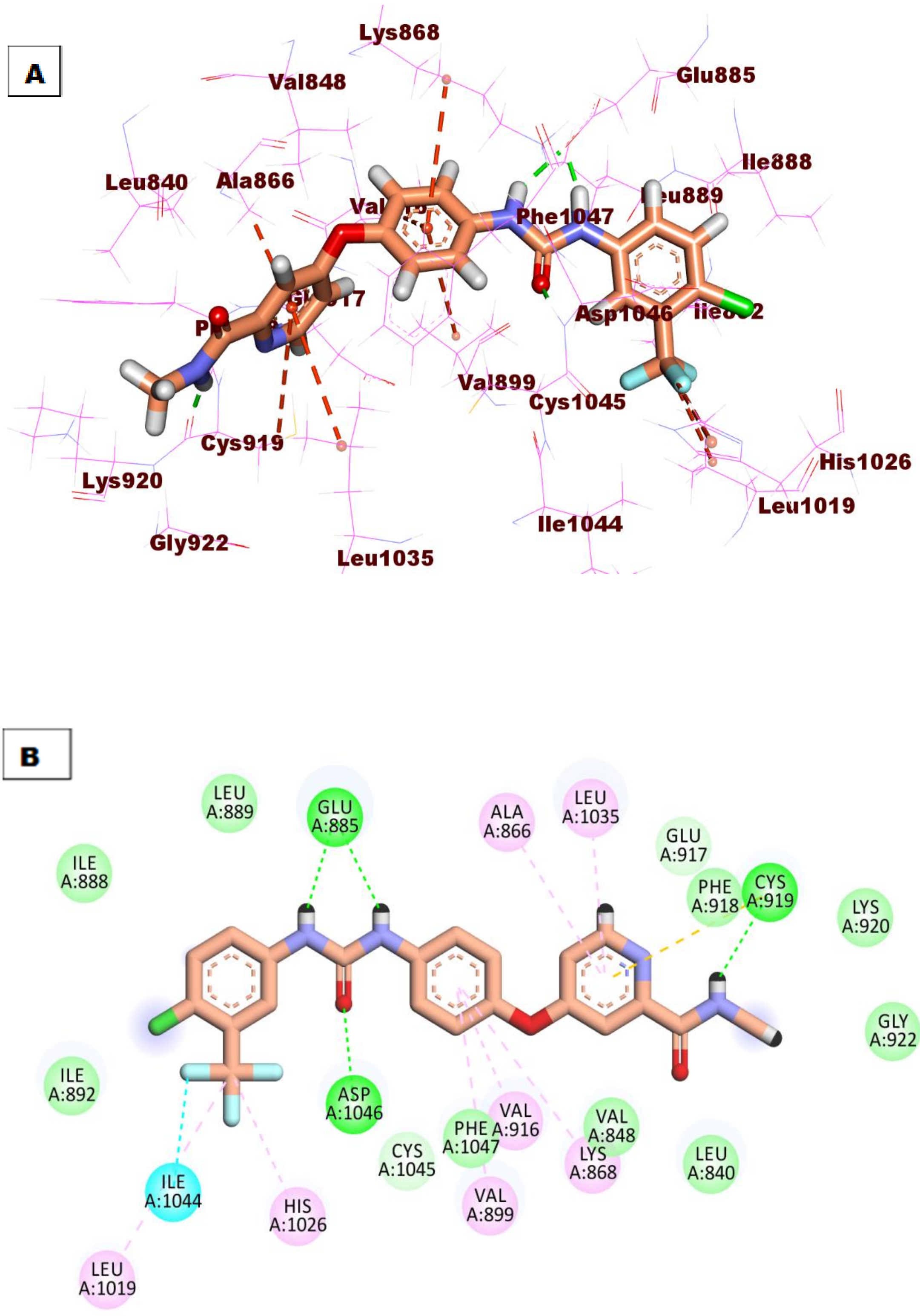

Fig. 8: **A**) 3D binding mode of sorafenib into VEGFR-2 active site, **B**) 2D binding mode of sorafenib into VEGFR-2 active site.

4.3.2. ADMET studies

ADMET descriptors (absorption, distribution, metabolism, excretion and toxicity) of the synthesized compounds were determined using Discovery studio 4.0. At first, the CHARMM force field was applied then the compounds were prepared and minimized according to the preparation of small molecule protocol. Then ADMET descriptors protocol was applied to carry out these studies.

4.3.3. Toxicity studies

The toxicity parameters of the synthesized compounds were calculated using Discovery studio 4.0. Sorafenib was used as a reference drug. At first, the CHARMM force field was applied then the compounds were prepared and minimized according to the preparation of small molecule protocol. Then different parameters were calculated from toxicity prediction (extensible) protocol.

4.3.4. Molecular dynamics simulation & MM/PBSA

The system was prepared using the web-based CHARMM-GUI[1-3] interface with the CHARMM36 force field[4]. All the simulations were done using the NAMD 2.13[5] package. The TIP3P explicit solvation model was used[6], and the periodic boundary conditions were set with a dimension of 82.65 Å, 82.36 Å, and 82.64 Å in x, y, and z, respectively. The parameters for the top docking results were generated using the CHARMM general force field[7] Afterward, the system was neutralized using ---- (Cl^-/Na^+) ions. The MD protocols involved minimization, equilibration, and production. a 2 *fs* time step of integration was chosen for all MD simulations, the equilibration was carried in the canonical (*NVT*) ensemble, while the isothermal–isobaric (*NPT*) ensemble was for the production. Through the 100 *ns* of MD production, the pressure was set at 1 atm using the Nose´–Hoover Langevin piston barostat[8, 9] with a Langevin piston decay of 0.05 *ps* and a period of 0.1 *ps*. The temperature was set at 298.15 K using the Langevin thermostat[10]. A distance cutoff of 12.0 Å was applied to short-range nonbonded interactions with a pair list distance of 16 Å, and Lennard Jones interactions were smoothly truncated at 8.0 Å. Long-range electrostatic interactions were treated using the particle-mesh Ewald (PME) method[11, 12], where a grid spacing of 1.0 Å was used for all simulation cells. All covalent bonds involving

hydrogen atoms were constrained using the SHAKE algorithm[13]. For consistency, we have applied the same protocol for all MD simulations.

Binding Energy Calculations

The one-average molecular mechanics generalized Born surface area (MM/GBSA)[14, 15] approach implemented in the MOLAICAL code[16] was used for the relative binding energy calculations, in which the ligand (L) binds to the protein receptor (R) to form the complex (RL),

$$\Delta G_{bind} = \Delta G_{RL} - \Delta G_R - \Delta G_L$$

which can be represented by contributions of different interactions,

$$\Delta G_{bind} = \Delta H - T\Delta S = \Delta E_{MM} + \Delta G_{Sol} - T\Delta S$$

where the changes in the gas phase molecular mechanics (ΔE_{MM}), solvation Gibbs energy (ΔG_{Sol}), and conformational entropy $(-T\Delta S)$ are determined as follows: ΔE_{MM} is the sum of the changes in the electrostatic energies ΔE_{ele}, the van der Waals energies ΔE_{vdW}, and the internal energies ΔE_{int} (bonded interactions); ΔG_{Sol} is the total of both the polar solvation (calculated using the generalized Born model) and the nonpolar solvation (calculated using the solvent-accessible surface area) and $-T\Delta S$ is calculated by the normal mode analysis. The solvent dielectric constant of 78.5 and the surface tension constant of 0.03012 kJ mol^{-1} Å^2 were used for MM/GBSA calculations.

[1] S. Jo, T. Kim, V.G. Iyer, W. Im, CHARMM-GUI: A web-based graphical user interface for CHARMM, Journal of computational chemistry 29(11) (2008) 1859-1865.

[2] B.R. Brooks, C.L. Brooks III, A.D. Mackerell Jr., L. Nilsson, R.J. Petrella, B. Roux, Y. Won, G. Archontis, C. Bartels, S. Boresch, A. Caflisch, L. Caves, Q. Cui, A.R. Dinner, M. Feig, S. Fischer, J. Gao, M. Hodoscek, W. Im, K. Kuczera, T. Lazaridis, J. Ma, V. Ovchinnikov, E. Paci, R.W. Pastor, C.B. Post, J.Z. Pu, M. Schaefer, B. Tidor, R.M. Venable, H.L. Woodcock, X. Wu, W. Yang, D.M. York, M. Karplus, CHARMM: The biomolecular simulation program, Journal of computational chemistry 30(10) (2009) 1545-1614.

[3] J. Lee, X. Cheng, J.M. Swails, M.S. Yeom, P.K. Eastman, J.A. Lemkul, S. Wei, J. Buckner, J.C. Jeong, Y. Qi, S. Jo, V.S. Pande, D.A. Case, C.L. Brooks, A.D. MacKerell, J.B. Klauda, W. Im, CHARMM-GUI Input Generator for NAMD, GROMACS, AMBER, OpenMM, and CHARMM/OpenMM Simulations Using the CHARMM36 Additive Force Field, Journal of chemical theory and computation 12(1) (2016) 405-413.

[4] R.B. Best, X. Zhu, J. Shim, P.E. Lopes, J. Mittal, M. Feig, A.D. Mackerell, Jr., Optimization of the additive CHARMM all-atom protein force field targeting improved sampling of the backbone phi, psi and side-chain chi(1) and chi(2) dihedral angles, Journal of chemical theory and computation 8(9) (2012) 3257-3273.

[5] J.C. Phillips, R. Braun, W. Wang, J. Gumbart, E. Tajkhorshid, E. Villa, C. Chipot, R.D. Skeel, L. Kale, K. Schulten, Scalable molecular dynamics with NAMD, Journal of computational chemistry 26(16) (2005) 1781-802.

[6] W.L. Jorgensen, J. Chandrasekhar, J.D. Madura, R.W. Impey, M.L. Klein, Comparison of simple potential functions for simulating liquid water, The Journal of Chemical Physics 79(2) (1983) 926-935.

[7] W. Yu, X. He, K. Vanommeslaeghe, A.D. MacKerell, Jr., Extension of the CHARMM General Force Field to sulfonyl-containing compounds and its utility in biomolecular simulations, Journal of computational chemistry 33(31) (2012) 2451-68.

[8] S. Nosé, M.L. Klein, Constant pressure molecular dynamics for molecular systems, Molecular Physics 50(5) (1983) 1055-1076.

[9] S. Nosé, A molecular dynamics method for simulations in the canonical ensemble, Molecular Physics 52(2) (1984) 255-268.

[10] G.S. Grest, K. Kremer, Molecular dynamics simulation for polymers in the presence of a heat bath, Physical review. A, General physics 33(5) (1986) 3628-3631.

[11] T. Darden, D. York, L. Pedersen, Particle mesh Ewald: AnN·log(N) method for Ewald sums in large systems, The Journal of Chemical Physics 98(12) (1993) 10089-10092.

[12] U. Essmann, L. Perera, M.L. Berkowitz, T. Darden, H. Lee, L.G. Pedersen, A smooth particle mesh Ewald method, The Journal of Chemical Physics 103(19) (1995) 8577-8593.

[13] J.-P. Ryckaert, G. Ciccotti, H.J.C. Berendsen, Numerical integration of the cartesian equations of motion of a system with constraints: molecular dynamics of n-alkanes, Journal of Computational Physics 23(3) (1977) 327-341.

[14] S. Genheden, U. Ryde, Comparison of end-point continuum-solvation methods for the calculation of protein-ligand binding free energies, Proteins 80(5) (2012) 1326-42.

[15] E. Wang, H. Sun, J. Wang, Z. Wang, H. Liu, J.Z.H. Zhang, T. Hou, End-Point Binding Free Energy Calculation with MM/PBSA and MM/GBSA: Strategies and Applications in Drug Design, Chemical reviews 119(16) (2019) 9478-9508.

[16] Q. Bai, S. Tan, T. Xu, H. Liu, J. Huang, X. Yao, MolAICal: a soft tool for 3D drug design of protein targets by artificial intelligence and classical algorithm, Briefings in bioinformatics 22(3) (2021) bbaa161.

5- ***In silico*** **toxicity data of final target compounds 6,7,10 and 11**

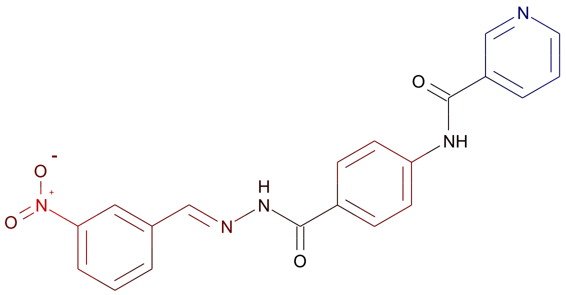

$C_{20}H_{15}N_5O_4$
Molecular Weight: 389.3642
ALogP: 2.211
Rotatable Bonds: 6
Acceptors: 6
Donors: 2

Model Prediction

Prediction: Mutagen
Probability: 0.901
Enrichment: 1.61
Bayesian Score: 5.03
Mahalanobis Distance: 7.41
Mahalanobis Distance p-value: 1

Prediction: Positive if the Bayesian score is above the estimated best cutoff value from minimizing the false positive and false negative rate.
Probability: The esimated probability that the sample is in the positive category. This assumes that the Bayesian score follows a normal distribution and is different from the prediction using a cutoff.
Enrichment: An estimate of enrichment, that is, the increased likelihood (versus random) of this sample being in the category.
Bayesian Score: The standard Laplacian-modified Bayesian score.
Mahalanobis Distance: The Mahalanobis distance (MD) is the distance to the center of the training data. The larger the MD, the less trustworthy the prediction.
Mahalanobis Distance p-value: The p-value gives the fraction of training data with an MD greater than or equal to the one for the given sample, assuming normally distributed data. The smaller the p-value, the less trustworthy the prediciton. For highly non-normal X properties (e.g., fingerprints), the MD p-value is wildly inaccurate.

Structural Similar Compounds

Name	119525-97-2	33372-39-3	Polythiazide
Structure	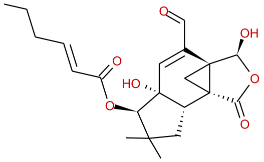	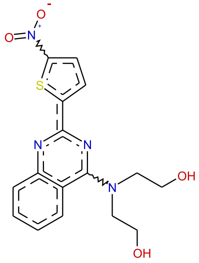	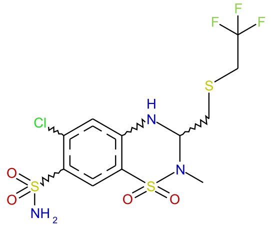
Actual Endpoint	Mutagen	Mutagen	Non-Mutagen
Predicted Endpoint	Mutagen	Mutagen	Non-Mutagen
Distance	0.588	0.590	0.601
Reference	Kazius et. al., J. Med. Chem. (2005) 48, 312-320	Kazius et. al., J. Med. Chem. (2005) 48, 312-320	Environ. Mol. Mut. 19(21):1992

Model Applicability

Unknown features are fingerprint features in the query molecule, but not found or appearing too infrequently in the training set.

1. All properties and OPS components are within expected ranges.

Feature Contribution

Top features for positive contribution				
**Fingerprint**	**Bit/Smiles**	**Feature Structure**	**Score**	**Mutagen in training set**
SCFP_12	555539852	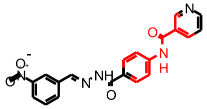 [*]:[cH]:[c](:[cH]:[*])C(=O)N[c]1:[cH]:[c H]:[*]:[cH]:[cH]:1	0.447	22 out of 24

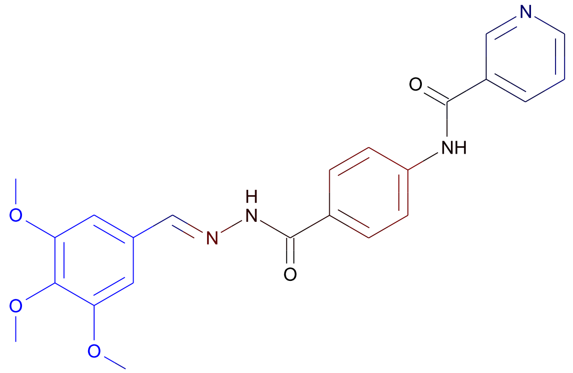

$C_{23}H_{22}N_4O_5$
Molecular Weight: 434.44457
ALogP: 2.268
Rotatable Bonds: 8
Acceptors: 7
Donors: 2

Structural Similar Compounds

Name	Delavirdine	94739-29-4	458-37-7
Structure	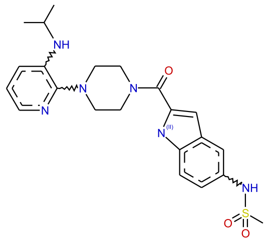	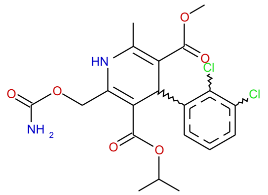	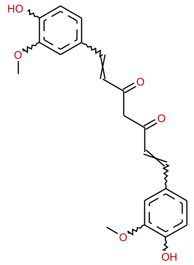
Actual Endpoint	Non-Mutagen	Non-Mutagen	Non-Mutagen
Predicted Endpoint	Non-Mutagen	Non-Mutagen	Non-Mutagen
Distance	0.571	0.579	0.607
Reference	Contrera, J.F., Matthews, E.J., Kruhlak, N.L., and Benz, R.D., Regulatory Toxicology and Pharmacology 2005, 313-323.	Kazius et. al., J. Med. Chem. (2005) 48, 312-320	Kazius et. al., J. Med. Chem. (2005) 48, 312-320

Model Prediction

Prediction: Non-Mutagen
Probability: 0.544
Enrichment: 0.974
Bayesian Score: -6.48
Mahalanobis Distance: 7.75
Mahalanobis Distance p-value: 0.997

Prediction: Positive if the Bayesian score is above the estimated best cutoff value from minimizing the false positive and false negative rate.
Probability: The esimated probability that the sample is in the positive category. This assumes that the Bayesian score follows a normal distribution and is different from the prediction using a cutoff.
Enrichment: An estimate of enrichment, that is, the increased likelihood (versus random) of this sample being in the category.
Bayesian Score: The standard Laplacian-modified Bayesian score.
Mahalanobis Distance: The Mahalanobis distance (MD) is the distance to the center of the training data. The larger the MD, the less trustworthy the prediction.
Mahalanobis Distance p-value: The p-value gives the fraction of training data with an MD greater than or equal to the one for the given sample, assuming normally distributed data. The smaller the p-value, the less trustworthy the prediciton. For highly non-normal X properties (e.g., fingerprints), the MD p-value is wildly inaccurate.

Model Applicability

Unknown features are fingerprint features in the query molecule, but not found or appearing too infrequently in the training set.

1. All properties and OPS components are within expected ranges.

Feature Contribution

Top features for positive contribution				
**Fingerprint**	**Bit/Smiles**	**Feature Structure**	**Score**	**Mutagen in training set**
SCFP_12	555539852	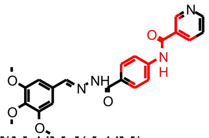 [*]:[cH]:[c](:[cH]:[*])C(=O)N[c]1:[cH]:[cH]:[*]:[cH]:[cH]:1	0.447	22 out of 24

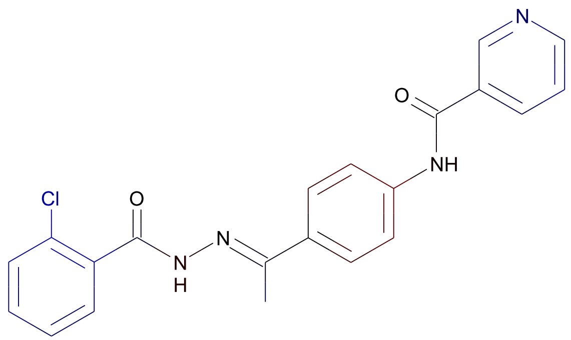

$C_{21}H_{17}ClN_4O_2$

Molecular Weight: 392.83827

ALogP: 2.962

Rotatable Bonds: 5

Acceptors: 4

Donors: 2

Structural Similar Compounds

Name	34433-31-3	IA 4 N-oxide	89784-39-4
Structure	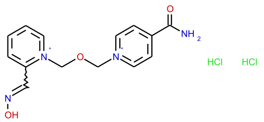	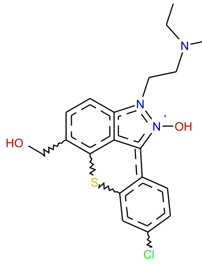	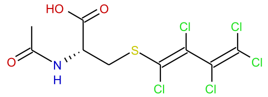
Actual Endpoint	Non-Mutagen	Mutagen	Mutagen
Predicted Endpoint	Non-Mutagen	Mutagen	Mutagen
Distance	0.535	0.537	0.558
Reference	Kazius et. al., J. Med. Chem. (2005) 48, 312-320	Contrera, J.F., Matthews, E.J., Kruhlak, N.L., and Benz, R.D., Regulatory Toxicology and Pharmacology 2005, 313-323.	Kazius et. al., J. Med. Chem. (2005) 48, 312-320

Model Prediction

Prediction: Non-Mutagen

Probability: 0.624

Enrichment: 1.12

Bayesian Score: -4.36

Mahalanobis Distance: 8.52

Mahalanobis Distance p-value: 0.95

Prediction: Positive if the Bayesian score is above the estimated best cutoff value from minimizing the false positive and false negative rate.
Probability: The esimated probability that the sample is in the positive category. This assumes that the Bayesian score follows a normal distribution and is different from the prediction using a cutoff.
Enrichment: An estimate of enrichment, that is, the increased likelihood (versus random) of this sample being in the category.
Bayesian Score: The standard Laplacian-modified Bayesian score.
Mahalanobis Distance: The Mahalanobis distance (MD) is the distance to the center of the training data. The larger the MD, the less trustworthy the prediction.
Mahalanobis Distance p-value: The p-value gives the fraction of training data with an MD greater than or equal to the one for the given sample, assuming normally distributed data. The smaller the p-value, the less trustworthy the prediciton. For highly non-normal X properties (e.g., fingerprints), the MD p-value is wildly inaccurate.

Model Applicability

Unknown features are fingerprint features in the query molecule, but not found or appearing too infrequently in the training set.

1. All properties and OPS components are within expected ranges.

Feature Contribution

Top features for positive contribution				
**Fingerprint**	**Bit/Smiles**	**Feature Structure**	**Score**	**Mutagen in training set**
SCFP_12	555539852	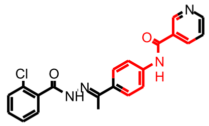 [*]:[cH]:[c](:[cH]:[*])C(=O)N[c]1:[cH]:[cH]:[*]:[cH]:[cH]:1	0.447	22 out of 24

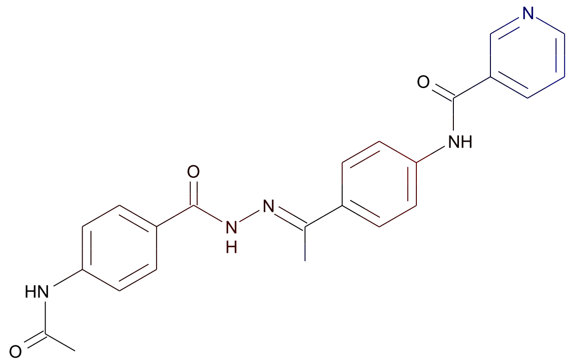

$C_{23}H_{21}N_5O_3$
Molecular Weight: 415.44453
ALogP: 1.418
Rotatable Bonds: 6
Acceptors: 5
Donors: 3

Model Prediction

Prediction: Non-Mutagen
Probability: 0.716
Enrichment: 1.28
Bayesian Score: -1.07
Mahalanobis Distance: 7.05
Mahalanobis Distance p-value: 1

Prediction: Positive if the Bayesian score is above the estimated best cutoff value from minimizing the false positive and false negative rate.
Probability: The esimated probability that the sample is in the positive category. This assumes that the Bayesian score follows a normal distribution and is different from the prediction using a cutoff.
Enrichment: An estimate of enrichment, that is, the increased likelihood (versus random) of this sample being in the category.
Bayesian Score: The standard Laplacian-modified Bayesian score.
Mahalanobis Distance: The Mahalanobis distance (MD) is the distance to the center of the training data. The larger the MD, the less trustworthy the prediction.
Mahalanobis Distance p-value: The p-value gives the fraction of training data with an MD greater than or equal to the one for the given sample, assuming normally distributed data. The smaller the p-value, the less trustworthy the prediciton. For highly non-normal X properties (e.g., fingerprints), the MD p-value is wildly inaccurate.

Structural Similar Compounds

Name	GLIPIZIDE	56211-40-6	67450-45-7
Structure	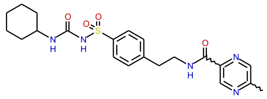	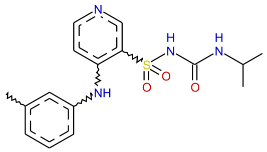	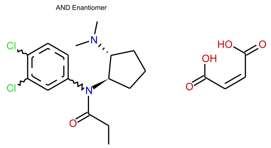
Actual Endpoint	Non-Mutagen	Non-Mutagen	Non-Mutagen
Predicted Endpoint	Non-Mutagen	Non-Mutagen	Non-Mutagen
Distance	0.587	0.594	0.613
Reference	PDR 1994	Kazius et. al., J. Med. Chem. (2005) 48, 312-320	Kazius et. al., J. Med. Chem. (2005) 48, 312-320

Model Applicability

Unknown features are fingerprint features in the query molecule, but not found or appearing too infrequently in the training set.

1. All properties and OPS components are within expected ranges.

Feature Contribution

Top features for positive contribution

Fingerprint	Bit/Smiles	Feature Structure	Score	Mutagen in training set
SCFP_12	555539852	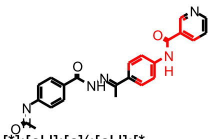 [*]:[cH]:[c](:[cH]:[*])C(=O)N[c]1:[cH]:[c H]:[*]:[cH]:[cH]:1	0.447	22 out of 24

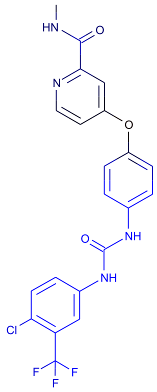

$C_{21}H_{16}ClF_3N_4O_3$
Molecular Weight: 464.82494
ALogP: 4.175
Rotatable Bonds: 6
Acceptors: 4
Donors: 3

Structural Similar Compounds

Name	GLYBURIDE	38914-96-4	93957-54-1
Structure	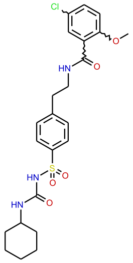	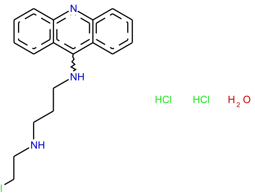	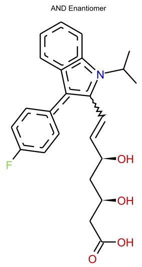
Actual Endpoint	Non-Mutagen	Mutagen	Non-Mutagen
Predicted Endpoint	Non-Mutagen	Mutagen	Non-Mutagen
Distance	0.590	0.592	0.600
Reference	PDR 1994	Kazius et. al., J. Med. Chem. (2005) 48, 312-320	US Environmental Protection Agency at http://www.epa.gov/NCCT/dsstox/sdf_isscan_external.html

Model Prediction

Prediction: Non-Mutagen
Probability: 0.0531
Enrichment: 0.0951
Bayesian Score: -19.7
Mahalanobis Distance: 13.1
Mahalanobis Distance p-value: 2.73e-006

Prediction: Positive if the Bayesian score is above the estimated best cutoff value from minimizing the false positive and false negative rate.
Probability: The esimated probability that the sample is in the positive category. This assumes that the Bayesian score follows a normal distribution and is different from the prediction using a cutoff.
Enrichment: An estimate of enrichment, that is, the increased likelihood (versus random) of this sample being in the category.
Bayesian Score: The standard Laplacian-modified Bayesian score.
Mahalanobis Distance: The Mahalanobis distance (MD) is the distance to the center of the training data. The larger the MD, the less trustworthy the prediction.
Mahalanobis Distance p-value: The p-value gives the fraction of training data with an MD greater than or equal to the one for the given sample, assuming normally distributed data. The smaller the p-value, the less trustworthy the prediciton. For highly non-normal X properties (e.g., fingerprints), the MD p-value is wildly inaccurate.

Model Applicability

Unknown features are fingerprint features in the query molecule, but not found or appearing too infrequently in the training set.

1. All properties and OPS components are within expected ranges.

Feature Contribution

Top features for positive contribution				
**Fingerprint**	**Bit/Smiles**	**Feature Structure**	**Score**	**Mutagen in training set**
SCFP_12	-347281112	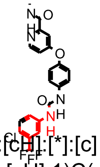 [*]N[c]1:[cH]:[*]:[c]([*]):[c](:[cH]:1)C([*])([*])[*]	0.337	18 out of 22

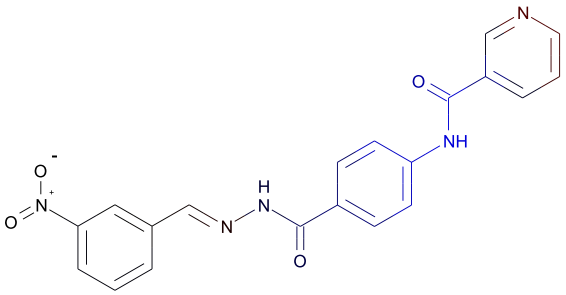

$C_{20}H_{15}N_5O_4$
Molecular Weight: 389.3642
ALogP: 2.211
Rotatable Bonds: 6
Acceptors: 6
Donors: 2

Model Prediction

Prediction: Mild

Probability: 0.652

Enrichment: 0.946

Bayesian Score: -4.31

Mahalanobis Distance: 4.55

Mahalanobis Distance p-value: 1

Prediction: Positive if the Bayesian score is above the estimated best cutoff value from minimizing the false positive and false negative rate.
Probability: The esimated probability that the sample is in the positive category. This assumes that the Bayesian score follows a normal distribution and is different from the prediction using a cutoff.
Enrichment: An estimate of enrichment, that is, the increased likelihood (versus random) of this sample being in the category.
Bayesian Score: The standard Laplacian-modified Bayesian score.
Mahalanobis Distance: The Mahalanobis distance (MD) is the distance to the center of the training data. The larger the MD, the less trustworthy the prediction.
Mahalanobis Distance p-value: The p-value gives the fraction of training data with an MD greater than or equal to the one for the given sample, assuming normally distributed data. The smaller the p-value, the less trustworthy the prediciton. For highly non-normal X properties (e.g., fingerprints), the MD p-value is wildly inaccurate.

Structural Similar Compounds

Name	ANTHRAQUINONE; 1-AMINO-4-HYDROXY-2-PHENOXY-	1-AMINO-4-BENZOYLAMINO-ANTHRAQUINONE	2-NAPHTHALENESULFONIC ACID;5-AMINO-6-ETHOXY-
Structure	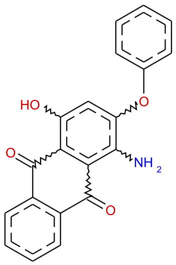	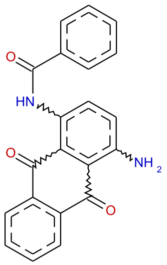	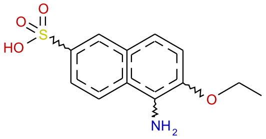
Actual Endpoint	Mild	Mild	Moderate_Severe
Predicted Endpoint	Mild	Mild	Mild
Distance	0.688	0.692	0.725
Reference	28ZPAK 239;72	28ZPAK-;124;72	28ZPAK-;191;72

Model Applicability

Unknown features are fingerprint features in the query molecule, but not found or appearing too infrequently in the training set.

1. All properties and OPS components are within expected ranges.
2. Unknown FCFP_2 feature: 581019816: [*]N\N=C\[*]
3. Unknown FCFP_2 feature: -828984032: [*][N+](=[*])[c](:[cH]:[*]):[cH]:[*]
4. Unknown FCFP_2 feature: -1338588315: [*]:[c](:[*])[N+](=O)[O-]
5. Unknown FCFP_2 feature: 1872392852: [*][N+](=O)[*]
6. Unknown FCFP_2 feature: 260476081: [*][N+](=[*])[O-]

Feature Contribution

Top features for positive contribution				
**Fingerprint**	**Bit/Smiles**	**Feature Structure**	**Score**	**Moderate_Severe in training set**

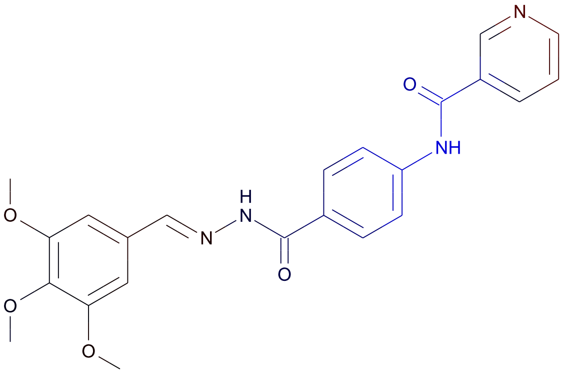

$C_{23}H_{22}N_4O_5$
Molecular Weight: 434.44457
ALogP: 2.268
Rotatable Bonds: 8
Acceptors: 7
Donors: 2

Model Prediction

Prediction: Mild
Probability: 0.632
Enrichment: 0.917
Bayesian Score: -4.59
Mahalanobis Distance: 6.42
Mahalanobis Distance p-value: 1

Prediction: Positive if the Bayesian score is above the estimated best cutoff value from minimizing the false positive and false negative rate.
Probability: The esimated probability that the sample is in the positive category. This assumes that the Bayesian score follows a normal distribution and is different from the prediction using a cutoff.
Enrichment: An estimate of enrichment, that is, the increased likelihood (versus random) of this sample being in the category.
Bayesian Score: The standard Laplacian-modified Bayesian score.
Mahalanobis Distance: The Mahalanobis distance (MD) is the distance to the center of the training data. The larger the MD, the less trustworthy the prediction.
Mahalanobis Distance p-value: The p-value gives the fraction of training data with an MD greater than or equal to the one for the given sample, assuming normally distributed data. The smaller the p-value, the less trustworthy the prediciton. For highly non-normal X properties (e.g., fingerprints), the MD p-value is wildly inaccurate.

Structural Similar Compounds

Name	COLCHICINE	ANTHRAQUINONE; 1-AMINO-4-HYDROXY-2-PHENOXY-	4;4'-DIAMINO-1;1'-DIANTHRIMIDE
Structure	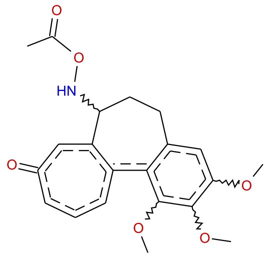	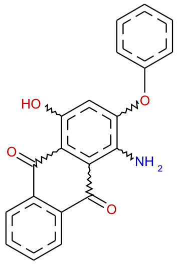	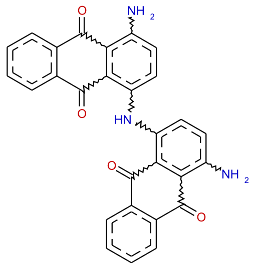
Actual Endpoint	Moderate_Severe	Mild	Mild
Predicted Endpoint	Moderate_Severe	Mild	Mild
Distance	0.705	0.799	0.804
Reference	AJOPAA 31;837;48	28ZPAK 239;72	28ZPAK-;125;72

Model Applicability

Unknown features are fingerprint features in the query molecule, but not found or appearing too infrequently in the training set.

1. All properties and OPS components are within expected ranges.
2. Unknown FCFP_2 feature: 581019816: [*]N\N=C\[*]

Feature Contribution

Top features for positive contribution				
Fingerprint	Bit/Smiles	Feature Structure	Score	Moderate_Severe in training set
FCFP_10	547884906	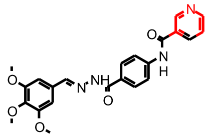 [*][c]1:[*]:[cH]:[cH]:n:[cH]:1	0.317	4 out of 4

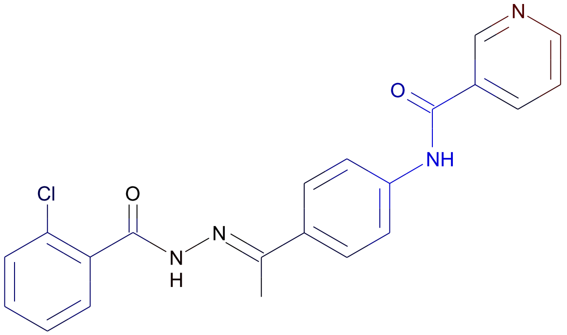

$C_{21}H_{17}ClN_4O_2$
Molecular Weight: 392.83827
ALogP: 2.962
Rotatable Bonds: 5
Acceptors: 4
Donors: 2

Model Prediction

Prediction: Mild

Probability: 0.568

Enrichment: 0.825

Bayesian Score: -5.4

Mahalanobis Distance: 6.76

Mahalanobis Distance p-value: 0.999

Prediction: Positive if the Bayesian score is above the estimated best cutoff value from minimizing the false positive and false negative rate.
Probability: The esimated probability that the sample is in the positive category. This assumes that the Bayesian score follows a normal distribution and is different from the prediction using a cutoff.
Enrichment: An estimate of enrichment, that is, the increased likelihood (versus random) of this sample being in the category.
Bayesian Score: The standard Laplacian-modified Bayesian score.
Mahalanobis Distance: The Mahalanobis distance (MD) is the distance to the center of the training data. The larger the MD, the less trustworthy the prediction.
Mahalanobis Distance p-value: The p-value gives the fraction of training data with an MD greater than or equal to the one for the given sample, assuming normally distributed data. The smaller the p-value, the less trustworthy the prediciton. For highly non-normal X properties (e.g., fingerprints), the MD p-value is wildly inaccurate.

Structural Similar Compounds

Name	1-AMINO-4-BENZOYLAMINO-ANTHRAQUINONE	5-NORBORNENE-2;3-DICARBOXYLIC ACID; 1;4;5;6;7;7-HEXACHLORO-	ANTHRAQUINONE; 1-AMINO-4-HYDROXY-2-PHENOXY-
Structure	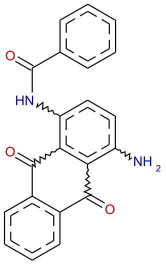	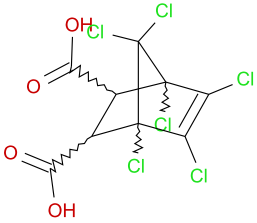	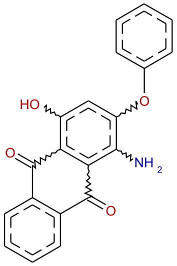
Actual Endpoint	Mild	Moderate_Severe	Mild
Predicted Endpoint	Mild	Moderate_Severe	Mild
Distance	0.582	0.592	0.659
Reference	28ZPAK-;124;72	28ZPAK-;92;72	28ZPAK 239;72

Model Applicability

Unknown features are fingerprint features in the query molecule, but not found or appearing too infrequently in the training set.

1. All properties and OPS components are within expected ranges.
2. Unknown FCFP_2 feature: 581019816: [*]N\N=C\[*]

Feature Contribution

Top features for positive contribution				
**Fingerprint**	**Bit/Smiles**	**Feature Structure**	**Score**	**Moderate_Severe in training set**
FCFP_10	547884906	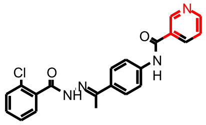 [*][c]1:[*]:[cH]:[cH]:n:[cH]:1	0.317	4 out of 4

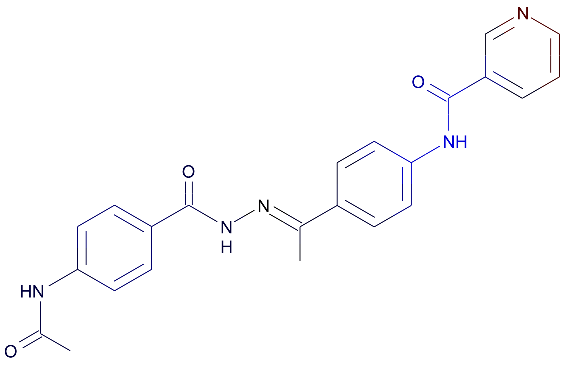

$C_{23}H_{21}N_5O_3$
Molecular Weight: 415.44453
ALogP: 1.418
Rotatable Bonds: 6
Acceptors: 5
Donors: 3

Structural Similar Compounds

Name	ANTHRAQUINONE; 1-((2-HYDROXYETHYL)AMINO)-4-(METHYLAMINO)-	p-Acetophenetidide; 3'-(bis(2-hydroxyethyl)amino)-	1-AMINO-4-BENZOYLAMINO-ANTHRAQUINONE
Structure	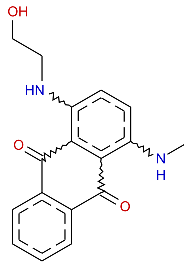	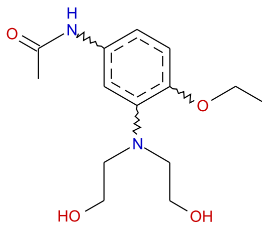	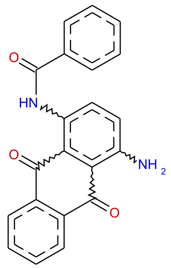
Actual Endpoint	Mild	Moderate_Severe	Mild
Predicted Endpoint	Mild	Moderate_Severe	Mild
Distance	0.673	0.696	0.772
Reference	28ZPAK 245;72	Prehled Prumyslove Toxikologie; Organicke Latky; Marhold; J. -;645;86	28ZPAK-;124;72

Model Prediction

Prediction: Mild
Probability: 0.544
Enrichment: 0.79
Bayesian Score: -5.68
Mahalanobis Distance: 7.75
Mahalanobis Distance p-value: 0.964

Prediction: Positive if the Bayesian score is above the estimated best cutoff value from minimizing the false positive and false negative rate.
Probability: The esimated probability that the sample is in the positive category. This assumes that the Bayesian score follows a normal distribution and is different from the prediction using a cutoff.
Enrichment: An estimate of enrichment, that is, the increased likelihood (versus random) of this sample being in the category.
Bayesian Score: The standard Laplacian-modified Bayesian score.
Mahalanobis Distance: The Mahalanobis distance (MD) is the distance to the center of the training data. The larger the MD, the less trustworthy the prediction.
Mahalanobis Distance p-value: The p-value gives the fraction of training data with an MD greater than or equal to the one for the given sample, assuming normally distributed data. The smaller the p-value, the less trustworthy the prediciton. For highly non-normal X properties (e.g., fingerprints), the MD p-value is wildly inaccurate.

Model Applicability

Unknown features are fingerprint features in the query molecule, but not found or appearing too infrequently in the training set.

1. All properties and OPS components are within expected ranges.
2. Unknown FCFP_2 feature: 581019816: [*]N\N=C\[*]

Feature Contribution

Top features for positive contribution				
**Fingerprint**	**Bit/Smiles**	**Feature Structure**	**Score**	**Moderate_Severe in training set**
FCFP_10	547884906	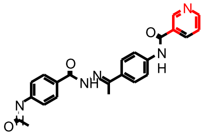 [*][c]1:[*]:[cH]:[cH]:n:[cH]:1	0.317	4 out of 4

Sorafenib

TOPKAT_Ocular_Irritancy_Mild_vs_Moderate_Severe

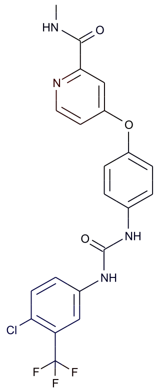

$C_{21}H_{16}ClF_3N_4O_3$
Molecular Weight: 464.82494
ALogP: 4.175
Rotatable Bonds: 6
Acceptors: 4
Donors: 3

Structural Similar Compounds

Name	4;4'-DIAMINO-1;1'-DIANTHRIMIDE	5-NORBORNENE-2;3-DICARBOXYLIC ACID; 1;4;5;6;7;7-HEXACHLORO-	METHANE;TRIS(4-AMINOPHENYL)-
Structure	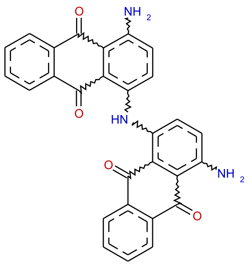	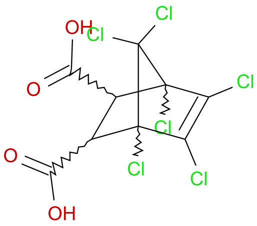	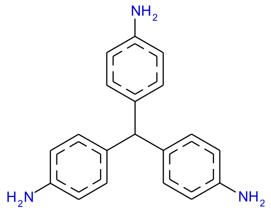
Actual Endpoint	Mild	Moderate_Severe	Moderate_Severe
Predicted Endpoint	Mild	Moderate_Severe	Moderate_Severe
Distance	0.799	0.816	0.827
Reference	28ZPAK-;125;72	28ZPAK-;92;72	28ZPAK-;73;72

Model Prediction

Prediction: Mild
Probability: 0.776
Enrichment: 1.13
Bayesian Score: -1.8
Mahalanobis Distance: 8.95
Mahalanobis Distance p-value: 0.537

Prediction: Positive if the Bayesian score is above the estimated best cutoff value from minimizing the false positive and false negative rate.
Probability: The esimated probability that the sample is in the positive category. This assumes that the Bayesian score follows a normal distribution and is different from the prediction using a cutoff.
Enrichment: An estimate of enrichment, that is, the increased likelihood (versus random) of this sample being in the category.
Bayesian Score: The standard Laplacian-modified Bayesian score.
Mahalanobis Distance: The Mahalanobis distance (MD) is the distance to the center of the training data. The larger the MD, the less trustworthy the prediction.
Mahalanobis Distance p-value: The p-value gives the fraction of training data with an MD greater than or equal to the one for the given sample, assuming normally distributed data. The smaller the p-value, the less trustworthy the prediciton. For highly non-normal X properties (e.g., fingerprints), the MD p-value is wildly inaccurate.

Model Applicability

Unknown features are fingerprint features in the query molecule, but not found or appearing too infrequently in the training set.

1. All properties and OPS components are within expected ranges.

Feature Contribution

Top features for positive contribution

Fingerprint	Bit/Smiles	Feature Structure	Score	Moderate_Severe in training set
FCFP_10	-1695756380	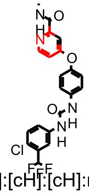 [*]1:[cH]:[cH]:[cH]:n:[cH]:1	0.285	10 out of 11

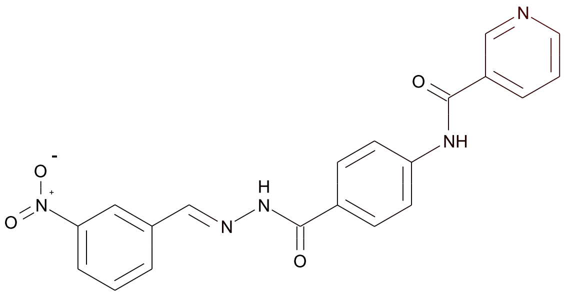

$C_{20}H_{15}N_5O_4$

Molecular Weight: 389.3642

ALogP: 2.211

Rotatable Bonds: 6

Acceptors: 6

Donors: 2

Structural Similar Compounds

Name	ANTHRAQUINONE; 1-AMINO-4-HYDROXY-2-PHENOXY-	1-AMINO-4-BENZOYLAMINO-ANTHRAQUINONE	2;2';-Dihydroxy-4;4'-dimethoxybenzophenone
Structure	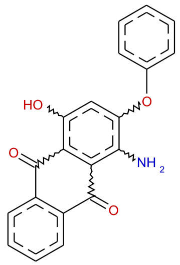	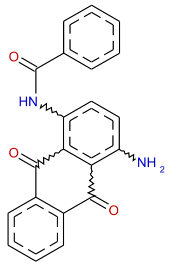	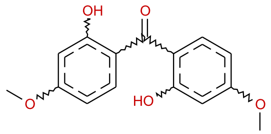
Actual Endpoint	Irritant	Irritant	Non-Irritant
Predicted Endpoint	Irritant	Irritant	Non-Irritant
Distance	0.674	0.686	0.712
Reference	28ZPAK 239;72	28ZPAK-;124;72	J. Am. Coll. Toxicol. 2(5):35;1983

Model Prediction

Prediction: Irritant

Probability: 1

Enrichment: 1.18

Bayesian Score: 2.25

Mahalanobis Distance: 3.98

Mahalanobis Distance p-value: 1

Prediction: Positive if the Bayesian score is above the estimated best cutoff value from minimizing the false positive and false negative rate.
Probability: The esimated probability that the sample is in the positive category. This assumes that the Bayesian score follows a normal distribution and is different from the prediction using a cutoff.
Enrichment: An estimate of enrichment, that is, the increased likelihood (versus random) of this sample being in the category.
Bayesian Score: The standard Laplacian-modified Bayesian score.
Mahalanobis Distance: The Mahalanobis distance (MD) is the distance to the center of the training data. The larger the MD, the less trustworthy the prediction.
Mahalanobis Distance p-value: The p-value gives the fraction of training data with an MD greater than or equal to the one for the given sample, assuming normally distributed data. The smaller the p-value, the less trustworthy the prediciton. For highly non-normal X properties (e.g., fingerprints), the MD p-value is wildly inaccurate.

Model Applicability

Unknown features are fingerprint features in the query molecule, but not found or appearing too infrequently in the training set.

1. All properties and OPS components are within expected ranges.
2. Unknown FCFP_2 feature: 581019816: [*]N\N=C\[*]
3. Unknown FCFP_2 feature: -828984032: [*][N+](=[*])[c](:[cH]:[*]):[cH]:[*]
4. Unknown FCFP_2 feature: -1338588315: [*]:[c](:[*])[N+](=O)[O-]
5. Unknown FCFP_2 feature: 1872392852: [*][N+](=O)[*]
6. Unknown FCFP_2 feature: 260476081: [*][N+](=[*])[O-]

Feature Contribution

Top features for positive contribution				
**Fingerprint**	**Bit/Smiles**	**Feature Structure**	**Score**	**Irritant in training set**

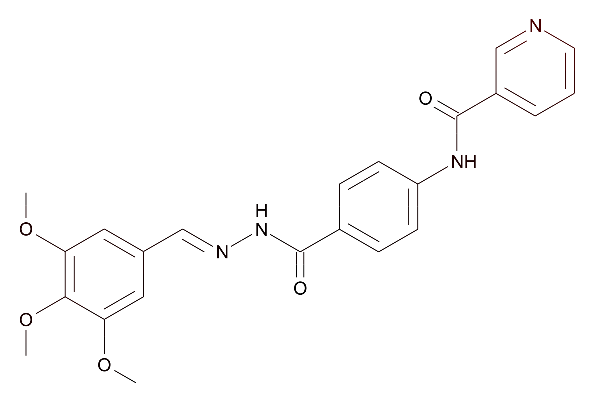

$C_{23}H_{22}N_4O_5$
Molecular Weight: 434.44457
ALogP: 2.268
Rotatable Bonds: 8
Acceptors: 7
Donors: 2

Model Prediction

Prediction: Irritant
Probability: 1
Enrichment: 1.18
Bayesian Score: 2.7
Mahalanobis Distance: 5.93
Mahalanobis Distance p-value: 1

Prediction: Positive if the Bayesian score is above the estimated best cutoff value from minimizing the false positive and false negative rate.
Probability: The esimated probability that the sample is in the positive category. This assumes that the Bayesian score follows a normal distribution and is different from the prediction using a cutoff.
Enrichment: An estimate of enrichment, that is, the increased likelihood (versus random) of this sample being in the category.
Bayesian Score: The standard Laplacian-modified Bayesian score.
Mahalanobis Distance: The Mahalanobis distance (MD) is the distance to the center of the training data. The larger the MD, the less trustworthy the prediction.
Mahalanobis Distance p-value: The p-value gives the fraction of training data with an MD greater than or equal to the one for the given sample, assuming normally distributed data. The smaller the p-value, the less trustworthy the prediciton. For highly non-normal X properties (e.g., fingerprints), the MD p-value is wildly inaccurate.

Structural Similar Compounds

Name	COLCHICINE	ANTHRAQUINONE; 1-AMINO-4-HYDROXY-2-PHENOXY-	4;4'-DIAMINO-1;1'-DIANTHRIMIDE
Structure	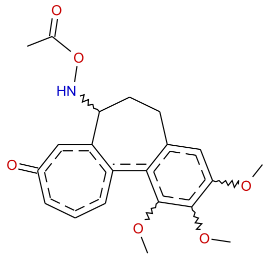	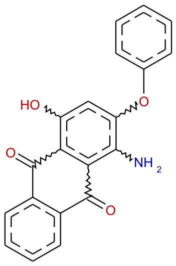	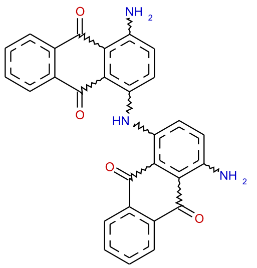
Actual Endpoint	Irritant	Irritant	Irritant
Predicted Endpoint	Irritant	Irritant	Irritant
Distance	0.707	0.781	0.791
Reference	AJOPAA 31;837;48	28ZPAK 239;72	28ZPAK-;125;72

Model Applicability

Unknown features are fingerprint features in the query molecule, but not found or appearing too infrequently in the training set.

1. All properties and OPS components are within expected ranges.
2. Unknown FCFP_2 feature: 581019816: [*]N\N=C\[*]

Feature Contribution

Top features for positive contribution

Fingerprint	Bit/Smiles	Feature Structure	Score	Irritant in training set
FCFP_12	1747237384	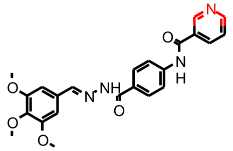 [*]:[cH]:n:[cH]:[*]	0.208	44 out of 44

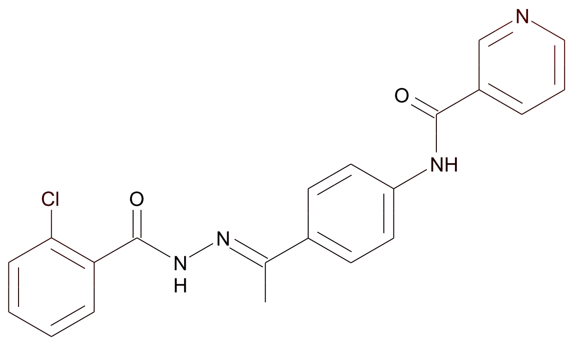

$C_{21}H_{17}ClN_4O_2$
Molecular Weight: 392.83827
ALogP: 2.962
Rotatable Bonds: 5
Acceptors: 4
Donors: 2

Structural Similar Compounds

Name	1-AMINO-4-BENZOYLAMINO-ANTHRAQUINONE	5-NORBORNENE-2;3-DICARBOXYLIC ACID; 1;4;5;6;7;7-HEXACHLORO-	ANTHRAQUINONE; 1-AMINO-4-HYDROXY-2-PHENOXY-
Structure	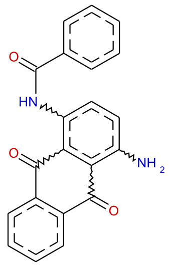	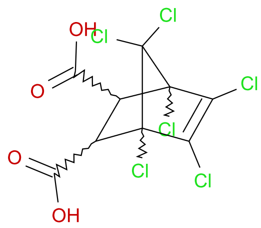	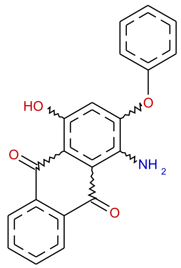
Actual Endpoint	Irritant	Irritant	Irritant
Predicted Endpoint	Irritant	Irritant	Irritant
Distance	0.581	0.585	0.654
Reference	28ZPAK-;124;72	28ZPAK-;92;72	28ZPAK 239;72

Model Prediction

Prediction: Irritant
Probability: 1
Enrichment: 1.18
Bayesian Score: 2.84
Mahalanobis Distance: 5.52
Mahalanobis Distance p-value: 1

Prediction: Positive if the Bayesian score is above the estimated best cutoff value from minimizing the false positive and false negative rate.
Probability: The esimated probability that the sample is in the positive category. This assumes that the Bayesian score follows a normal distribution and is different from the prediction using a cutoff.
Enrichment: An estimate of enrichment, that is, the increased likelihood (versus random) of this sample being in the category.
Bayesian Score: The standard Laplacian-modified Bayesian score.
Mahalanobis Distance: The Mahalanobis distance (MD) is the distance to the center of the training data. The larger the MD, the less trustworthy the prediction.
Mahalanobis Distance p-value: The p-value gives the fraction of training data with an MD greater than or equal to the one for the given sample, assuming normally distributed data. The smaller the p-value, the less trustworthy the prediciton. For highly non-normal X properties (e.g., fingerprints), the MD p-value is wildly inaccurate.

Model Applicability

Unknown features are fingerprint features in the query molecule, but not found or appearing too infrequently in the training set.

1. All properties and OPS components are within expected ranges.
2. Unknown FCFP_2 feature: 581019816: [*]N\N=C\[*]

Feature Contribution

Top features for positive contribution				
**Fingerprint**	**Bit/Smiles**	**Feature Structure**	**Score**	**Irritant in training set**
FCFP_12	1747237384	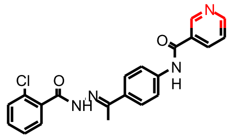 [*]:[cH]:n:[cH]:[*]	0.208	44 out of 44

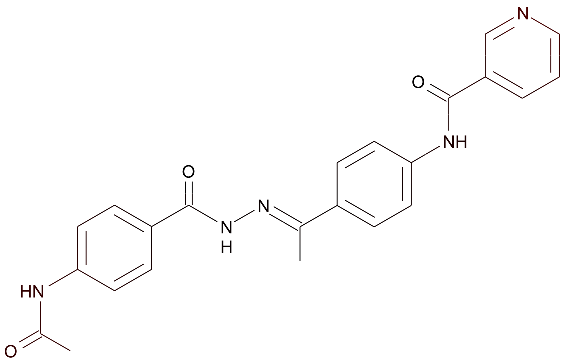

$C_{23}H_{21}N_5O_3$
Molecular Weight: 415.44453
ALogP: 1.418
Rotatable Bonds: 6
Acceptors: 5
Donors: 3

Structural Similar Compounds

Name	ANTHRAQUINONE; 1-((2-HYDROXYETHYL)AMINO)-4-(METHYLAMINO)-	p-Acetophenetidide; 3'-(bis(2-hydroxyethyl)amino)-	Disperse Black 9
Structure	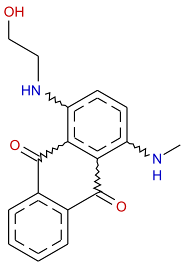	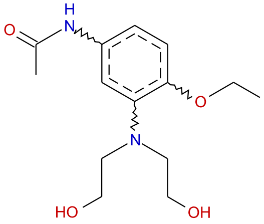	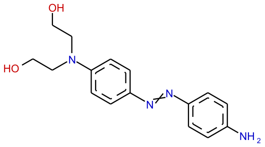
Actual Endpoint	Irritant	Irritant	Non-Irritant
Predicted Endpoint	Irritant	Irritant	Non-Irritant
Distance	0.664	0.684	0.710
Reference	28ZPAK 245;72	Prehled Prumyslove Toxikologie; Organicke Latky; Marhold; J. -;645;86	J. Am. Coll. Toxicol. 5(3):205;1986

Model Prediction

Prediction: Irritant
Probability: 1
Enrichment: 1.18
Bayesian Score: 2.95
Mahalanobis Distance: 6.96
Mahalanobis Distance p-value: 0.999

Prediction: Positive if the Bayesian score is above the estimated best cutoff value from minimizing the false positive and false negative rate.
Probability: The esimated probability that the sample is in the positive category. This assumes that the Bayesian score follows a normal distribution and is different from the prediction using a cutoff.
Enrichment: An estimate of enrichment, that is, the increased likelihood (versus random) of this sample being in the category.
Bayesian Score: The standard Laplacian-modified Bayesian score.
Mahalanobis Distance: The Mahalanobis distance (MD) is the distance to the center of the training data. The larger the MD, the less trustworthy the prediction.
Mahalanobis Distance p-value: The p-value gives the fraction of training data with an MD greater than or equal to the one for the given sample, assuming normally distributed data. The smaller the p-value, the less trustworthy the prediciton. For highly non-normal X properties (e.g., fingerprints), the MD p-value is wildly inaccurate.

Model Applicability

Unknown features are fingerprint features in the query molecule, but not found or appearing too infrequently in the training set.

1. All properties and OPS components are within expected ranges.
2. Unknown FCFP_2 feature: 581019816: [*]N\N=C\[*]

Feature Contribution

Top features for positive contribution				
**Fingerprint**	**Bit/Smiles**	**Feature Structure**	**Score**	**Irritant in training set**
FCFP_12	1747237384	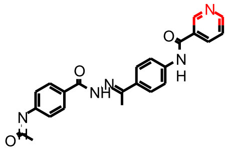 [*]:[cH]:n:[cH]:[*]	0.208	44 out of 44

Sorafenib

TOPKAT_Ocular_Irritancy_None_vs_Irritant

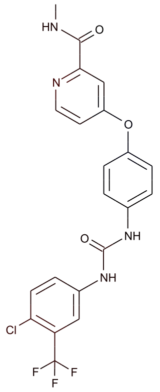

$C_{21}H_{16}ClF_3N_4O_3$
Molecular Weight: 464.82494
ALogP: 4.175
Rotatable Bonds: 6
Acceptors: 4
Donors: 3

Model Prediction

Prediction: Irritant
Probability: 1
Enrichment: 1.18
Bayesian Score: 3.04
Mahalanobis Distance: 6.28
Mahalanobis Distance p-value: 1

Prediction: Positive if the Bayesian score is above the estimated best cutoff value from minimizing the false positive and false negative rate.
Probability: The esimated probability that the sample is in the positive category. This assumes that the Bayesian score follows a normal distribution and is different from the prediction using a cutoff.
Enrichment: An estimate of enrichment, that is, the increased likelihood (versus random) of this sample being in the category.
Bayesian Score: The standard Laplacian-modified Bayesian score.
Mahalanobis Distance: The Mahalanobis distance (MD) is the distance to the center of the training data. The larger the MD, the less trustworthy the prediction.
Mahalanobis Distance p-value: The p-value gives the fraction of training data with an MD greater than or equal to the one for the given sample, assuming normally distributed data. The smaller the p-value, the less trustworthy the prediciton. For highly non-normal X properties (e.g., fingerprints), the MD p-value is wildly inaccurate.

Structural Similar Compounds

Name	BENZANILIDE;2';2'''-DITHIOBIS-	4;4'-DIAMINO-1;1'-DIANTHRIMIDE	5-NORBORNENE-2;3-DICARBOXYLIC ACID; 1;4;5;6;7;7-HEXACHLORO-
Structure	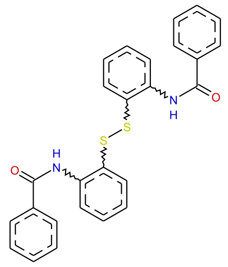	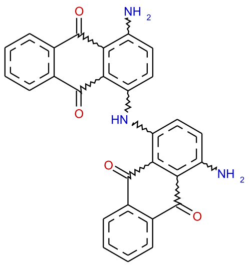	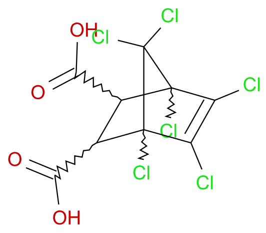
Actual Endpoint	Non-Irritant	Irritant	Irritant
Predicted Endpoint	Non-Irritant	Irritant	Irritant
Distance	0.743	0.791	0.801
Reference	28ZPAK-;173;72	28ZPAK-;125;72	28ZPAK-;92;72

Model Applicability

Unknown features are fingerprint features in the query molecule, but not found or appearing too infrequently in the training set.

1. All properties and OPS components are within expected ranges.

Feature Contribution

Top features for positive contribution

Fingerprint	Bit/Smiles	Feature Structure	Score	Irritant in training set
FCFP_12	1747237384	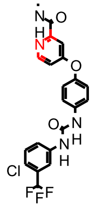 [*]:[cH]:n:[cH]:[*]	0.208	44 out of 44

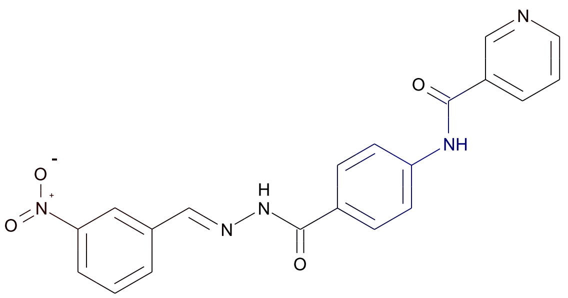

$C_{20}H_{15}N_5O_4$
Molecular Weight: 389.3642
ALogP: 2.211
Rotatable Bonds: 6
Acceptors: 6
Donors: 2

Model Prediction

Prediction: Non-Irritant

Probability: 0.955

Enrichment: 1.04

Bayesian Score: -1.73

Mahalanobis Distance: 5.53

Mahalanobis Distance p-value: 1

Prediction: Positive if the Bayesian score is above the estimated best cutoff value from minimizing the false positive and false negative rate.
Probability: The esimated probability that the sample is in the positive category. This assumes that the Bayesian score follows a normal distribution and is different from the prediction using a cutoff.
Enrichment: An estimate of enrichment, that is, the increased likelihood (versus random) of this sample being in the category.
Bayesian Score: The standard Laplacian-modified Bayesian score.
Mahalanobis Distance: The Mahalanobis distance (MD) is the distance to the center of the training data. The larger the MD, the less trustworthy the prediction.
Mahalanobis Distance p-value: The p-value gives the fraction of training data with an MD greater than or equal to the one for the given sample, assuming normally distributed data. The smaller the p-value, the less trustworthy the prediciton. For highly non-normal X properties (e.g., fingerprints), the MD p-value is wildly inaccurate.

Structural Similar Compounds

Name	2-Anthracenesulfonic acid, 1-amino-9,10-dihydro-9,10-dioxo-4-(2,4,6 -trimethylanilino)-, monosodium salt	Benzenesulfonamide, 4-amino-N-(5,6-dimethoxy-4-pyrimidinyl)-	Benzenesulfonic acid, 2-anilino-5-nitro-
Structure	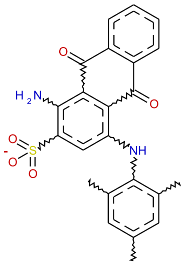	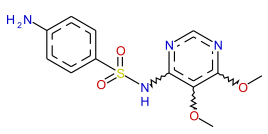	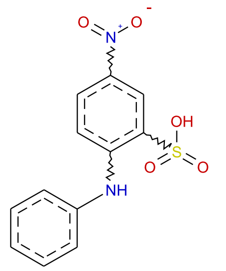
Actual Endpoint	Irritant	Irritant	Irritant
Predicted Endpoint	Non-Irritant	Non-Irritant	Non-Irritant
Distance	0.661	0.704	0.725
Reference	85JCAE "Prehled Prumyslove Toxikologie; Organicke Latky," Marhold, J., Prague , Czechoslovakia, Avicenum, 1986 Volume(issue)/page/year: -,1327,1986	FCTXAV 14,307,76	85JCAE "Prehled Prumyslove Toxikologie; Organicke Latky," Marhold, J., Prague , Czechoslovakia, Avicenum, 1986 Volume(issue)/page/year: -,1061,1986

Model Applicability

Unknown features are fingerprint features in the query molecule, but not found or appearing too infrequently in the training set.

1. All properties and OPS components are within expected ranges.

Feature Contribution

Top features for positive contribution

Fingerprint	Bit/Smiles	Feature Structure	Score	Irritant in training set

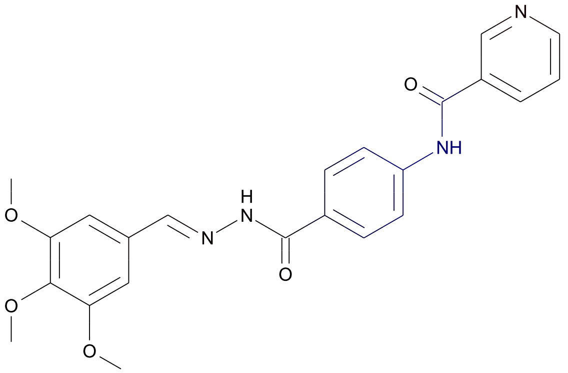

$C_{23}H_{22}N_4O_5$

Molecular Weight: 434.44457

ALogP: 2.268

Rotatable Bonds: 8

Acceptors: 7

Donors: 2

Model Prediction

Prediction: Non-Irritant

Probability: 0.924

Enrichment: 1

Bayesian Score: -2.44

Mahalanobis Distance: 6.52

Mahalanobis Distance p-value: 1

Prediction: Positive if the Bayesian score is above the estimated best cutoff value from minimizing the false positive and false negative rate.
Probability: The esimated probability that the sample is in the positive category. This assumes that the Bayesian score follows a normal distribution and is different from the prediction using a cutoff.
Enrichment: An estimate of enrichment, that is, the increased likelihood (versus random) of this sample being in the category.
Bayesian Score: The standard Laplacian-modified Bayesian score.
Mahalanobis Distance: The Mahalanobis distance (MD) is the distance to the center of the training data. The larger the MD, the less trustworthy the prediction.
Mahalanobis Distance p-value: The p-value gives the fraction of training data with an MD greater than or equal to the one for the given sample, assuming normally distributed data. The smaller the p-value, the less trustworthy the prediciton. For highly non-normal X properties (e.g., fingerprints), the MD p-value is wildly inaccurate.

Structural Similar Compounds

Name	Pregna-1,4-diene-3,20-dione, 21-(acetyloxy)-11-hydroxy-6-methyl-17- (1-oxopropoxy)-, (6-alpha,11-beta)-	2-Anthracenesulfonic acid, 1-amino-9,10-dihydro-9,10-dioxo-4-(2,4,6 -trimethylanilino)-, monosodium salt	Benzenesulfonamide, 4-amino-N-(5,6-dimethoxy-4-pyrimidinyl)-
Structure	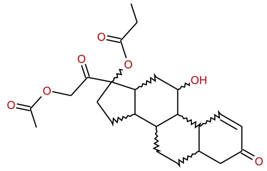	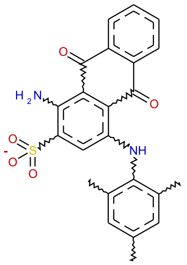	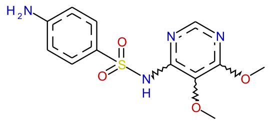
Actual Endpoint	Irritant	Irritant	Irritant
Predicted Endpoint	Irritant	Non-Irritant	Non-Irritant
Distance	0.699	0.756	0.823
Reference	YACHDS Yakuri to Chiryo. Pharmacology and Therapeutics. (Raifu Saiensu Shup pan K.K., 2-5-13, Yaesu, Chuo-ku, Tokyo 104, Japan) V.1-1972- Volume(issue) /page/year: 19,3103,1991	85JCAE "Prehled Prumyslove Toxikologie; Organicke Latky," Marhold, J., Prague , Czechoslovakia, Avicenum, 1986 Volume(issue)/page/year: -,1327,1986	FCTXAV 14,307,76

Model Applicability

Unknown features are fingerprint features in the query molecule, but not found or appearing too infrequently in the training set.

1. All properties and OPS components are within expected ranges.

Feature Contribution

Top features for positive contribution

Fingerprint	Bit/Smiles	Feature Structure	Score	Irritant in training set

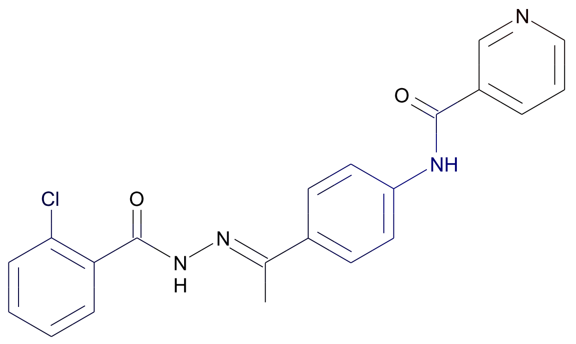

$C_{21}H_{17}ClN_4O_2$
Molecular Weight: 392.83827
ALogP: 2.962
Rotatable Bonds: 5
Acceptors: 4
Donors: 2

Model Prediction

Prediction: Non-Irritant
Probability: 0.6
Enrichment: 0.652
Bayesian Score: -4.27
Mahalanobis Distance: 6.85
Mahalanobis Distance p-value: 0.998

Prediction: Positive if the Bayesian score is above the estimated best cutoff value from minimizing the false positive and false negative rate.
Probability: The esimated probability that the sample is in the positive category. This assumes that the Bayesian score follows a normal distribution and is different from the prediction using a cutoff.
Enrichment: An estimate of enrichment, that is, the increased likelihood (versus random) of this sample being in the category.
Bayesian Score: The standard Laplacian-modified Bayesian score.
Mahalanobis Distance: The Mahalanobis distance (MD) is the distance to the center of the training data. The larger the MD, the less trustworthy the prediction.
Mahalanobis Distance p-value: The p-value gives the fraction of training data with an MD greater than or equal to the one for the given sample, assuming normally distributed data. The smaller the p-value, the less trustworthy the prediciton. For highly non-normal X properties (e.g., fingerprints), the MD p-value is wildly inaccurate.

Structural Similar Compounds

Name	5-Norbornene-2,3-dicarboxylic acid, 1,4,5,6,7,7-hexachloro-	1-Amino-2-bromo-4-hydroxyanthraquinone	1-Piperazineacetic acid, 4-(2-hydroxyethyl)-alpha-phenyl-, 2,6-xyly l ester, monohydrochloride
Structure	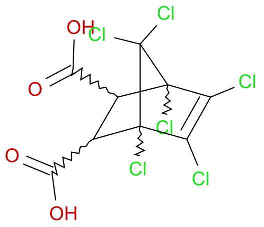	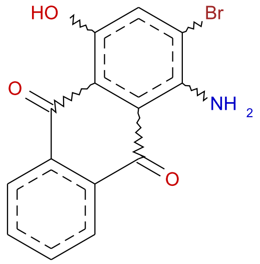	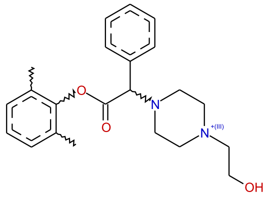
Actual Endpoint	Irritant	Non-Irritant	Irritant
Predicted Endpoint	Irritant	Non-Irritant	Irritant
Distance	0.637	0.750	0.764
Reference	85JCAE "Prehled Prumyslove Toxikologie; Organicke Latky," Marhold, J., Prague , Czechoslovakia, Avicenum, 1986 Volume(issue)/page/year: -,581,1986	28ZPAK -,83,72	BCFAAI Bollettino Chimico Farmaceutico. (Societa Editoriale Farmaceutica, Vi a Ausonio 12, 20123 Milan, Italy) V.33- 1894- Volume(issue)/page/year: 107,3 10,1968

Model Applicability

Unknown features are fingerprint features in the query molecule, but not found or appearing too infrequently in the training set.

1. All properties and OPS components are within expected ranges.

Feature Contribution

Top features for positive contribution				
Fingerprint	Bit/Smiles	Feature Structure	Score	Irritant in training set

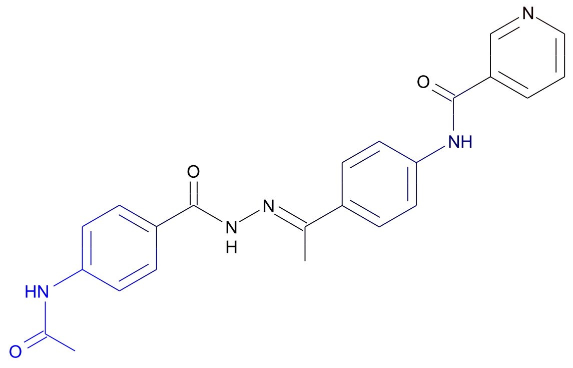

$C_{23}H_{21}N_5O_3$

Molecular Weight: 415.44453

ALogP: 1.418

Rotatable Bonds: 6

Acceptors: 5

Donors: 3

Model Prediction

Prediction: Non-Irritant

Probability: 0.000125

Enrichment: 0.000136

Bayesian Score: -8.87

Mahalanobis Distance: 7.25

Mahalanobis Distance p-value: 0.989

Prediction: Positive if the Bayesian score is above the estimated best cutoff value from minimizing the false positive and false negative rate.
Probability: The esimated probability that the sample is in the positive category. This assumes that the Bayesian score follows a normal distribution and is different from the prediction using a cutoff.
Enrichment: An estimate of enrichment, that is, the increased likelihood (versus random) of this sample being in the category.
Bayesian Score: The standard Laplacian-modified Bayesian score.
Mahalanobis Distance: The Mahalanobis distance (MD) is the distance to the center of the training data. The larger the MD, the less trustworthy the prediction.
Mahalanobis Distance p-value: The p-value gives the fraction of training data with an MD greater than or equal to the one for the given sample, assuming normally distributed data. The smaller the p-value, the less trustworthy the prediciton. For highly non-normal X properties (e.g., fingerprints), the MD p-value is wildly inaccurate.

Structural Similar Compounds

Name	p-Acetophenetidide, 3'-(bis(2-hydroxyethyl)amino)-	2-Anthracenesulfonic acid, 1-amino-9,10-dihydro-9,10-dioxo-4-(2,4,6 -trimethylanilino)-, monosodium salt	5-Norbornene-2,3-dicarboxylic acid, 1,4,5,6,7,7-hexachloro-
Structure	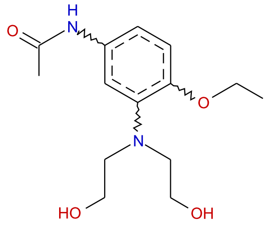	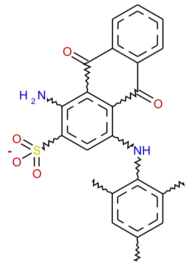	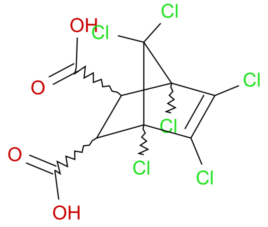
Actual Endpoint	Non-Irritant	Irritant	Irritant
Predicted Endpoint	Non-Irritant	Non-Irritant	Irritant
Distance	0.788	0.830	0.855
Reference	28ZPAK -,100,72	85JCAE "Prehled Prumyslove Toxikologie; Organicke Latky," Marhold, J., Prague , Czechoslovakia, Avicenum, 1986 Volume(issue)/page/year: -,1327,1986	85JCAE "Prehled Prumyslove Toxikologie; Organicke Latky," Marhold, J., Prague , Czechoslovakia, Avicenum, 1986 Volume(issue)/page/year: -,581,1986

Model Applicability

Unknown features are fingerprint features in the query molecule, but not found or appearing too infrequently in the training set.

1. All properties and OPS components are within expected ranges.

Feature Contribution

Top features for positive contribution				
**Fingerprint**	**Bit/Smiles**	**Feature Structure**	**Score**	**Irritant in training set**

Sorafenib

TOPKAT_Skin_Irritancy_None_vs_Irritant

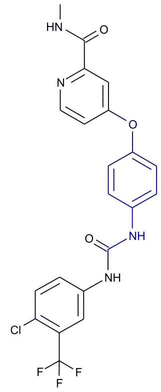

$C_{21}H_{16}ClF_3N_4O_3$

Molecular Weight: 464.82494

ALogP: 4.175

Rotatable Bonds: 6

Acceptors: 4

Donors: 3

Model Prediction

Prediction: Non-Irritant

Probability: 0.264

Enrichment: 0.287

Bayesian Score: -5.23

Mahalanobis Distance: 8.27

Mahalanobis Distance p-value: 0.791

Prediction: Positive if the Bayesian score is above the estimated best cutoff value from minimizing the false positive and false negative rate.
Probability: The esimated probability that the sample is in the positive category. This assumes that the Bayesian score follows a normal distribution and is different from the prediction using a cutoff.
Enrichment: An estimate of enrichment, that is, the increased likelihood (versus random) of this sample being in the category.
Bayesian Score: The standard Laplacian-modified Bayesian score.
Mahalanobis Distance: The Mahalanobis distance (MD) is the distance to the center of the training data. The larger the MD, the less trustworthy the prediction.
Mahalanobis Distance p-value: The p-value gives the fraction of training data with an MD greater than or equal to the one for the given sample, assuming normally distributed data. The smaller the p-value, the less trustworthy the prediciton. For highly non-normal X properties (e.g., fingerprints), the MD p-value is wildly inaccurate.

Structural Similar Compounds

Name	5-Norbornene-2,3-dicarboxylic acid, 1,4,5,6,7,7-hexachloro-	Benzenesulfonic acid, 2,2'-(4,4'-biphenylylenedivinylene)di-, disod ium salt	Sulfide, bis(4-t-butyl-m-cresyl)-
Structure	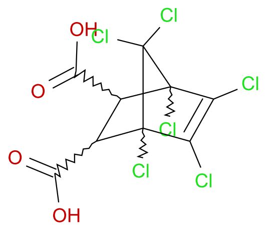	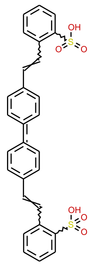	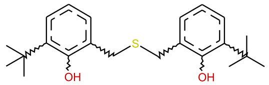
Actual Endpoint	Irritant	Irritant	Irritant
Predicted Endpoint	Irritant	Non-Irritant	Irritant
Distance	0.844	0.871	0.884
Reference	85JCAE "Prehled Prumyslove Toxikologie; Organicke Latky," Marhold, J., Prague , Czechoslovakia, Avicenum, 1986 Volume(issue)/page/year: -,581,1986	MVCRB3 MVC-Report. (Stockholm, Sweden) No.1-2, 1972-73. Discontinued. Volu me(issue)/page/year: 2,193,1973	AMIHBC AMA Archives of Industrial Hygiene and Occupational Medicine. (Chicago , IL) V.2-10, 1950-54. For publisher information, see AEHLAU. Volume(issue)/pag e/year: 5,311,1952

Model Applicability

Unknown features are fingerprint features in the query molecule, but not found or appearing too infrequently in the training set.

1. All properties and OPS components are within expected ranges.

Feature Contribution

Top features for positive contribution

Fingerprint	Bit/Smiles	Feature Structure	Score	Irritant in training set

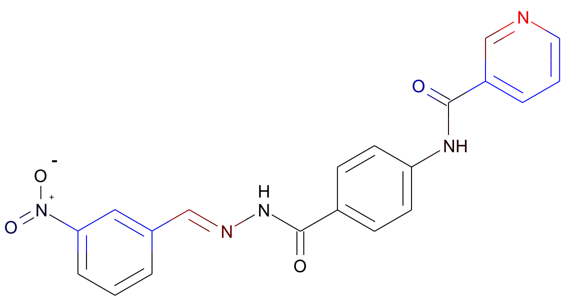

$C_{20}H_{15}N_5O_4$
Molecular Weight: 389.3642
ALogP: 2.211
Rotatable Bonds: 6
Acceptors: 6
Donors: 2

Model Prediction

Prediction: 28.4
Unit: mg/kg_body_weight/day
Mahalanobis Distance: 12.2
Mahalanobis Distance p-value: 7.56e-006

Mahalanobis Distance: The Mahalanobis distance (MD) is a generalization of the Euclidean distance that accounts for correlations among the X properties. It is calculated as the distance to the center of the training data. The larger the MD, the less trustworthy the prediction.
Mahalanobis Distance p-value: The p-value gives the fraction of training data with an MD greater than or equal to the one for the given sample, assuming normally distributed data. The smaller the p-value, the less trustworthy the prediciton. For highly non-normal X properties (e.g., fingerprints), the MD p-value is wildly inaccurate.

Structural Similar Compounds

Name	470	542	Ochratoxin A
Structure	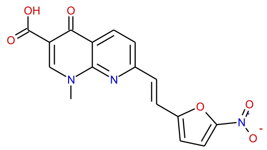	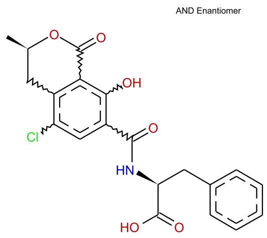 AND Enantiomer	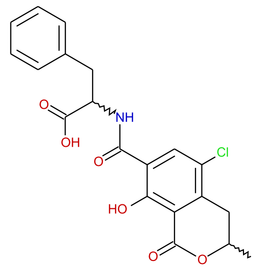
Actual Endpoint (-log C)	4.62839	4.79932	4.79932
Predicted Endpoint (-log C)	3.93264	3.6353	3.6353
Distance	0.687	0.688	0.688
Reference	CPDB	CPDB	CPDB

Model Applicability

Unknown features are fingerprint features in the query molecule, but not found or appearing too infrequently in the training set.

1. All properties and OPS components are within expected ranges.

Feature Contribution

Top features for positive contribution			
**Fingerprint**	**Bit/Smiles**	**Feature Structure**	**Score**
ECFP_6	655739385	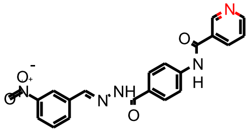 [*]:n:[*]	0.229

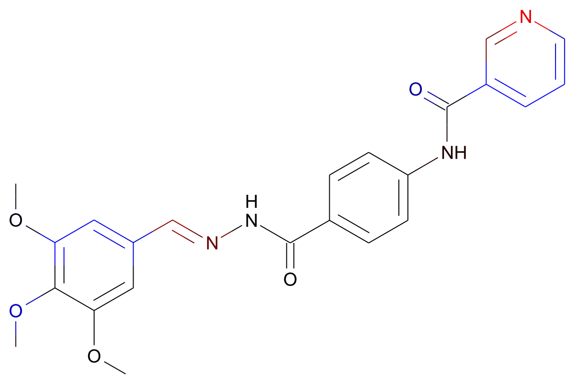

$C_{23}H_{22}N_4O_5$
Molecular Weight: 434.44457
ALogP: 2.268
Rotatable Bonds: 8
Acceptors: 7
Donors: 2

Structural Similar Compounds

Name	422	832	Compound LY171883
Structure	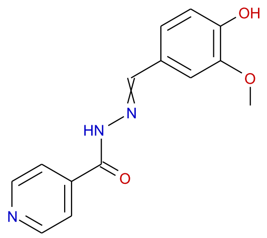	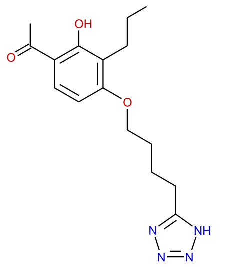	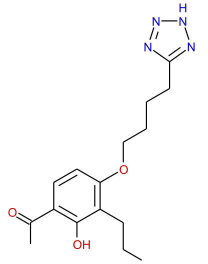
Actual Endpoint (-log C)	3.99565	3.45372	3.45372
Predicted Endpoint (-log C)	3.22211	2.80429	2.84749
Distance	0.691	0.714	0.719
Reference	CPDB	CPDB	CPDB

Model Applicability

Unknown features are fingerprint features in the query molecule, but not found or appearing too infrequently in the training set.

1. All properties and OPS components are within expected ranges.

Model Prediction

Prediction: 42.5
Unit: mg/kg_body_weight/day
Mahalanobis Distance: 11.8
Mahalanobis Distance p-value: 4.89e-005

Mahalanobis Distance: The Mahalanobis distance (MD) is a generalization of the Euclidean distance that accounts for correlations among the X properties. It is calculated as the distance to the center of the training data. The larger the MD, the less trustworthy the prediction.
Mahalanobis Distance p-value: The p-value gives the fraction of training data with an MD greater than or equal to the one for the given sample, assuming normally distributed data. The smaller the p-value, the less trustworthy the prediciton. For highly non-normal X properties (e.g., fingerprints), the MD p-value is wildly inaccurate.

Feature Contribution

Top features for positive contribution

Fingerprint	Bit/Smiles	Feature Structure	Score
ECFP_6	655739385	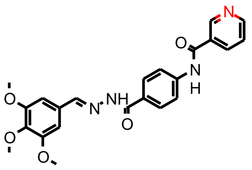 [*]:n:[*]	0.229

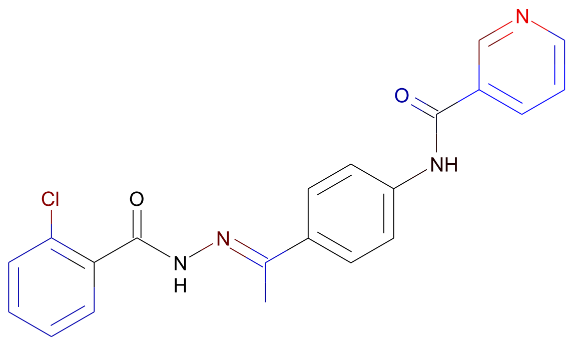

$C_{21}H_{17}ClN_4O_2$
Molecular Weight: 392.83827
ALogP: 2.962
Rotatable Bonds: 5
Acceptors: 4
Donors: 2

Model Prediction

Prediction: 26.4
Unit: mg/kg_body_weight/day
Mahalanobis Distance: 12.3
Mahalanobis Distance p-value: 4.14e-006

Mahalanobis Distance: The Mahalanobis distance (MD) is a generalization of the Euclidean distance that accounts for correlations among the X properties. It is calculated as the distance to the center of the training data. The larger the MD, the less trustworthy the prediction.
Mahalanobis Distance p-value: The p-value gives the fraction of training data with an MD greater than or equal to the one for the given sample, assuming normally distributed data. The smaller the p-value, the less trustworthy the prediciton. For highly non-normal X properties (e.g., fingerprints), the MD p-value is wildly inaccurate.

Structural Similar Compounds

Name	455	Phenolphthalein	44
Structure	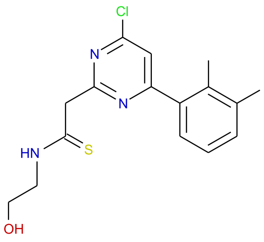	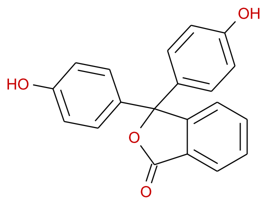	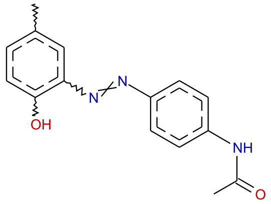
Actual Endpoint (-log C)	3.87681	2.43468	2.42163
Predicted Endpoint (-log C)	3.77582	3.66084	2.85113
Distance	0.688	0.691	0.697
Reference	CPDB	CPDB	CPDB

Model Applicability

Unknown features are fingerprint features in the query molecule, but not found or appearing too infrequently in the training set.

1. All properties and OPS components are within expected ranges.
2. Unknown ECFP_2 feature: 128986386: [*]\N=C(/C)\[c](:[*]):[*]
3. Unknown ECFP_2 feature: 560380707: [*]NN=C([*])[*]

Feature Contribution

Top features for positive contribution			
**Fingerprint**	**Bit/Smiles**	**Feature Structure**	**Score**
ECFP_6	655739385	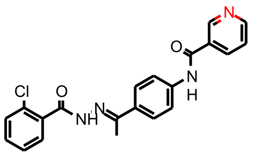 [*]:n:[*]	0.229

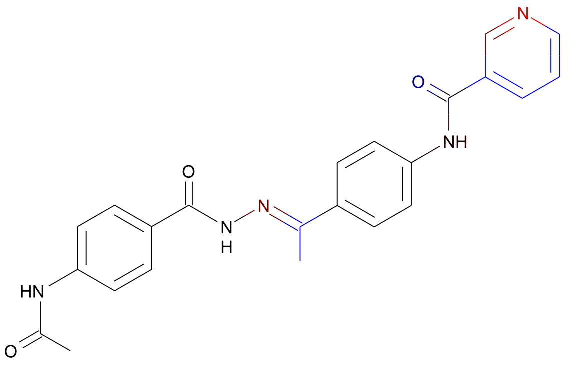

$C_{23}H_{21}N_5O_3$
Molecular Weight: 415.44453
ALogP: 1.418
Rotatable Bonds: 6
Acceptors: 5
Donors: 3

Model Prediction

Prediction: 28.1
Unit: mg/kg_body_weight/day
Mahalanobis Distance: 11.5
Mahalanobis Distance p-value: 0.000179

Mahalanobis Distance: The Mahalanobis distance (MD) is a generalization of the Euclidean distance that accounts for correlations among the X properties. It is calculated as the distance to the center of the training data. The larger the MD, the less trustworthy the prediction.
Mahalanobis Distance p-value: The p-value gives the fraction of training data with an MD greater than or equal to the one for the given sample, assuming normally distributed data. The smaller the p-value, the less trustworthy the prediciton. For highly non-normal X properties (e.g., fingerprints), the MD p-value is wildly inaccurate.

Structural Similar Compounds

Name	Ochratoxin A	542	4-Chloro-6-(2,3-xylidino)-2-pyri-mi-dinylthio(N-b-hydroxy-ethyl) acetamide
Structure	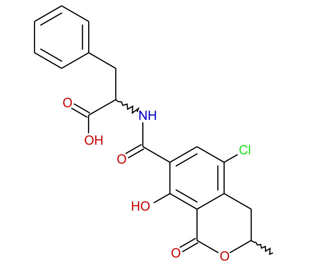	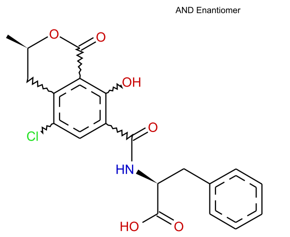	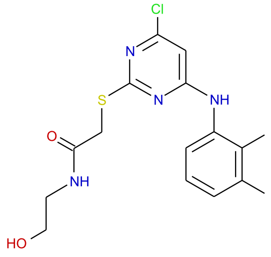
Actual Endpoint (-log C)	4.79932	4.79932	3.91517
Predicted Endpoint (-log C)	3.6353	3.6353	3.92186
Distance	0.686	0.686	0.722
Reference	CPDB	CPDB	CPDB

Model Applicability

Unknown features are fingerprint features in the query molecule, but not found or appearing too infrequently in the training set.

1. All properties and OPS components are within expected ranges.
2. Unknown ECFP_2 feature: 128986386: [*]\N=C(/C)\[c](:[*]):[*]
3. Unknown ECFP_2 feature: 560380707: [*]NN=C([*])[*]

Feature Contribution

Top features for positive contribution

Fingerprint	Bit/Smiles	Feature Structure	Score
ECFP_6	655739385	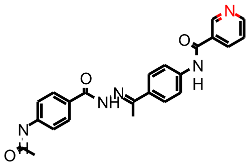 [*]:n:[*]	0.229

Sorafenib

TOPKAT_Carcinogenic_Potency_TD50_Mouse

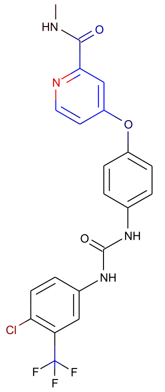

$C_{21}H_{16}ClF_3N_4O_3$

Molecular Weight: 464.82494

ALogP: 4.175

Rotatable Bonds: 6

Acceptors: 4

Donors: 3

Model Prediction

Prediction: 19.2

Unit: mg/kg_body_weight/day

Mahalanobis Distance: 12.4

Mahalanobis Distance p-value: 2.94e-006

Mahalanobis Distance: The Mahalanobis distance (MD) is a generalization of the Euclidean distance that accounts for correlations among the X properties. It is calculated as the distance to the center of the training data. The larger the MD, the less trustworthy the prediction.
Mahalanobis Distance p-value: The p-value gives the fraction of training data with an MD greater than or equal to the one for the given sample, assuming normally distributed data. The smaller the p-value, the less trustworthy the prediciton. For highly non-normal X properties (e.g., fingerprints), the MD p-value is wildly inaccurate.

Structural Similar Compounds

Name	Ochratoxin A	542	4-Chloro-6-(2,3-xylidino)-2-pyri-mi-dinylthio(N-b-hydroxy-ethyl) acetamide
Structure	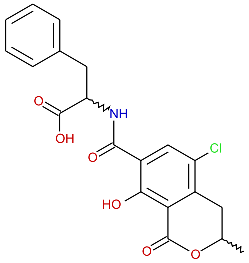	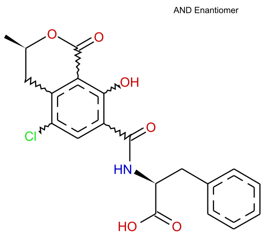	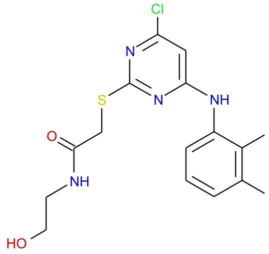
Actual Endpoint (-log C)	4.79932	4.79932	3.91517
Predicted Endpoint (-log C)	3.6353	3.6353	3.92186
Distance	0.718	0.718	0.738
Reference	CPDB	CPDB	CPDB

Model Applicability

Unknown features are fingerprint features in the query molecule, but not found or appearing too infrequently in the training set.

1. All properties and OPS components are within expected ranges.
2. Unknown ECFP_2 feature: 1413420509: [*]C(=[*])[c](:[cH]:[*]):n:[*]
3. Unknown ECFP_2 feature: 1338334141: [*]C(=[*])NC

Feature Contribution

Top features for positive contribution

Fingerprint	Bit/Smiles	Feature Structure	Score
ECFP_6	655739385	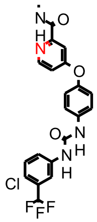 [*]:n:[*]	0.229

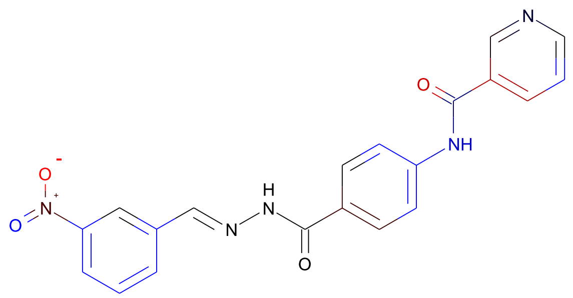

$C_{20}H_{15}N_5O_4$
Molecular Weight: 389.3642
ALogP: 2.211
Rotatable Bonds: 6
Acceptors: 6
Donors: 2

Structural Similar Compounds

Name	4-Bis(2-hydroxyethyl)amino-2-(5-nitro-2-thienyl)quinazoline	4-(2-Hydroxyethylamino)-2-(5-nitro-2-thienyl)quinazoline	Salicylazosulfapyridine
Structure	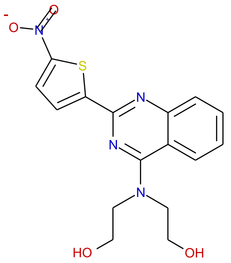	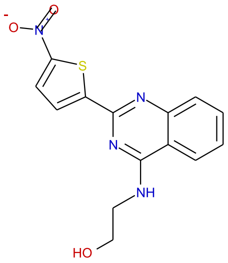	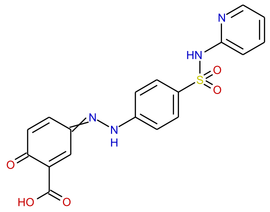
Actual Endpoint (-log C)	5.05984	5.22831	2.39891
Predicted Endpoint (-log C)	4.23808	4.31976	3.17598
Distance	0.564	0.604	0.628
Reference	CPDB	CPDB	CPDB

Model Prediction

Prediction: 25.8
Unit: mg/kg_body_weight/day
Mahalanobis Distance: 12.6
Mahalanobis Distance p-value: 4.11e-005

Mahalanobis Distance: The Mahalanobis distance (MD) is a generalization of the Euclidean distance that accounts for correlations among the X properties. It is calculated as the distance to the center of the training data. The larger the MD, the less trustworthy the prediction.
Mahalanobis Distance p-value: The p-value gives the fraction of training data with an MD greater than or equal to the one for the given sample, assuming normally distributed data. The smaller the p-value, the less trustworthy the prediciton. For highly non-normal X properties (e.g., fingerprints), the MD p-value is wildly inaccurate.

Model Applicability

Unknown features are fingerprint features in the query molecule, but not found or appearing too infrequently in the training set.

1. OPS PC24 out of range. Value: -3.3565. Training min, max, SD, explained variance: -3.0088, 7.4204, 1.076, 0.0111.

Feature Contribution

Top features for positive contribution

Fingerprint	Bit/Smiles	Feature Structure	Score
FCFP_6	5	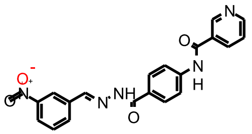 [*][O-]	0.431

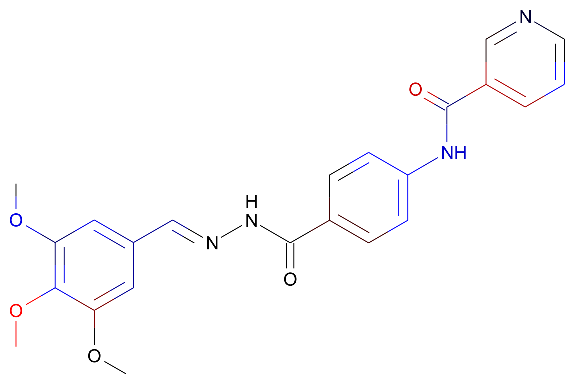

$C_{23}H_{22}N_4O_5$
Molecular Weight: 434.44457
ALogP: 2.268
Rotatable Bonds: 8
Acceptors: 7
Donors: 2

Model Prediction

Prediction: 6.21
Unit: mg/kg_body_weight/day
Mahalanobis Distance: 13.9
Mahalanobis Distance p-value: 3.35e-008

Mahalanobis Distance: The Mahalanobis distance (MD) is a generalization of the Euclidean distance that accounts for correlations among the X properties. It is calculated as the distance to the center of the training data. The larger the MD, the less trustworthy the prediction.
Mahalanobis Distance p-value: The p-value gives the fraction of training data with an MD greater than or equal to the one for the given sample, assuming normally distributed data. The smaller the p-value, the less trustworthy the prediciton. For highly non-normal X properties (e.g., fingerprints), the MD p-value is wildly inaccurate.

Structural Similar Compounds

Name	C.I. direct brown 95	Loxtidine	Salicylazosulfapyridine
Structure	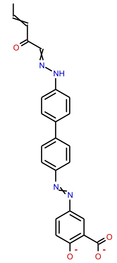	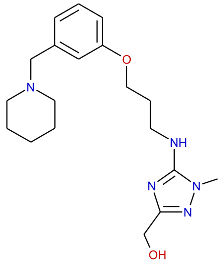	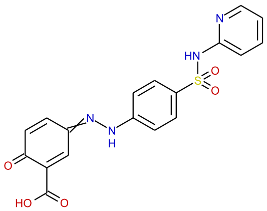
Actual Endpoint (-log C)	5.31387	2.87532	2.39891
Predicted Endpoint (-log C)	4.30266	3.63996	3.17598
Distance	0.631	0.674	0.688
Reference	CPDB	CPDB	CPDB

Model Applicability

Unknown features are fingerprint features in the query molecule, but not found or appearing too infrequently in the training set.

1. All properties and OPS components are within expected ranges.

Feature Contribution

Top features for positive contribution

Fingerprint	Bit/Smiles	Feature Structure	Score
FCFP_6	136627117	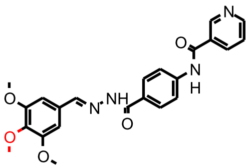 [*]OC	0.69

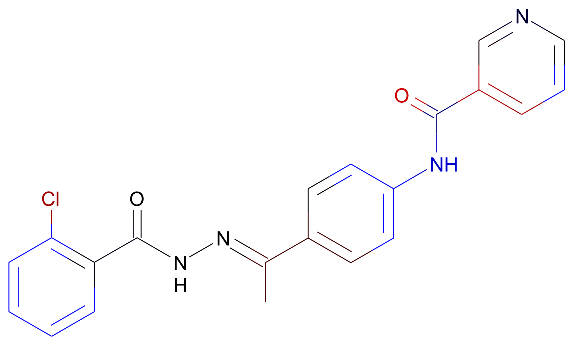

$C_{21}H_{17}ClN_4O_2$
Molecular Weight: 392.83827
ALogP: 2.962
Rotatable Bonds: 5
Acceptors: 4
Donors: 2

Model Prediction

Prediction: 29.4
Unit: mg/kg_body_weight/day
Mahalanobis Distance: 12.5
Mahalanobis Distance p-value: 6.85e-005

Mahalanobis Distance: The Mahalanobis distance (MD) is a generalization of the Euclidean distance that accounts for correlations among the X properties. It is calculated as the distance to the center of the training data. The larger the MD, the less trustworthy the prediction.
Mahalanobis Distance p-value: The p-value gives the fraction of training data with an MD greater than or equal to the one for the given sample, assuming normally distributed data. The smaller the p-value, the less trustworthy the prediciton. For highly non-normal X properties (e.g., fingerprints), the MD p-value is wildly inaccurate.

Structural Similar Compounds

Name	Omeprazole	3-(Cyclopentyloxy)-N-(3,5-di-chloro-4-pyridyl)-4-methoxy-benzamide	Indomethacin
Structure	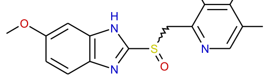	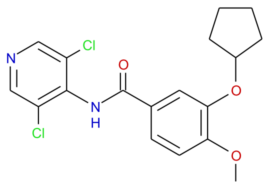	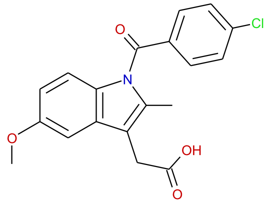
Actual Endpoint (-log C)	3.4628	5.39369	5.49293
Predicted Endpoint (-log C)	4.7324	4.27874	4.9569
Distance	0.603	0.611	0.612
Reference	CPDB	CPDB	CPDB

Model Applicability

Unknown features are fingerprint features in the query molecule, but not found or appearing too infrequently in the training set.

1. OPS PC6 out of range. Value: 7.0847. Training min, max, SD, explained variance: -5.5832, 6.4847, 1.973, 0.0374.

Feature Contribution

Top features for positive contribution			
**Fingerprint**	**Bit/Smiles**	**Feature Structure**	**Score**
FCFP_6	1	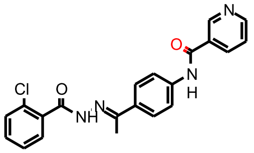 [*]=O	0.234

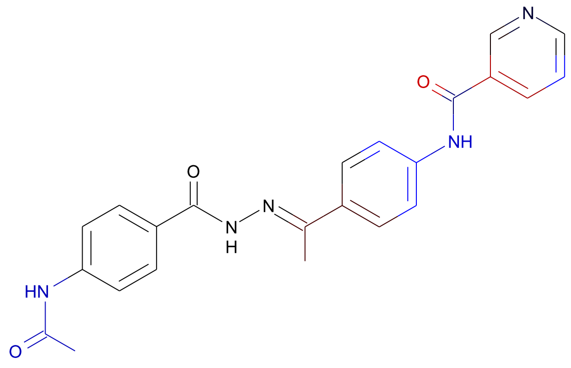

$C_{23}H_{21}N_5O_3$

Molecular Weight: 415.44453

ALogP: 1.418

Rotatable Bonds: 6

Acceptors: 5

Donors: 3

Model Prediction

Prediction: 30.7

Unit: mg/kg_body_weight/day

Mahalanobis Distance: 10.7

Mahalanobis Distance p-value: 0.0416

Mahalanobis Distance: The Mahalanobis distance (MD) is a generalization of the Euclidean distance that accounts for correlations among the X properties. It is calculated as the distance to the center of the training data. The larger the MD, the less trustworthy the prediction.

Mahalanobis Distance p-value: The p-value gives the fraction of training data with an MD greater than or equal to the one for the given sample, assuming normally distributed data. The smaller the p-value, the less trustworthy the prediciton. For highly non-normal X properties (e.g., fingerprints), the MD p-value is wildly inaccurate.

Structural Similar Compounds

Name	4,4´-Sulfonylbisacetanilide	Ochratoxin A	542
Structure	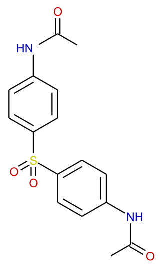	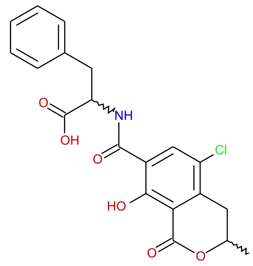	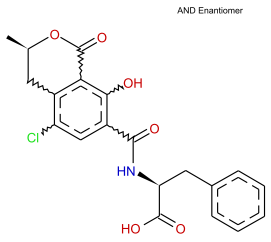
Actual Endpoint (-log C)	3.77655	6.47264	6.59334
Predicted Endpoint (-log C)	3.55337	5.06501	5.06501
Distance	0.627	0.655	0.655
Reference	CPDB	CPDB	CPDB

Model Applicability

Unknown features are fingerprint features in the query molecule, but not found or appearing too infrequently in the training set.

1. All properties and OPS components are within expected ranges.

Feature Contribution

Top features for positive contribution

Fingerprint	Bit/Smiles	Feature Structure	Score
FCFP_6	1	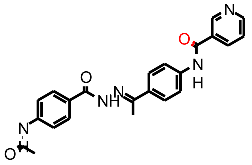 [*]=O	0.234

Sorafenib

TOPKAT_Carcinogenic_Potency_TD50_Rat

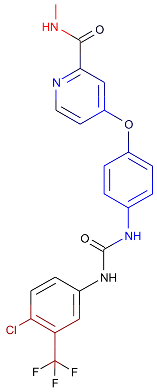

$C_{21}H_{16}ClF_3N_4O_3$

Molecular Weight: 464.82494

ALogP: 4.175

Rotatable Bonds: 6

Acceptors: 4

Donors: 3

Model Prediction

Prediction: 14.2

Unit: mg/kg_body_weight/day

Mahalanobis Distance: 20.4

Mahalanobis Distance p-value: 9.56e-031

Mahalanobis Distance: The Mahalanobis distance (MD) is a generalization of the Euclidean distance that accounts for correlations among the X properties. It is calculated as the distance to the center of the training data. The larger the MD, the less trustworthy the prediction.
Mahalanobis Distance p-value: The p-value gives the fraction of training data with an MD greater than or equal to the one for the given sample, assuming normally distributed data. The smaller the p-value, the less trustworthy the prediciton. For highly non-normal X properties (e.g., fingerprints), the MD p-value is wildly inaccurate.

Structural Similar Compounds

Name	Fluvastatin	913	Ochratoxin A
Structure	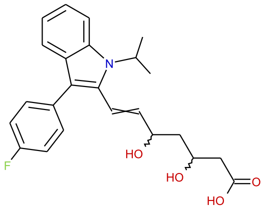	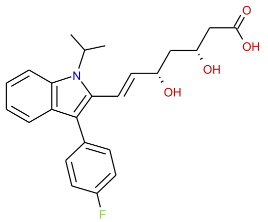	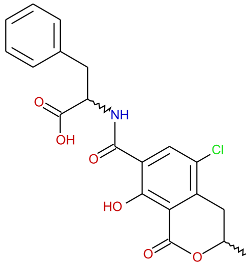
Actual Endpoint (-log C)	3.51742	3.51742	6.47264
Predicted Endpoint (-log C)	5.41573	5.41573	5.06501
Distance	0.597	0.597	0.666
Reference	CPDB	CPDB	CPDB

Model Applicability

Unknown features are fingerprint features in the query molecule, but not found or appearing too infrequently in the training set.

1. All properties and OPS components are within expected ranges.
2. Unknown FCFP_2 feature: -1029533685: [*]:[c](:[*])C(F)(F)F

Feature Contribution

Top features for positive contribution

Fingerprint	Bit/Smiles	Feature Structure	Score
FCFP_6	1	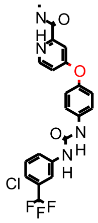 [*]=O	0.234

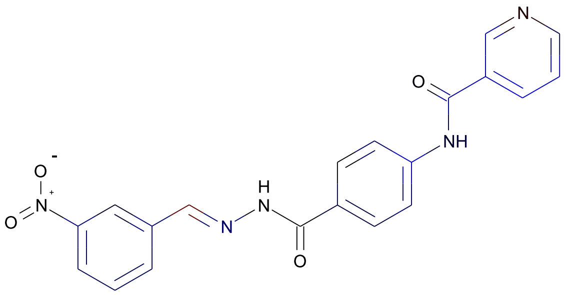

$C_{20}H_{15}N_5O_4$

Molecular Weight: 389.3642

ALogP: 2.211

Rotatable Bonds: 6

Acceptors: 6

Donors: 2

Model Prediction

Prediction: 0.0774

Unit: g/kg_body_weight

Mahalanobis Distance: 29

Mahalanobis Distance p-value: 6.55e-023

Mahalanobis Distance: The Mahalanobis distance (MD) is a generalization of the Euclidean distance that accounts for correlations among the X properties. It is calculated as the distance to the center of the training data. The larger the MD, the less trustworthy the prediction.
Mahalanobis Distance p-value: The p-value gives the fraction of training data with an MD greater than or equal to the one for the given sample, assuming normally distributed data. The smaller the p-value, the less trustworthy the prediciton. For highly non-normal X properties (e.g., fingerprints), the MD p-value is wildly inaccurate.

Structural Similar Compounds

Name	GLIPIZIDE	CHLORSULFURON	DANTROLENE.NA
Structure	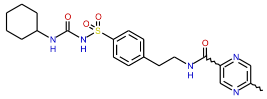	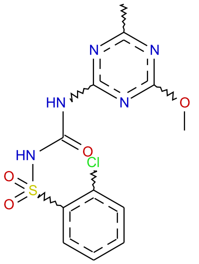	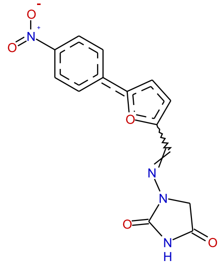
Actual Endpoint (-log C)	3.94991	4.15566	4.19625
Predicted Endpoint (-log C)	3.95594	3.79771	4.62637
Distance	0.634	0.643	0.669
Reference	NDA-17583	EPA COVER SHEET 0027;880301;(1)	NDA-17443

Model Applicability

Unknown features are fingerprint features in the query molecule, but not found or appearing too infrequently in the training set.

1. All properties and OPS components are within expected ranges.
2. Unknown FCFP_2 feature: 5: [*][O-]
3. Unknown FCFP_2 feature: -828984032: [*][N+](=[*])[c](:[cH]:[*]):[cH]:[*]
4. Unknown FCFP_2 feature: -1338588315: [*]:[c](:[*])[N+](=O)[O-]
5. Unknown FCFP_2 feature: 1872392852: [*][N+](=O)[*]
6. Unknown FCFP_2 feature: 260476081: [*][N+](=[*])[O-]
7. Unknown ECFP_6 feature: 1043790491: [*][N+](=[*])[*]
8. Unknown ECFP_6 feature: 781519895: [*][O-]
9. Unknown ECFP_6 feature: 1997021792: [*]:[cH]:[cH]:[cH]:[*]
10. Unknown ECFP_6 feature: 1996163143: [*]:[cH]:[cH]:n:[*]
11. Unknown ECFP_6 feature: -677055651: [*]:[cH]:n:[cH]:[*]
12. Unknown ECFP_6 feature: -709633021: [*][c](:[*]):[cH]:n:[*]
13. Unknown ECFP_6 feature: -175146122: [*]C(=[*])[c](:[cH]:[*]):[cH]:[*]
14. Unknown ECFP_6 feature: 1430169877: [*]NC(=O)[c](:[*]):[*]
15. Unknown ECFP_6 feature: -177077903: [*]N[c](:[cH]:[*]):[cH]:[*]
16. Unknown ECFP_6 feature: 544048674: [*]C(=[*])NN=[*]
17. Unknown ECFP_6 feature: 1814278164: [*]N\N=C\[*]
18. Unknown ECFP_6 feature: -1832102709: [*]\N=C\[c](:[*]):[*]
19. Unknown ECFP_6 feature: -176483725: [*]=C[c](:[cH]:[*]):[cH]:[*]

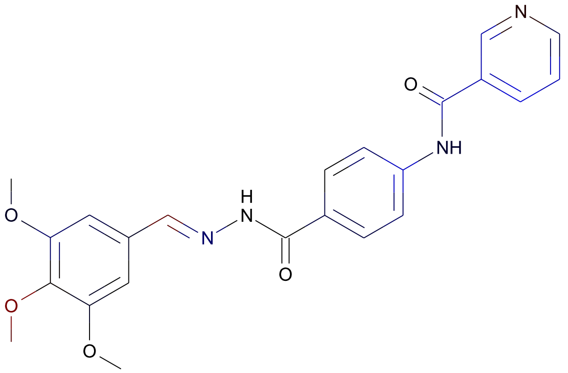

$C_{23}H_{22}N_4O_5$

Molecular Weight: 434.44457

ALogP: 2.268

Rotatable Bonds: 8

Acceptors: 7

Donors: 2

Model Prediction

Prediction: 0.0909

Unit: g/kg_body_weight

Mahalanobis Distance: 30.4

Mahalanobis Distance p-value: 2.22e-025

Mahalanobis Distance: The Mahalanobis distance (MD) is a generalization of the Euclidean distance that accounts for correlations among the X properties. It is calculated as the distance to the center of the training data. The larger the MD, the less trustworthy the prediction.
Mahalanobis Distance p-value: The p-value gives the fraction of training data with an MD greater than or equal to the one for the given sample, assuming normally distributed data. The smaller the p-value, the less trustworthy the prediciton. For highly non-normal X properties (e.g., fingerprints), the MD p-value is wildly inaccurate.

Structural Similar Compounds

Name	GLIPIZIDE	GLYBURIDE	CHLORSULFURON
Structure	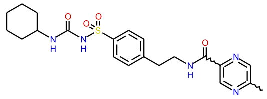	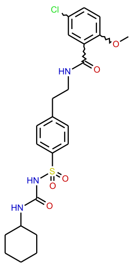	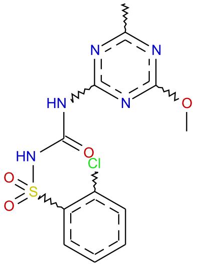
Actual Endpoint (-log C)	3.94991	4.21661	4.15566
Predicted Endpoint (-log C)	3.95594	4.21035	3.79771
Distance	0.688	0.714	0.727
Reference	NDA-17583	UPJ-26452	EPA COVER SHEET 0027;880301;(1)

Model Applicability

Unknown features are fingerprint features in the query molecule, but not found or appearing too infrequently in the training set.

1. OPS PC18 out of range. Value: -5.1484. Training min, max, SD, explained variance: -4.7991, 6.1674, 1.831, 0.0147.
2. Unknown ECFP_6 feature: 1997021792: [*]:[cH]:[cH]:[cH]:[*]
3. Unknown ECFP_6 feature: 1996163143: [*]:[cH]:[cH]:n:[*]
4. Unknown ECFP_6 feature: -677055651: [*]:[cH]:n:[cH]:[*]
5. Unknown ECFP_6 feature: -709633021: [*][c](:[*]):[cH]:n:[*]
6. Unknown ECFP_6 feature: -175146122: [*]C(=[*])[c](:[cH]:[*]):[cH]:[*]
7. Unknown ECFP_6 feature: 1430169877: [*]NC(=O)[c](:[*]):[*]
8. Unknown ECFP_6 feature: -177077903: [*]N[c](:[cH]:[*]):[cH]:[*]
9. Unknown ECFP_6 feature: 544048674: [*]C(=[*])NN=[*]
10. Unknown ECFP_6 feature: 1814278164: [*]N\N=C\[*]
11. Unknown ECFP_6 feature: -1832102709: [*]\N=C\[c](:[*]):[*]
12. Unknown ECFP_6 feature: -176483725: [*]=C[c](:[cH]:[*]):[cH]:[*]
13. Unknown ECFP_6 feature: -1531301414: [*]O[c](:[c]([*]):[*]):[c]([*]):[*]
14. Unknown ECFP_6 feature: 1307307440: [*]:[c](:[*])OC

Feature Contribution

Top features for positive contribution

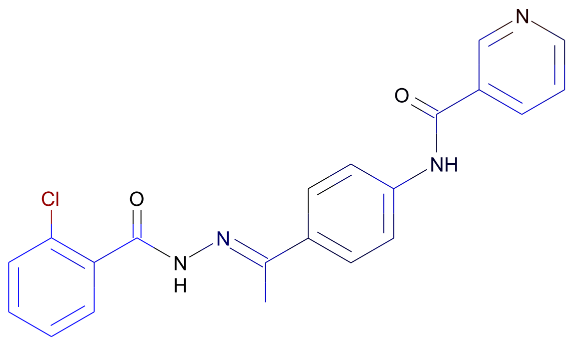

$C_{21}H_{17}ClN_4O_2$
Molecular Weight: 392.83827
ALogP: 2.962
Rotatable Bonds: 5
Acceptors: 4
Donors: 2

Model Prediction

Prediction: 0.639
Unit: g/kg_body_weight
Mahalanobis Distance: 27.4
Mahalanobis Distance p-value: 7.45e-020

Mahalanobis Distance: The Mahalanobis distance (MD) is a generalization of the Euclidean distance that accounts for correlations among the X properties. It is calculated as the distance to the center of the training data. The larger the MD, the less trustworthy the prediction.
Mahalanobis Distance p-value: The p-value gives the fraction of training data with an MD greater than or equal to the one for the given sample, assuming normally distributed data. The smaller the p-value, the less trustworthy the prediciton. For highly non-normal X properties (e.g., fingerprints), the MD p-value is wildly inaccurate.

Structural Similar Compounds

Name	ISOXABEN	HC BLUE 1	SODIUM ACIFLUORFEN
Structure	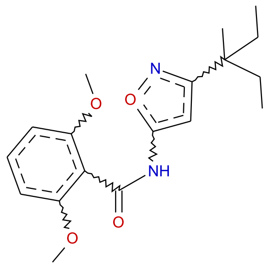	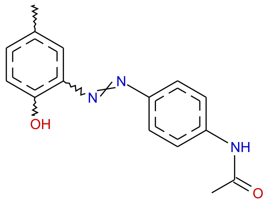	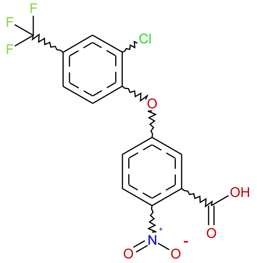
Actual Endpoint (-log C)	3.81665	3.0323	4.16036
Predicted Endpoint (-log C)	4.42315	2.7171	4.65915
Distance	0.660	0.664	0.680
Reference	EPA COVER SHEET 0339;881201;(1)	NTP REPORT # 222	EPA COVER SHEET 0192;891101;(1)

Model Applicability

Unknown features are fingerprint features in the query molecule, but not found or appearing too infrequently in the training set.

1. All properties and OPS components are within expected ranges.
2. Unknown ECFP_6 feature: 1997021792: [*]:[cH]:[cH]:[cH]:[*]
3. Unknown ECFP_6 feature: 1996163143: [*]:[cH]:[cH]:n:[*]
4. Unknown ECFP_6 feature: -677055651: [*]:[cH]:n:[cH]:[*]
5. Unknown ECFP_6 feature: -709633021: [*][c](:[*]):[cH]:n:[*]
6. Unknown ECFP_6 feature: -175146122: [*]C(=[*])[c](:[cH]:[*]):[cH]:[*]
7. Unknown ECFP_6 feature: 1430169877: [*]NC(=O)[c](:[*]):[*]
8. Unknown ECFP_6 feature: -177077903: [*]N[c](:[cH]:[*]):[cH]:[*]
9. Unknown ECFP_6 feature: 128986386: [*]\N=C(/C)\[c](:[*]):[*]
10. Unknown ECFP_6 feature: 560380707: [*]NN=C([*])[*]
11. Unknown ECFP_6 feature: 544048674: [*]C(=[*])NN=[*]
12. Unknown ECFP_6 feature: 99947387: [*]:[c](:[*])Cl

Feature Contribution

Top features for positive contribution			
Fingerprint	Bit/Smiles	Feature Structure	Score

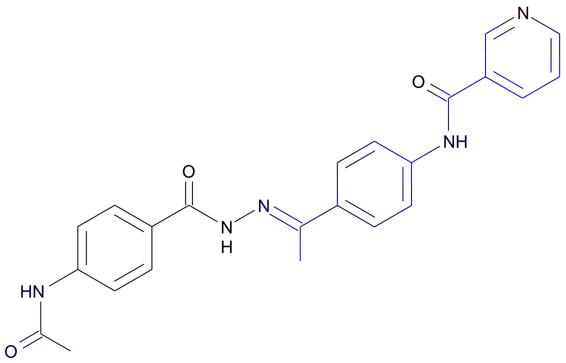

$C_{23}H_{21}N_5O_3$
Molecular Weight: 415.44453
ALogP: 1.418
Rotatable Bonds: 6
Acceptors: 5
Donors: 3

Model Prediction

Prediction: 0.315
Unit: g/kg_body_weight
Mahalanobis Distance: 27
Mahalanobis Distance p-value: 4.08e-019

Mahalanobis Distance: The Mahalanobis distance (MD) is a generalization of the Euclidean distance that accounts for correlations among the X properties. It is calculated as the distance to the center of the training data. The larger the MD, the less trustworthy the prediction.
Mahalanobis Distance p-value: The p-value gives the fraction of training data with an MD greater than or equal to the one for the given sample, assuming normally distributed data. The smaller the p-value, the less trustworthy the prediciton. For highly non-normal X properties (e.g., fingerprints), the MD p-value is wildly inaccurate.

Structural Similar Compounds

Name	GLIPIZIDE	CHLORSULFURON	FUROSEMIDE
Structure	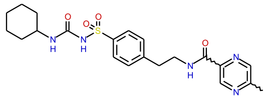	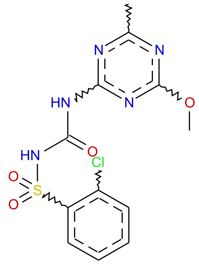	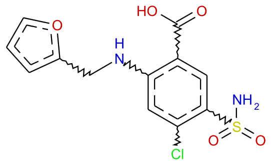
Actual Endpoint (-log C)	3.94991	4.15566	4.27645
Predicted Endpoint (-log C)	3.95594	3.79771	4.40005
Distance	0.630	0.727	0.734
Reference	NDA-17583	EPA COVER SHEET 0027;880301;(1)	NTP REPORT # 356

Model Applicability

Unknown features are fingerprint features in the query molecule, but not found or appearing too infrequently in the training set.

1. All properties and OPS components are within expected ranges.
2. Unknown ECFP_6 feature: 1997021792: [*]:[cH]:[cH]:[cH]:[*]
3. Unknown ECFP_6 feature: 1996163143: [*]:[cH]:[cH]:n:[*]
4. Unknown ECFP_6 feature: -677055651: [*]:[cH]:n:[cH]:[*]
5. Unknown ECFP_6 feature: -709633021: [*][c](:[*]):[cH]:n:[*]
6. Unknown ECFP_6 feature: -175146122: [*]C(=[*])[c](:[cH]:[*]):[cH]:[*]
7. Unknown ECFP_6 feature: 1430169877: [*]NC(=O)[c](:[*]):[*]
8. Unknown ECFP_6 feature: -177077903: [*]N[c](:[cH]:[*]):[cH]:[*]
9. Unknown ECFP_6 feature: 128986386: [*]\N=C(/C)\[c](:[*]):[*]
10. Unknown ECFP_6 feature: 560380707: [*]NN=C([*])[*]
11. Unknown ECFP_6 feature: 544048674: [*]C(=[*])NN=[*]
12. Unknown ECFP_6 feature: -474544785: [*]NC(=O)C

Feature Contribution

Top features for positive contribution			
Fingerprint	Bit/Smiles	Feature Structure	Score

Sorafenib

TOPKAT_Chronic_LOAEL

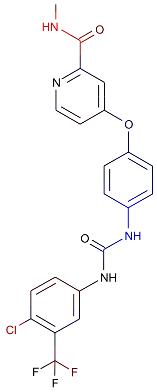

$C_{21}H_{16}ClF_3N_4O_3$

Molecular Weight: 464.82494

ALogP: 4.175

Rotatable Bonds: 6

Acceptors: 4

Donors: 3

Structural Similar Compounds

Name	GLYBURIDE	D & C RED 9	SODIUM ACIFLUORFEN
Structure	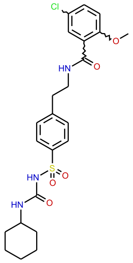	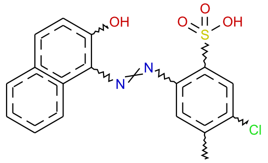	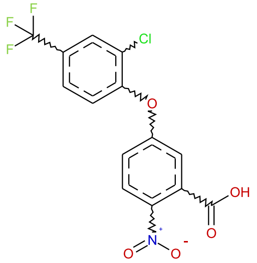
Actual Endpoint (-log C)	4.21661	3.87715	4.16036
Predicted Endpoint (-log C)	4.21035	3.6546	4.65915
Distance	0.636	0.722	0.736
Reference	UPJ-26452	NTP REPORT # 225	EPA COVER SHEET 0192;891101;(1)

Model Prediction

Prediction: 0.00483

Unit: g/kg_body_weight

Mahalanobis Distance: 30

Mahalanobis Distance p-value: 1.21e-024

Mahalanobis Distance: The Mahalanobis distance (MD) is a generalization of the Euclidean distance that accounts for correlations among the X properties. It is calculated as the distance to the center of the training data. The larger the MD, the less trustworthy the prediction.
Mahalanobis Distance p-value: The p-value gives the fraction of training data with an MD greater than or equal to the one for the given sample, assuming normally distributed data. The smaller the p-value, the less trustworthy the prediciton. For highly non-normal X properties (e.g., fingerprints), the MD p-value is wildly inaccurate.

Model Applicability

Unknown features are fingerprint features in the query molecule, but not found or appearing too infrequently in the training set.

1. All properties and OPS components are within expected ranges.
2. Unknown ECFP_6 feature: -1046436026: [*]F
3. Unknown ECFP_6 feature: 1305253718: [*]:[c](:[*])O[c](:[*]):[*]
4. Unknown ECFP_6 feature: 1413420509: [*]C(=[*])[c](:[cH]:[*]):n:[*]
5. Unknown ECFP_6 feature: -677309799: [*][c](:[*]):n:[cH]:[*]
6. Unknown ECFP_6 feature: 1996163143: [*]:[cH]:[cH]:n:[*]
7. Unknown ECFP_6 feature: 1430169877: [*]NC(=O)[c](:[*]):[*]
8. Unknown ECFP_6 feature: 1338334141: [*]C(=[*])NC
9. Unknown ECFP_6 feature: 864287155: [*]NC
10. Unknown ECFP_6 feature: -177077903: [*]N[c](:[cH]:[*]):[cH]:[*]
11. Unknown ECFP_6 feature: -649580166: [*]NC(=O)N[*]
12. Unknown ECFP_6 feature: 1336678434: [*][c](:[*]):[c](:[cH]:[*])C([*])([*])[*]
13. Unknown ECFP_6 feature: 99947387: [*]:[c](:[*])Cl
14. Unknown ECFP_6 feature: -1952889961: [*]:[c](:[*])C(F)(F)F
15. Unknown ECFP_6 feature: 226796801: [*]C([*])([*])F

Feature Contribution

Top features for positive contribution

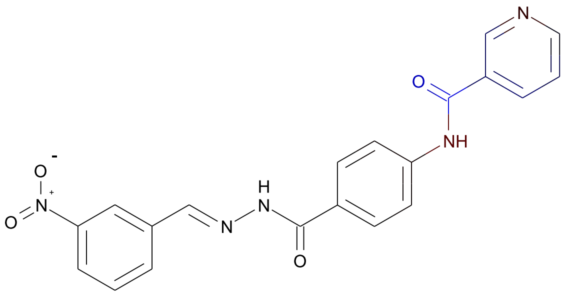

$C_{20}H_{15}N_5O_4$

Molecular Weight: 389.3642

ALogP: 2.211

Rotatable Bonds: 6

Acceptors: 6

Donors: 2

Model Prediction

Prediction: 0.0936

Unit: g/kg_body_weight

Mahalanobis Distance: 7

Mahalanobis Distance p-value: 0.197

Mahalanobis Distance: The Mahalanobis distance (MD) is a generalization of the Euclidean distance that accounts for correlations among the X properties. It is calculated as the distance to the center of the training data. The larger the MD, the less trustworthy the prediction.
Mahalanobis Distance p-value: The p-value gives the fraction of training data with an MD greater than or equal to the one for the given sample, assuming normally distributed data. The smaller the p-value, the less trustworthy the prediciton. For highly non-normal X properties (e.g., fingerprints), the MD p-value is wildly inaccurate.

Structural Similar Compounds

Name	SALICYLAZOSULFAPYRIDINE	FUROSEMIDE	DAPSONE
Structure	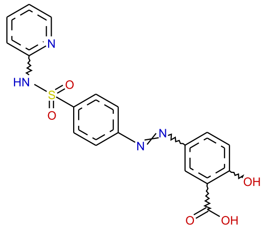	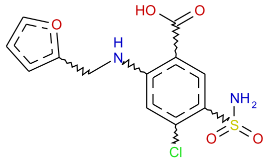	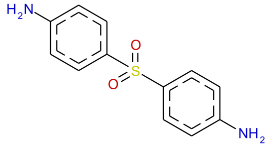
Actual Endpoint (-log C)	3.375	4.04236	3.66258
Predicted Endpoint (-log C)	2.80292	2.8614	3.26993
Distance	0.597	0.624	0.783
Reference	NCI/NTP TR-457	NCI/NTP TR-356	NCI/NTP TR-20

Model Applicability

Unknown features are fingerprint features in the query molecule, but not found or appearing too infrequently in the training set.

1. All properties and OPS components are within expected ranges.
2. Unknown FCFP_2 feature: 8: [*][N+](=[*])[*]
3. Unknown FCFP_2 feature: 5: [*][O-]
4. Unknown FCFP_2 feature: -828984032: [*][N+](=[*])[c](:[cH]:[*]):[cH]:[*]
5. Unknown FCFP_2 feature: -1338588315: [*]:[c](:[*])[N+](=O)[O-]
6. Unknown FCFP_2 feature: 1872392852: [*][N+](=O)[*]
7. Unknown FCFP_2 feature: 260476081: [*][N+](=[*])[O-]

Feature Contribution

Top features for positive contribution			
Fingerprint	Bit/Smiles	Feature Structure	Score

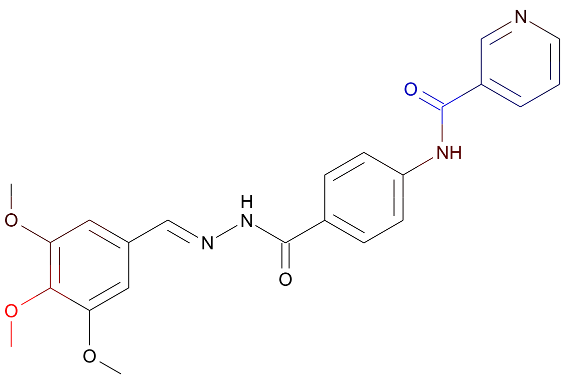

$C_{23}H_{22}N_4O_5$

Molecular Weight: 434.44457

ALogP: 2.268

Rotatable Bonds: 8

Acceptors: 7

Donors: 2

Structural Similar Compounds

Name	SALICYLAZOSULFAPYRIDINE	FUROSEMIDE	3,3'-DIMETHOXYBENZIDINE-4,4'-DIISOCYANATE
Structure	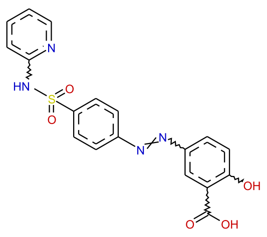	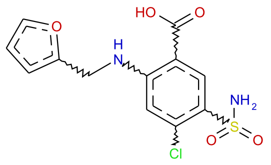	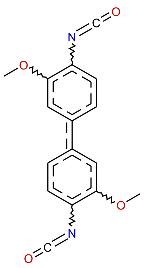
Actual Endpoint (-log C)	3.375	4.04236	2.17504
Predicted Endpoint (-log C)	2.80292	2.8614	3.78717
Distance	0.649	0.760	0.823
Reference	NCI/NTP TR-457	NCI/NTP TR-356	NCI/NTP TR-128

Model Prediction

Prediction: 0.0557

Unit: g/kg_body_weight

Mahalanobis Distance: 7.8

Mahalanobis Distance p-value: 0.0398

Mahalanobis Distance: The Mahalanobis distance (MD) is a generalization of the Euclidean distance that accounts for correlations among the X properties. It is calculated as the distance to the center of the training data. The larger the MD, the less trustworthy the prediction.
Mahalanobis Distance p-value: The p-value gives the fraction of training data with an MD greater than or equal to the one for the given sample, assuming normally distributed data. The smaller the p-value, the less trustworthy the prediciton. For highly non-normal X properties (e.g., fingerprints), the MD p-value is wildly inaccurate.

Model Applicability

Unknown features are fingerprint features in the query molecule, but not found or appearing too infrequently in the training set.

1. OPS PC9 out of range. Value: 4.0783. Training min, max, SD, explained variance: -2.8548, 3.3954, 1.263, 0.0360.

Feature Contribution

Top features for positive contribution			
**Fingerprint**	**Bit/Smiles**	**Feature Structure**	**Score**
FCFP_2	136627117	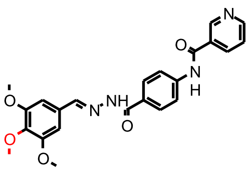 [*]OC	0.173

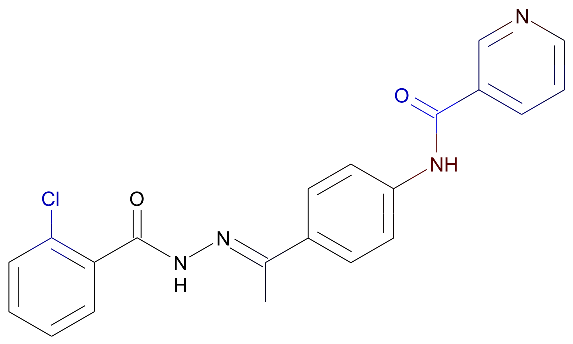

$C_{21}H_{17}ClN_4O_2$
Molecular Weight: 392.83827
ALogP: 2.962
Rotatable Bonds: 5
Acceptors: 4
Donors: 2

Model Prediction

Prediction: 0.142
Unit: g/kg_body_weight
Mahalanobis Distance: 8.84
Mahalanobis Distance p-value: 0.00237

Mahalanobis Distance: The Mahalanobis distance (MD) is a generalization of the Euclidean distance that accounts for correlations among the X properties. It is calculated as the distance to the center of the training data. The larger the MD, the less trustworthy the prediction.
Mahalanobis Distance p-value: The p-value gives the fraction of training data with an MD greater than or equal to the one for the given sample, assuming normally distributed data. The smaller the p-value, the less trustworthy the prediciton. For highly non-normal X properties (e.g., fingerprints), the MD p-value is wildly inaccurate.

Structural Similar Compounds

Name	PHENOLPHTHALEIN	DISPERSE YELLOW 3	OXAZEPAM
Structure	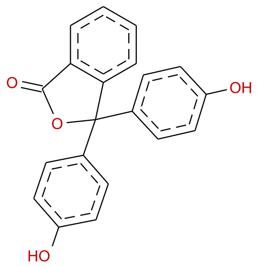	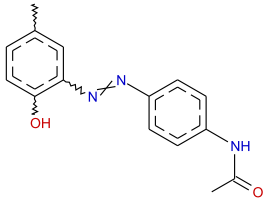	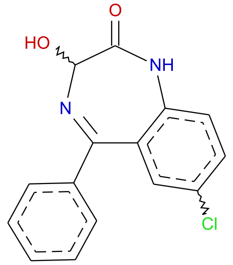
Actual Endpoint (-log C)	2.20184	2.77703	3.05262
Predicted Endpoint (-log C)	2.8857	2.80195	3.13073
Distance	0.624	0.637	0.638
Reference	NCI/NTP TR-465	NCI/NTP TR-222	NCI/NTP TR-468

Model Applicability

Unknown features are fingerprint features in the query molecule, but not found or appearing too infrequently in the training set.

1. All properties and OPS components are within expected ranges.

Feature Contribution

Top features for positive contribution

Fingerprint	Bit/Smiles	Feature Structure	Score
FCFP_2	3	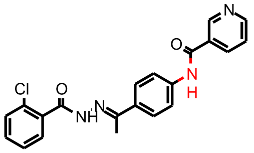 [*]N[*]	0.0737

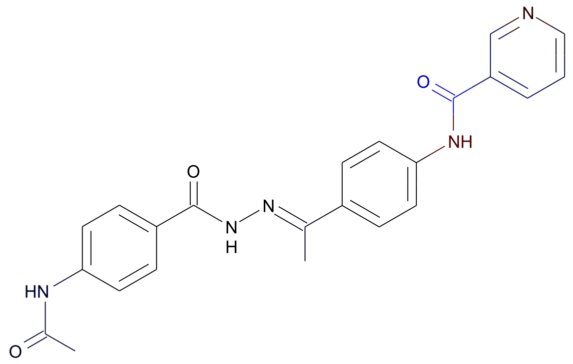

$C_{23}H_{21}N_5O_3$
Molecular Weight: 415.44453
ALogP: 1.418
Rotatable Bonds: 6
Acceptors: 5
Donors: 3

Model Prediction

Prediction: 0.122
Unit: g/kg_body_weight
Mahalanobis Distance: 8.66
Mahalanobis Distance p-value: 0.00407

Mahalanobis Distance: The Mahalanobis distance (MD) is a generalization of the Euclidean distance that accounts for correlations among the X properties. It is calculated as the distance to the center of the training data. The larger the MD, the less trustworthy the prediction.
Mahalanobis Distance p-value: The p-value gives the fraction of training data with an MD greater than or equal to the one for the given sample, assuming normally distributed data. The smaller the p-value, the less trustworthy the prediciton. For highly non-normal X properties (e.g., fingerprints), the MD p-value is wildly inaccurate.

Structural Similar Compounds

Name	FUROSEMIDE	SALICYLAZOSULFAPYRIDINE	DAPSONE
Structure	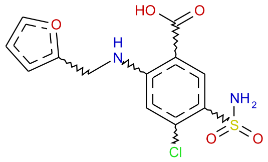	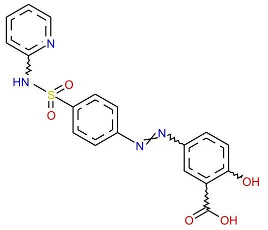	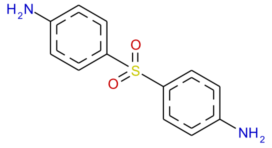
Actual Endpoint (-log C)	4.04236	3.375	3.66258
Predicted Endpoint (-log C)	2.8614	2.80292	3.26993
Distance	0.607	0.681	0.756
Reference	NCI/NTP TR-356	NCI/NTP TR-457	NCI/NTP TR-20

Model Applicability

Unknown features are fingerprint features in the query molecule, but not found or appearing too infrequently in the training set.

1. All properties and OPS components are within expected ranges.

Feature Contribution

Top features for positive contribution

Fingerprint	Bit/Smiles	Feature Structure	Score
FCFP_2	3	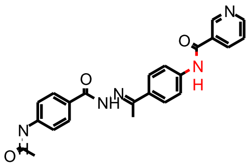 [*]N[*]	0.0737

Sorafenib

TOPKAT_Rat_Maximum_Tolerated_Dose_Feed

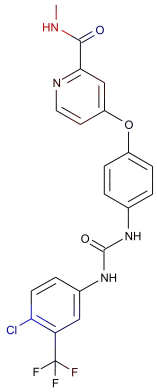

$C_{21}H_{16}ClF_3N_4O_3$

Molecular Weight: 464.82494

ALogP: 4.175

Rotatable Bonds: 6

Acceptors: 4

Donors: 3

Model Prediction

Prediction: 0.0885

Unit: g/kg_body_weight

Mahalanobis Distance: 12.4

Mahalanobis Distance p-value: 1.76e-009

Mahalanobis Distance: The Mahalanobis distance (MD) is a generalization of the Euclidean distance that accounts for correlations among the X properties. It is calculated as the distance to the center of the training data. The larger the MD, the less trustworthy the prediction.
Mahalanobis Distance p-value: The p-value gives the fraction of training data with an MD greater than or equal to the one for the given sample, assuming normally distributed data. The smaller the p-value, the less trustworthy the prediciton. For highly non-normal X properties (e.g., fingerprints), the MD p-value is wildly inaccurate.

Structural Similar Compounds

Name	FUROSEMIDE	PHENOLPHTHALEIN	DISPERSE YELLOW 3
Structure	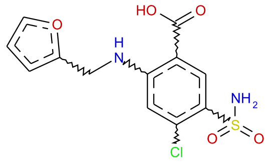	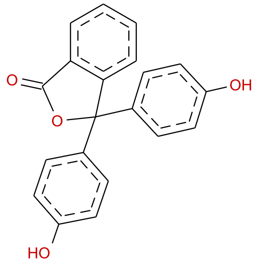	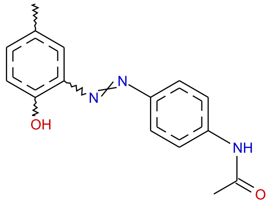
Actual Endpoint (-log C)	4.04236	2.20184	2.77703
Predicted Endpoint (-log C)	2.8614	2.8857	2.80195
Distance	0.741	0.780	0.799
Reference	NCI/NTP TR-356	NCI/NTP TR-465	NCI/NTP TR-222

Model Applicability

Unknown features are fingerprint features in the query molecule, but not found or appearing too infrequently in the training set.

1. All properties and OPS components are within expected ranges.

Feature Contribution

Top features for positive contribution			
Fingerprint	Bit/Smiles	Feature Structure	Score
FCFP_2	-885550502	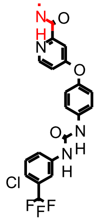 [*]C(=[*])NC	0.115

6 TOPKAT_Rat_Maximum_Tolerated_Dose_Gavage

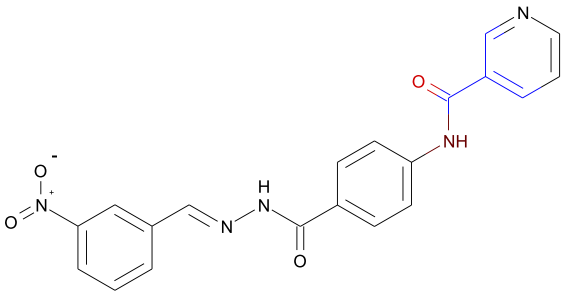

$C_{20}H_{15}N_5O_4$

Molecular Weight: 389.3642

ALogP: 2.211

Rotatable Bonds: 6

Acceptors: 6

Donors: 2

Model Prediction

Prediction: 0.027

Unit: g/kg_body_weight

Mahalanobis Distance: 8.04

Mahalanobis Distance p-value: 0.00184

Mahalanobis Distance: The Mahalanobis distance (MD) is a generalization of the Euclidean distance that accounts for correlations among the X properties. It is calculated as the distance to the center of the training data. The larger the MD, the less trustworthy the prediction.
Mahalanobis Distance p-value: The p-value gives the fraction of training data with an MD greater than or equal to the one for the given sample, assuming normally distributed data. The smaller the p-value, the less trustworthy the prediciton. For highly non-normal X properties (e.g., fingerprints), the MD p-value is wildly inaccurate.

Structural Similar Compounds

Name	OCHRATOXIN	SULFISOOXAZOLE	PENICILLIN VK
Structure	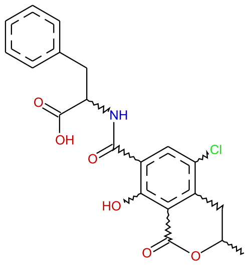	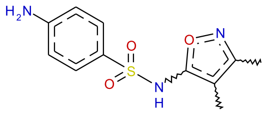	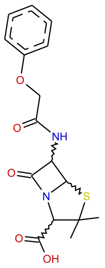
Actual Endpoint (-log C)	6.28396	2.82494	2.54455
Predicted Endpoint (-log C)	5.12358	3.0705	3.9702
Distance	0.765	0.823	0.968
Reference	NCI/NTP TR-358	NCI/NTP TR-138	NCI/NTP TR-336

Model Applicability

Unknown features are fingerprint features in the query molecule, but not found or appearing too infrequently in the training set.

1. Num_AromaticRings out of range. Value: 3. Training min, max, mean, SD: 0, 2, 0.5625, 0.693.
2. Unknown FCFP_2 feature: 8: [*][N+](=[*])[*]
3. Unknown FCFP_2 feature: 5: [*][O-]
4. Unknown FCFP_2 feature: -885520711: [*]C(=[*])NN=[*]
5. Unknown FCFP_2 feature: 581019816: [*]N\N=C\[*]
6. Unknown FCFP_2 feature: -828984032: [*][N+](=[*])[c](:[cH]:[*]):[cH]:[*]
7. Unknown FCFP_2 feature: -1338588315: [*]:[c](:[*])[N+](=O)[O-]
8. Unknown FCFP_2 feature: 1872392852: [*][N+](=O)[*]
9. Unknown FCFP_2 feature: 260476081: [*][N+](=[*])[O-]

Feature Contribution

Top features for positive contribution			
Fingerprint	Bit/Smiles	Feature Structure	Score

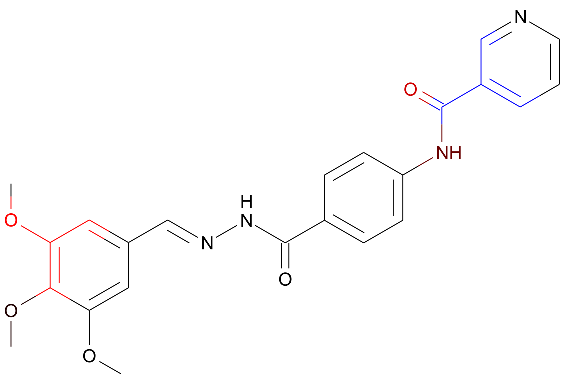

$C_{23}H_{22}N_4O_5$

Molecular Weight: 434.44457

ALogP: 2.268

Rotatable Bonds: 8

Acceptors: 7

Donors: 2

Model Prediction

Prediction: 0.00109

Unit: g/kg_body_weight

Mahalanobis Distance: 9.52

Mahalanobis Distance p-value: 2.52e-005

Mahalanobis Distance: The Mahalanobis distance (MD) is a generalization of the Euclidean distance that accounts for correlations among the X properties. It is calculated as the distance to the center of the training data. The larger the MD, the less trustworthy the prediction.
Mahalanobis Distance p-value: The p-value gives the fraction of training data with an MD greater than or equal to the one for the given sample, assuming normally distributed data. The smaller the p-value, the less trustworthy the prediciton. For highly non-normal X properties (e.g., fingerprints), the MD p-value is wildly inaccurate.

Structural Similar Compounds

Name	OCHRATOXIN	SULFISOOXAZOLE	PENICILLIN VK
Structure	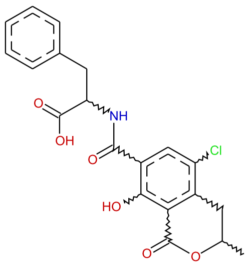	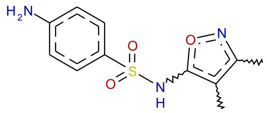	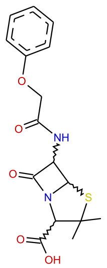
Actual Endpoint (-log C)	6.28396	2.82494	2.54455
Predicted Endpoint (-log C)	5.12358	3.0705	3.9702
Distance	0.829	0.958	0.995
Reference	NCI/NTP TR-358	NCI/NTP TR-138	NCI/NTP TR-336

Model Applicability

Unknown features are fingerprint features in the query molecule, but not found or appearing too infrequently in the training set.

1. Num_H_Acceptors out of range. Value: 7. Training min, max, mean, SD: 0, 6, 1.6146, 1.644.
2. Num_AromaticRings out of range. Value: 3. Training min, max, mean, SD: 0, 2, 0.5625, 0.693.
3. OPS PC6 out of range. Value: -3.2562. Training min, max, SD, explained variance: -2.4321, 2.9885, 1.256, 0.0488.
4. Unknown FCFP_2 feature: -885520711: [*]C(=[*])NN=[*]
5. Unknown FCFP_2 feature: 581019816: [*]N\N=C\[*]

Feature Contribution

Top features for positive contribution

Fingerprint	Bit/Smiles	Feature Structure	Score
FCFP_2	332760439	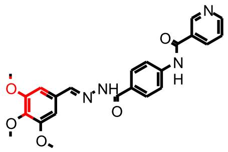 [*]O[c](:[cH]:[*]):[c]([*]):[*]	0.672
